# Evaluation of Azobenzene Ethers Carrying a Perhalogenated Moiety as Halogen Bond Donors by Cocrystallization with Nitrogen-Containing Acceptors

**DOI:** 10.1021/acs.cgd.5c00749

**Published:** 2025-07-28

**Authors:** Filip Kučas, Lidija Posavec, Nikola Bedeković, Vinko Nemec, Dominik Cinčić

**Affiliations:** Department of Chemistry, Faculty of Science, University of Zagreb, Horvatovac 102a, 10000 Zagreb, Croatia

## Abstract

In this work, we synthesized three novel 4-iodotetrafluorophenoxy-azobenzene ethers, which contain different substituents (X = −H, −Cl, −CN) on the opposite side of the molecule in relation to the perhalogenated moiety carrying the iodine atom. To explore the halogen bond donor potential of the prepared compounds, we performed cocrystal screening with a series of nitrogen-containing acceptors: 1,4-diazabicyclo[2.2.2]­octane, 4-dimethylaminopyridine, 2,2’-bipyridine, 4,4’-bipyridine, 4,4’-azopyridine, *N*,*N’*-bis­(pyridin-4-yl)­methylenehydrazine, 1,2-bis­(pyridin-4-yl)­ethane, and 1,2-bis­(pyridin-4-yl)­ethylene. These three azobenzenes were selected in order to investigate how bent molecules carrying a perhalogenated moiety would act as halogen bond donors, as well as how different substituents on a distant part of the molecule could affect the formation of cocrystals. Out of 24 combinations, only 8 experiments yielded cocrystals suitable for single-crystal X-ray diffraction with two out of three azobenzene derivatives (X = −Cl and −CN). Structural analysis revealed that in all obtained cocrystals, the robust interaction is the I···N halogen bond between the azobenzene iodine atom and the acceptor nitrogen atom. A majority of cocrystals feature two donor molecules per one acceptor molecule and display crystal packing based on discrete trimeric halogen-bonded complexes. Only in the case of the 4,4’-bipyridine cocrystal with a 1:1 stoichiometry is the crystal structure based on discrete halogen-bonded dimers. In order to investigate changes in the halogen bond donor ability of the azobenzene derivatives, we have calculated values of the molecular electrostatic potential (MEP) for the DFT-optimized molecular geometries. Calculations showed that the electrostatic potential on the iodine atom only slightly depends on the functional group located on the opposite side of the molecule, with relatively large MEP values (+135 kJ mol^–1^ e^–1^ on average).

## Graphical abstract

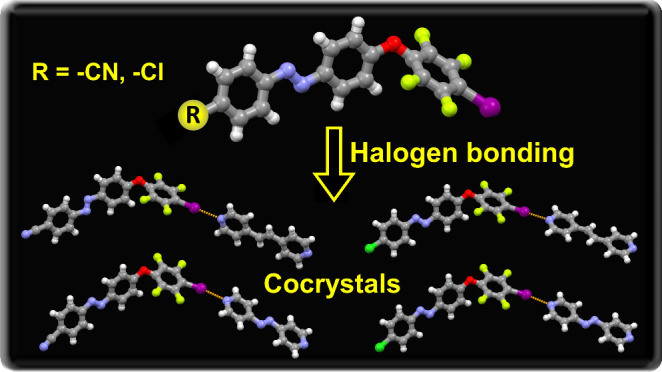

## Introduction

Noncovalent interactions are, in general, an important tool in supramolecular chemistry for the design and synthesis of multicomponent crystals,^1–3^ i.e., such as photoresponsive,^4,5^ conductive,^6^ and magnetic^7^ materials. Among the vast majority of noncovalent interactions, halogen bonding has been intensely investigated throughout the last two decades, becoming the most appropriate σ-hole interaction for applications in the field of supramolecular chemistry and one of the paramount tools in crystal engineering alongside hydrogen bonding.^8–10^ Both hydrogen and halogen bonds are strong and directional interactions with a similar range of bond energies, and both can vary from purely electrostatic to largely covalent.^3,11–14^ Numerous studies have been focused on using (inter)­halogens,^15–17^ unsaturated iodo- (and bromo-) hydrocarbons,^18–20^ *N*-haloimides,^13,21–24^ and other molecules with halogen atoms bonded to an electronegative atom as halogen bond donors in cocrystal design.^25^ Such molecules are generally quite reactive and are therefore difficult to handle and use as building blocks in the crystal engineering of multicomponent crystals. More appropriate for supramolecular chemistry are perfluorohalocarbons, halogen bond donors introduced by Resnati and Metrangolo in the late 1990s.^26–28^ Perfluorinated halobenzenes, in particular pentafluoroiodobenzene, the three isomers of tetrafluorodiiodobenzene, and 1,3,5-trifluorotriiodobenzene, are to date the most widely used in crystal engineering.^8–10,29–33^ Their widespread use is a result of the strong electron-withdrawing effect of fluorine atoms leading to significant donor atom polarization, their lack of competing functional groups for supramolecular interactions and overall stability under commonly used synthetic conditions (e.g. in typical solution crystallization experiments in a number of different solvents, or in mechanochemical synthesis)^34,35^ and their commercial availability. According to the available structural data in the Cambridge Structural Database (CSD),^29^ there are a total of 1880 data sets for crystals containing perfluorinated halobenzenes. Of those, 802 data sets correspond to multicomponent crystals with 1,4-diiodotetrafluorobenzene, 362 data sets are with 1,3,5-triiodotrifluorobenzene, 139 data sets are with iodopentafluorobenzene, 126 data sets are with 1,2-diiodotetrafluorobenzene, and 92 data sets are with 1,3-diiodotetrafluorobenzene. Furthermore, there are 289 data sets for crystals containing the [I–C_6_F_4_-R] motif (R being C, N, or O) that correspond to molecules containing the perfluorinated iodobenzene moiety, which are mostly commercially unavailable. A subset of these data corresponds to crystals containing the [I_*para*_-C_6_F_4_-R] motif, with 255 data sets. Of those, 151 data sets correspond to cocrystals: 71 data sets with the [I_*para*_-C_6_F_4_–C] motif, 52 data sets with the [I_*para*_-C_6_F_4_–N] motif, and 28 data sets with the [I_*para*_-C_6_F_4_–O] motif. While there is a solid amount of data for a general azobenzene motif [Ph-N = N-Ph] with 4559 data sets (Ph being a benzene ring with no restrictions on its substituents), only a small amount of these data exhibited the [Ph-N = N–C_6_F_4_–I] motif, 43 data sets, of which 36 correspond to multicomponent crystals. A subset of these data corresponds to crystals containing the I–C_6_F_4_–N = N–C_6_F_4_–I molecule, with 30 data sets. Due to *cis*/*trans* isomerization of this type of donor, cocrystallization with appropriate acceptors could yield materials that exhibit remarkable photomechanical properties.^36–38^ After narrowing the search to structures containing both the [Ph-N = N-Ph] and [R-C_6_F_4_–I] motifs, we found only two data sets corresponding to peripherally perfluorinated azobenzene ethers in which a perfluorinated unit is attached to an azobenzene unit through an ether linker. These two compounds were reported by Frangville and co-workers and were investigated only in solution.^39^ This overview clearly shows that work including azobenzene moieties in halogen-bonded cocrystals was exclusively done with perfluorinated azobenzenes, while the behavior of peripherally perfluorinated azobenzenes in the solid state remains mostly unexplored.

There are well-established principles for the formation of halogen bonds between molecules and the synthesis of cocrystals; however, numerous studies on halogen-bonded cocrystals showcased the unpredictability of the outcome of cocrystallization experiments (either mechanochemical or by solution crystallization) even though the potential donor molecule carries a group containing a strongly polarized halogen (mostly iodine atoms) and the acceptor molecule contains an appropriate electron-rich region (i.e., a pyridine nitrogen atom). In general, screening suitable coformers is a major challenge during cocrystal synthesis. For instance, in previous work, Aakeröy and co-workers performed 108 experiments by liquid-assisted grinding (LAG) in order to study cocrystallization of 9 perfluorinated halogen bond donors and 12 ditopic nitrogen-containing acceptors.^40^ A total of 89 (82%) experiments resulted in cocrystal formation, but cocrystallization experiments from solution yielded only 35 (32%) crystals suitable for single-crystal X-ray diffraction. In another work, they performed LAG screening of a combination of six aromatic halogen bond donors (either perfluorinated or containing an iodo/bromo-ethynyl moiety) and 21 acceptors.^41^ A total of 58 (46%) of the 126 experiments resulted in cocrystal formation. In a more recent study, Rissanen, Puttreddy, and co-workers attempted cocrystallization of 32 pyridine *N*-oxides as very strong halogen bond acceptors and a selection of five perfluoroiodobenzenes and obtained 111 (69%) cocrystals out of 160 experiments.^42^ In another work, they performed a cocrystal screen of a combination of four diiodoperfluoroalkanes and eight methyl-substituted pyridine *N*-oxides, and obtained 17 cocrystals (53%) out of 32 combinations.^43^ Valkonen and co-workers performed 45 experiments in order to study cocrystallization of 15 thiourea-based acceptors and three perfluoroiodobenzenes, and obtained only 5 cocrystals (11%).^44^ In another work, they performed cocrystallization of two perfluoroiodobenzenes and 24 thiocarbonyl acceptors and obtained 19 cocrystals (40%) out of 48 combinations.^45^ Furthermore, a number of studies of halogen-bonded cocrystallization of perfluorinated benzenes with a wide range of organic and metal–organic acceptors were performed by our group. Out of a total of 178 performed cocrystallization experiments from solution, 111 (62%) yielded cocrystals.^46–55^

In this work we performed cocrystal screening with azobenzene ethers and a series of selected nitrogen containing acceptors: 1,4-diazabicyclo[2.2.2]­octane (**dabco**), 4-dimethylaminopyridine (**dmap**), 2,2’-bipyridine (**22bpy**), 4,4’-bipyridine (**44bpy**), 4,4’-azopyridine (**44azpy**), *N*,*N’*-bis­(pyridin-4-yl)­methylenehydrazine (**hpy**), 1,2-bis­(pyridin-4-yl)­ethane (**dpa**), and 1,2-bis­(pyridin-4-yl)­ethylene (**dpe**), (Scheme 1). For this purpose, we synthesized three 4-iodotetrafluorophenoxy-azobenzene ethers that exhibit “V-shaped” molecular geometry and contain different substituents (−H, −Cl, −CN) on the opposite side of the molecule in relation to the moiety carrying the iodine atom (Scheme 1). We selected these compounds in order to explore how bent molecules carrying a perhalogenated moiety would act as halogen bond donors, as well as how the incorporation of atoms like hydrogen and chlorine and groups like the cyano group on a distant part of the molecule could affect the formation of cocrystals.

**Scheme 1:**
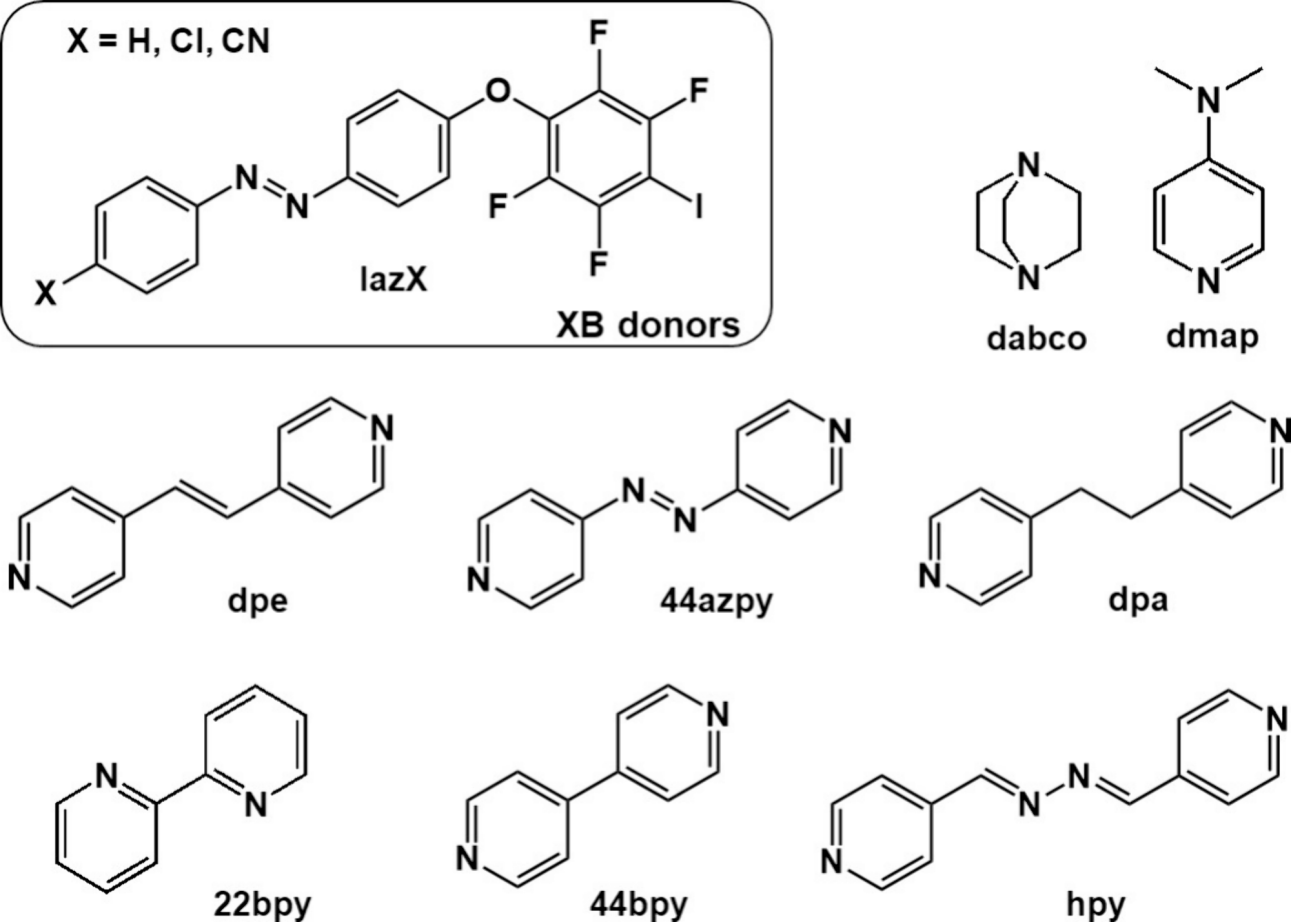
Halogen Bond Donors and Acceptors Used in This Study

## Experimental Section

### Synthesis

All substances except **44azpy**, **hpy**, **IazH**, **IazCl**, and **IazCN** were purchased from commercial sources. The synthesis of halogen bond donors was performed in a two-step process (Scheme 2). In the first step, azo-precursors were synthesized using the azo-coupling reaction of phenol with an appropriate substituted aniline. In the next step, azo-precursors were condensed with iodopentafluorobenzene, thus forming the desired compounds.

**Scheme 2:**
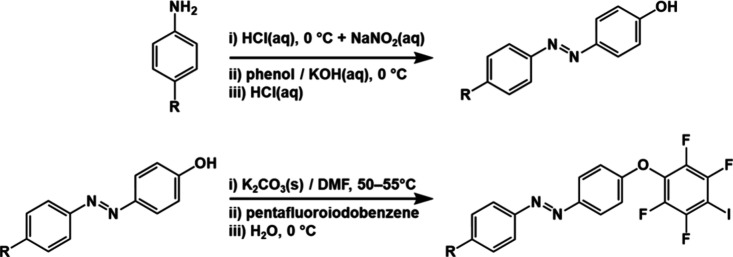
Steps in the Synthesis of 4-Iodotetrafluorophenoxy-azobenzene Ethers

### General Procedure for the Synthesis of Azo-precursors

The substituted aniline (5.0 mmol) was dissolved in hydrochloric acid (6 mL, 2 mol dm^–3^). The prepared solution was cooled to 0 °C; 2 mL of a water solution of sodium nitrite (2 mol dm^–3^) was added, and the solution was stirred for another 5 min. The prepared diazonium salt solution was then added to a solution of phenol (0.476 g, 5.0 mmol) in potassium hydroxide (10 mL, 2 mol dm^–3^) with strong stirring while the temperature was kept at 0 °C. The reaction mixture was stirred for another 40 min, after which dilute hydrochloric acid (10 mL, 2 mol dm^–3^) was added, and the obtained precipitate was filtered off and dried. The obtained azo-precursor was used without further purification.

### General Procedure for the Synthesis of 4-Iodotetrafluorophenoxy-azobenzene Ethers^39^

The prepared azo-precursor (2.5 mmol) was mixed with potassium carbonate (3.25 mmol). The obtained mixture was then suspended in DMF (5 mL) and heated between 50–55 °C. After half an hour, pentafluoroiodobenzene (2.75 mmol) was added to a vigorously stirred solution, and the reaction mixture was stirred for another 6 h. After the reaction was complete, the mixture was cooled to room temperature, and the product was precipitated by the addition of 100 mL of ice-cold water. The colored product was then vacuum filtered, dried, and used without further purification (yields: 46% for **IazH**, 60% for **IazCl**, and 47% for **IazCN**).

### Synthesis of 44azpy

In a 250 mL beaker, a water solution of sodium hypochlorite (6–14%, 60 mL) was cooled to 0 °C using an ice water bath. Then, 25 mL of a cold water solution of 4-aminopyridine (1.18 g, 12.5 mmol) was added dropwise over 45 min, with the temperature of the reaction mixture kept below 5 °C. After the addition, the mixture was stirred for another 1 h. The crude product was obtained via vacuum filtration. The resulting orange precipitate was further recrystallized from *n*-hexane.

### Synthesis of hpy

A water solution of hydrazine hydrate (1 mL, 21.0 mmol) was added dropwise to a solution of pyridine-4-carbaldehyde (4 mL, 40.0 mmol) in 15 mL of absolute ethanol with vigorous stirring. After the addition, the mixture was stirred for another 30 min. The crude product was obtained by vacuum filtration. The resulting yellow precipitate was used without further purification.

### Mechanochemical Cocrystal Screening

Cocrystal screening was performed by ball-milling mixtures of **IazH**, **IazCl**, or **IazCN** with **44bpy**, **44azpy**, **dpe**, **dpa**, and **hpy** in stoichiometric ratios of 2:1. The reaction mixture (60 mg) was placed in a 5 mL stainless steel jar along with 15 μL of acetonitrile and one stainless-steel ball 7 mm in diameter and then milled for 15 min in a Retsch MM200 Shaker Mill operating at 25 Hz. The resulting powders were characterized by powder X-ray diffraction. Details on mechanochemical experiments are given in the ESI.

### Solution-Based Cocrystallization

Cocrystals suitable for single-crystal experiments were prepared by crystallization from a suitable solvent. About 30 mg of a mixture of **IazH**, **IazCl** or **IazCN**, with either **44bpy**, **hpy**, **dpe**, **dpa**, or **44azpy** in a 1:1 stoichiometric ratio, was dissolved in a hot solvent. The crystals were obtained by slow evaporation of the solvent at room temperature after a few days. Details for crystallization experiments are given in the ESI.

### Single-Crystal X-ray Diffraction (SCXRD)

Crystal and molecular structures of eight cocrystals and all three azobenzene-ethers were determined by using single-crystal X-ray diffraction. Diffraction measurements were made on an Oxford Diffraction Xcalibur Kappa CCD X-ray diffractometer with graphite-monochromated Mo*K*α (λ = 0.71073 Å) radiation. The data sets were collected using ω scan mode over the 2θ range up to 54°. All data were collected at 295 K. Programs CrysAlis CCD, CrysAlis RED, and CrysAlisPro were employed for data collection, cell refinement, and data reduction, respectively.^56^ The structures were solved by direct methods and refined using the SHELXT, SHELXS, and SHELXL programs, respectively.^57,58^ Structural refinement was performed on *F* ^2^ using all of the data. The hydrogen atoms were placed in calculated positions and treated as riding on their parent atoms [C–H = 0.93 Å and *U*_iso_(H) = 1.2 *U*_eq_(C); C–H = 0.97 Å and *U*_iso_(H) = 1.2 *U*_eq_(C)]. Crystal data and refinement details are given in the ESI. All calculations were performed using the WINGX crystallographic suite of programs.^59^

### Intermolecular Contact Analysis

Intermolecular contacts were analyzed using Mercury 2022.3.0.^60^ In the determined structures, contacts that are shorter than the sum of the van der Waals radii of the involved atoms were analyzed. Two-dimensional molecular fingerprint plots were calculated and drawn using the *d*_norm_ function of CrystalExplorer 21.5 software.^61^

### Differential Scanning Calorimetry (DSC)

Bulk **s**amples for DSC measurements for both azobenzene ethers and their cocrystals were obtained by solution crystallization or recrystallization experiments. Due to issues with crystal nucleation, all experiments ended with complete evaporation of the solvent, and the crystals were usually scraped off the crystallization vial walls and bottom. The measurements were performed on a Mettler Toledo DSC823^e^ module in sealed aluminum pans (40 μL) with one pinhole in the lid, heated in flowing nitrogen (200 mL min^–1^) at a rate of 10 °C min^–1^. The data collection and analysis were performed using the program package STARe Software 15.00.^62^

### Calculation Details

All calculations were performed using the Gaussian 16 software package.^63^ Geometry optimizations of halogen bond donors and acceptors, as well as MEP calculations, were performed using M06–2X/def2-tzvp level of theory,^64^ with an ultrafine integration grid (99 radial shells and 590 points per shell). The default Gaussian convergence criteria were used. Harmonic frequency calculations were performed on the optimized geometries to ensure that the obtained structures represented true minima. The Figures were prepared using GaussView 5.1.^65^

## Results and Discussion

We synthesized three novel peripherally perfluorinated azobenzene ethers, **IazH**, **IazCl**, and **IazCN**, which are orange solids at room temperature (Figure 1). Structural characterization showed that molecules of **IazCN** are interconnected through I···N_nitrile_ halogen bonds (*d*(I1···N3) = 3.053 Å), thus forming a chain spreading along the diagonal of the *ac* crystallographic plane. The chains are connected into a 2D layer via a combination of an *R*_2_^2^(10)*R*_2_^2^(10) hydrogen bonding motif (*d*(C5···N3) = 3.637 Å, ∠(C5–H5···N3) = 167.0°) and a C–H···F contact (*d*(C6···F2) = 3.488 Å, ∠(C6–H6···F2) = 154.7°). The layers are then closely packed in the 3D crystal structure. On the other hand, in crystals of **IazCl**, we observed interhalogen type I I···Cl contacts (*d*(I1···Cl1) = 3.613 Å), at an angle of 137.1°. These contacts lead to the formation of a chain, and the chains are connected by C–H···F contacts (*d*(C2···F3) = 3.383 Å, ∠(C2–H2···F3) = 161.9°; *d*(C8···F4) = 3.142 Å, ∠(C8–H8···F4) = 144.3°; *d*(C5···F2) = 3.563 Å, ∠(C5–H5···F2) = 160.0°) into a 3D network. Although the **IazH** molecule contains potential halogen bond acceptor sites like the ether oxygen atom, the azo-group as well as two benzene rings, the iodine atom of **IazH** does not engage in halogen bonding with any of these groups, instead **IazH** molecules are interconnected only through C–H···F contacts (*d*(C9···F1) = 3.450 Å, ∠(C9–H9···F1) = 148.4°) into a chain along the *a* crystallographic axis. The chains are then closely packed in the 3D crystal structure.

**Figure 1:**
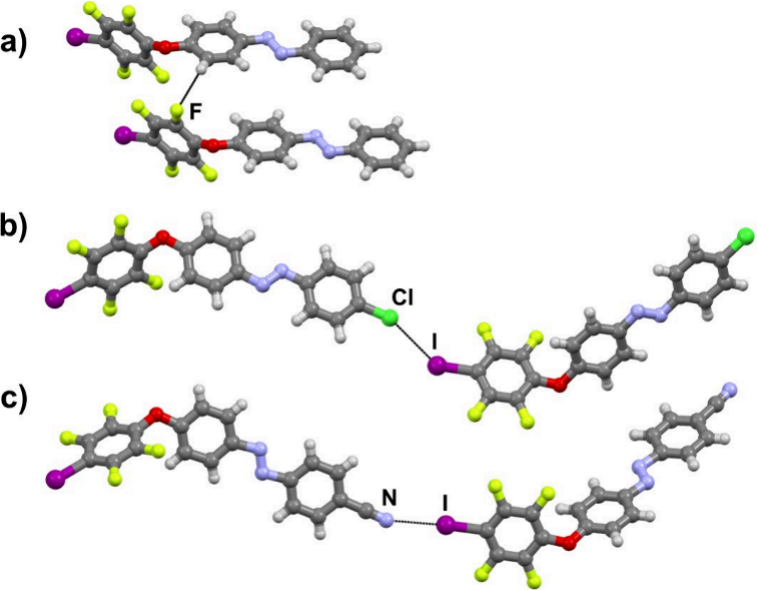
Parts of crystal structures of the herein synthesized azobenzene ethers: (a) **IazH**, (b) **IazCl,** and (c) **IazCN**.

In order to investigate how halogen bond donor and acceptor abilities change in the prepared azobenzene derivatives and the nitrogen-containing molecules used, we have calculated values of the molecular electrostatic potential (MEP) of the DFT-optimized geometries for donor and acceptor molecules. It can be noticed that the electrostatic potential on the iodine atom only slightly depends on the functional group X located on the opposite side of the molecule (the biggest difference in MEPs between **IazH** and **IazCN** is about 8 kJ mol^–1^ e^–1^, Figure 2). This observation is a consequence of the reduced inductive effect of X toward the iodine atom due to their unfavorable relative position in the molecule (they are separated by 16 bonds) and large spatial distance (*d*(I···X) = 16.9 Å on average). Nevertheless, all donors have relatively large MEP values on the donor atoms (MEP = +135 kJ mol^–1^ e^–1^ on average), which implies the possibility of realizing halogen bonds with suitable acceptor species. Regarding the acceptors, they all have negative MEP values on the nitrogen atoms, ranging from −100 kJ mol^–1^ e^–1^ (**22bpy**) to −180 kJ mol^–1^ e^–1^ (**dmap**), which, according to the well-established principles and guidelines for crystal engineering of halogen-bonded materials, should make them all good halogen bond acceptors. Considering the presence of two acceptor sites with negative MEP values and a relatively large distance between them within the acceptor molecules (which significantly reduces the impact of anticooperativity on the binding of second donor molecules), the formation of D_2_A cocrystals is expected with monotopic **IazX** donors.

**Figure 2:**
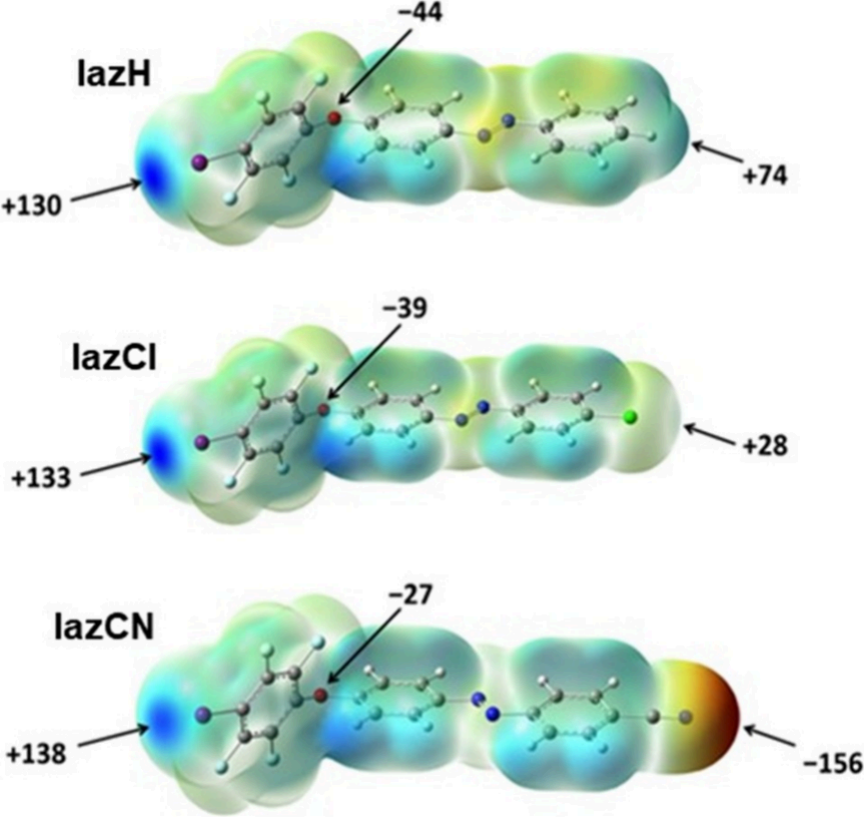
Calculated values in kJ mol^–1^ e^–1^ of the molecular electrostatic potential mapped to the electron density isosurfaces (ρ = 0.001 au) corresponding to the optimized geometries of azobenzene derivatives (M06-2X/def2-tzvp level of theory).

Out of 24 combinations, we obtained a total of 8 cocrystals by cocrystallization experiments from a solution of reactants, with two out of three azobenzene derivatives, **IazCl** and **IazCN**. This revealed several interesting results. In spite of our expectations and calculation results, which both considered the change in substituents on the part of the molecule distant to the activated iodine atom as being negligible, all experiments performed with **IazH** failed to yield any cocrystals at all. We were unable to detect the formation of new crystalline solids, since we predominantly obtained oils and amorphous solids, and only rarely a mixture of reactants. Although we have no evidence to prove whether **IazH** is capable or not of being a halogen bond donor in combination with the used bipyridyl acceptors, it is surprising that no crystalline material was obtained under the same experimental conditions as for the cocrystals obtained with **IazCl** and **IazCN**, considering the minute differences in the relatively large donor molecules. As is evident from the mentioned previous studies, which involved cocrystal screening experiments for systems utilizing well-established halogen bonding donor and acceptor moieties, one can easily infer that the mere possibility of forming a primary robust interaction does not ensure the formation of cocrystals. This is mainly because strong, usually highly directional interactions are important for molecular recognition and in the initial state of crystal nucleation, but the overall cocrystallization outcome also depends on other factors. Weak secondary interactions are a large contributor to the energy of the crystal and also significantly affect the close packing of molecules in the crystal and its thermodynamic stability. Furthermore, for all of the prepared azobenzene ethers, cocrystallization experiments from the solution were accompanied by mechanochemical experiments. However, we obtained only amorphous solids by LAG in all combinations of acceptors and **IazCl**, **IazCN**, or **IazH** (see ESI). This is a rather unusual result, since grinding experiments are often considered a superior method for cocrystal screening, both in the literature as well as in our research experience.^13,23,24,34,35,46,48,49,53,66^ Because of the amorphization of mixtures, we could not detect the formation of any cocrystals.

The third interesting observation in our cocrystal screening is that we only obtained cocrystals with bulkier acceptor molecules, except **22bpy**, which is both a sterically hindered, rod-like molecule and the weakest Lewis base. This result is consistent with a trend that we observed based on data in the CSD for similar and large halogen bond donors mentioned in the introduction. One can find 28 deposited data sets corresponding to cocrystals of I_*para*_-C_6_F_4_–O-R donors. A subset of these data corresponds to crystals containing ditopic bulkier rod-like pyridine derivatives with 23 data sets (77%). Furthermore, we found 71 data sets corresponding to cocrystals of I_*para*_-C_6_F_4_–C-R donors, of which 54 (76%) correspond to cocrystals containing bulkier rod-like pyridine derivatives and only 17 correspond to cocrystals containing monotopic simple pyridine derivatives. For cocrystals of I_*para*_-C_6_F_4_–N-R halogen bond donors, we found 52 data sets, of which 38 (73%) correspond to cocrystals containing bulkier acceptors.

Single-crystal X-ray diffraction analysis showed that in all obtained cocrystals, the robust interaction is the I···N halogen bond. All obtained cocrystals showed a 2:1 halogen bond donor to acceptor ratio, while in the case of the **44bpy** cocrystal, the stoichiometry was 1:1. A very similar halogen-bonded trimer (Figures 3 and 4) is present in all cocrystals, except in the case of the **44bpy** cocrystal, where the molecules form a discrete halogen-bonded dimer.

**Figure 3:**
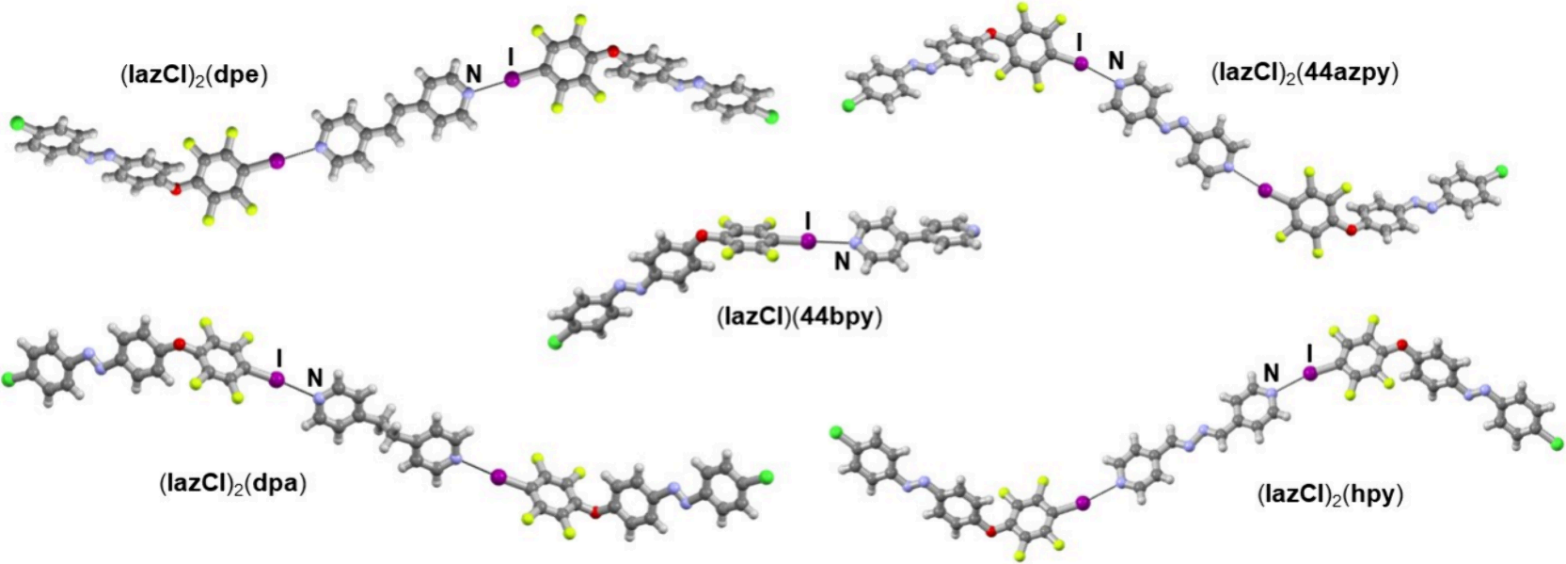
Parts of the crystal structures of **IazCl** cocrystals with bipyridine derivatives.

**Figure 4:**
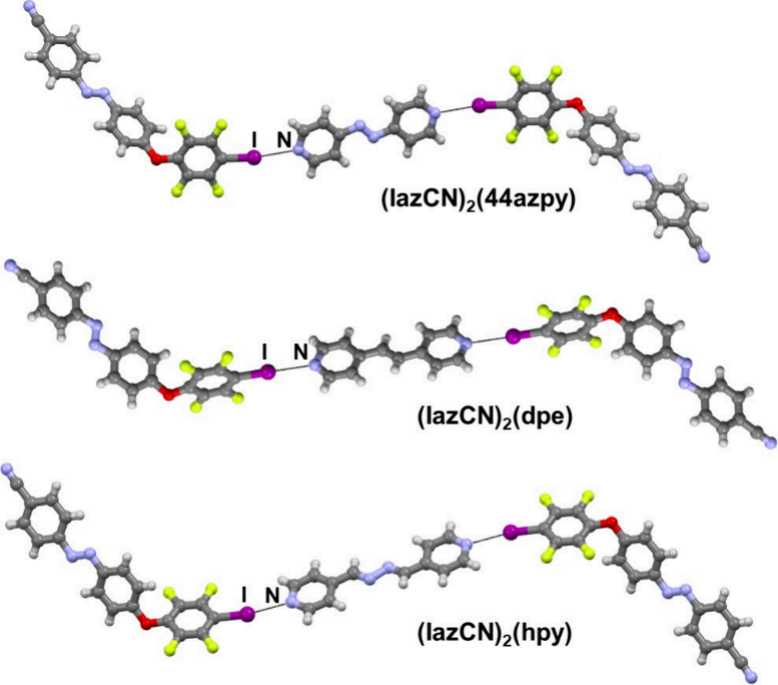
Parts of the crystal structures of **IazCN** cocrystals with bipyridine derivatives.

Cocrystallization of **IazCl** with bipyridyl acceptors resulted in five cocrystals (Figure 3, see Table S5 in the ESI for a full list of directional intermolecular interactions). As mentioned above, the (**IazCl**)­(**44bpy**) cocrystal is different from others in this series since it exhibits a 1:1 donor to acceptor ratio. In the crystal structure, one nitrogen atom of **44bpy** participates in halogen bonding with an iodine atom of the donor molecule, while the other nitrogen atom participates in C–H···N hydrogen bonding with the azobenzene part of the halogen bond donor molecule. Therefore, two molecules of **44bpy** and two molecules of **IazCl** are interconnected through both hydrogen and halogen bonds, thus forming a tetrameric supramolecular motif that has not been observed in cocrystals with other bipyridyl acceptors. The tetramers are connected into a 2D layer by a combination of C–H···F and C–H···C_π_ contacts. The layers are then closely packed in the 3D crystal structure. In all other cocrystals of **IazCl** and bipyridyl acceptors, bipyridyl molecules act as ditopic halogen bond acceptors. Two **IazCl** molecules are bonded to bipyridyl acceptors via two symmetrically equivalent I···N halogen bonds, except in (**IazCl**)_2_(**44azpy**), where there are two symmetrically inequivalent I···N halogen bonds instead. In the crystal structures of (**IazCl**)_2_(**44azpy**) and (**IazCl**)_2_(**dpe**), trimers formed by halogen bonding are then interconnected into a 3D network through a combination of Cl···Cl contacts between peripherally located chlorine atoms and C–H···F contacts. In the (**IazCl**)_2_(**dpa**) crystal structure, trimers are connected into a chain by Cl···Cl contacts and then closely packed in the 3D crystal structure. Interhalogen contacts are absent in the (**IazCl**)_2_(**hpy**) crystal structure, where the trimers are connected to the 3D network solely by C–H···F contacts. Due to inherent similarities in the structure and Lewis basicity of the used halogen bond acceptors, with only the spacer between pyridine rings being different, it comes as no surprise that I···N halogen bonds were similar in length in all **IazCl** cocrystals, with relative shortening values from 17.2% in (**IazCl**)_2_(**44azpy**) to 20.7% in (**IazCl**)_2_(**dpe**). Also, by analyzing data in Table 1, it can be seen that halogen bonds in the **IazCl** series are highly linear, ranging from 169.9° in (**IazCl**)_2_(**hpy**) to 178.3° in (**IazCl**)_2_(**dpe**).

**Table 1:** Halogen Bond Lengths *d*(X···A), Bond Angles α*,* and Relative Shortening RS^a^ of X···A Distances for the Herein Prepared Compounds

compound	D–X···A	*d*(D–X)/Å	*d*(X···A)/Å	α/°	RS^a^/%
**IazH**	-	2.071	-	-	-
**IazCl**	C–I···Cl	2.064	3.613	137.1	3.1
**IazCN**	C–I···N	2.079	3.053	176.2	13.5
(**IazCl**)(**44bpy**)	C–I···N	2.090	2.832	176.8	19.8
(**IazCl**)_2_(**44azpy**)	C16–I1···N5	2.095	2.903	172.1	17.8
	C34–I2···N8	2.091	2.923	170.6	17.2
(**IazCl**)_2_(**dpe**)	C–I···N	2.086	2.801	178.3	20.7
(**IazCl**)_2_(**dpa**)	C–I···N	2.080	2.876	175.0	18.5
(**IazCl**)_2_(**hpy**)	C–I···N	2.096	2.867	169.9	18.8
(**IazCN**)_2_(**44azpy**)	C–I···N	2.089	2.922	171.4	17.2
(**IazCN**)_2_(**dpe**)	C–I···N	2.100	2.952	175.4	16.4
(**IazCN**)_2_(**hpy**)	C–I···N	2.098	2.851	169.9	19.2

a RS = 1 – *d*(D···A)/[*r*_vdW_(D) + *r*_vdW_(A)].^67^

Cocrystallization of **IazCN** with selected acceptors resulted in three cocrystals (Figure 4). Analogously to **IazCl** cocrystals, molecules in these cocrystals are connected by I···N halogen bonds into trimers formed by one bipyridyl molecule and two molecules of **IazCN**. In the (**IazCN**)_2_(**dpe**) and (**IazCN**)_2_(**44azpy**) cocrystals the halogen bonded trimers are then interconnected via a combination of the *R*_2_^2^(10) C–H···N hydrogen bonding motif with nitrile nitrogen atoms and C–H···F contacts into a 3D network, also with additional C–H···N hydrogen bonds in the (**IazCN**)_2_(**dpe**) cocrystal. In the (**IazCN**)_2_(**hpy**) cocrystal, the nitrile functional group does not participate in hydrogen bonding, and therefore, the 3D network is obtained by connecting the trimer solely by C–H···F contacts. As is the case with **IazCl**, cocrystals with **IazCN** feature highly linear halogen bonds ranging from 169.9° in (**IazCl**)_2_(**hpy**) to 175.4° in (**IazCN**)_2_(**dpe**), with relative shortening values from 16.4% for (**IazCN**)_2_(**dpe**) to 20.7% for (**IazCl**)_2_(**dpe**). From a summary of halogen bonding parameters presented in Table 1, it can be observed that for the same halogen bond acceptors, both **IazCl** and **IazCN** form halogen bonds of almost equivalent bond lengths and relative shortening values. This observation also implies that changing chlorido and cyano functional groups on distant parts of a molecule (spacers in our case being one ether oxygen atom and one azo linker group along with an azobenzene ring) has minor effects on geometrical characteristics of halogen bonding, which furthermore implies a minimal effect on halogen bond strength.

From the data in Figure 5, it can be seen that there is a shortening for our data set. The data that exhibit a roughly linear relationship belong to cocrystals of structurally similar bipyridyl molecules **44bpy**, **44azpy**, **dpe**, and **dpa** that have a similar spacer length between the two pyridine rings. Points from Figure 5 that deviate from the observed trend correspond to cocrystals with **hpy**, where the spacer length is much larger, and to the (**IazCN**)_2_(**dpe**) cocrystal in which both *ortho* C–H groups of **dpe** participate in additional C–H···F and C–H···NC hydrogen bonding, thereby preventing a closer approach of the donor atom to the acceptor site and the formation of a shorter halogen bond.

**Figure 5:**
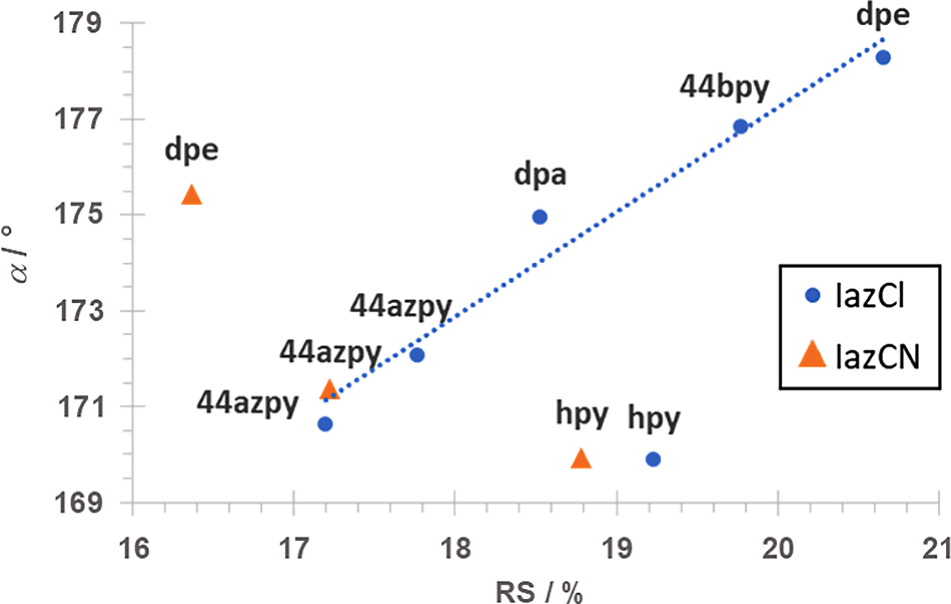
Dependence of halogen bond angle on relative shortening.

In order to quantitatively investigate intermolecular interactions in the obtained cocrystals, we performed Hirshfeld surface analysis (HSA). Percentages for individual intermolecular interactions of **IazCl** and **IazCN** molecules in the obtained cocrystals are shown in Table S4 (see ESI). From these data, it is possible to observe a remarkable similarity in percentages of I···X (X = N, F, C, H, and O) contacts in cocrystals with the same acceptor molecule. This similarity between contacts formed with iodine indicates that changing electron-withdrawing substituents located peripherally at a large distance from the iodine atom has a negligible effect on the formation of intermolecular contacts. A closer examination of data in Table S4 also reveals that the percentage of I···N halogen bonding in all obtained cocrystals falls in a range between 1.7 and 3.7%, unsurprising considering the structural similarities of XB acceptor molecules. These similarities in interaction types are well represented by the two-dimensional fingerprint plots (Figure 6). Cocrystals with the same halogen bond acceptor molecules have similar fingerprint plots, with the only notable differences observable in the **dpe** cocrystal fingerprint plots. Additionally, similarities in donor fingerprint plots can be observed in cocrystals of different bipyridyl acceptors with the same halogen bond donor, which implies that the overall electron density around azobenzene remains the same.

**Figure 6:**
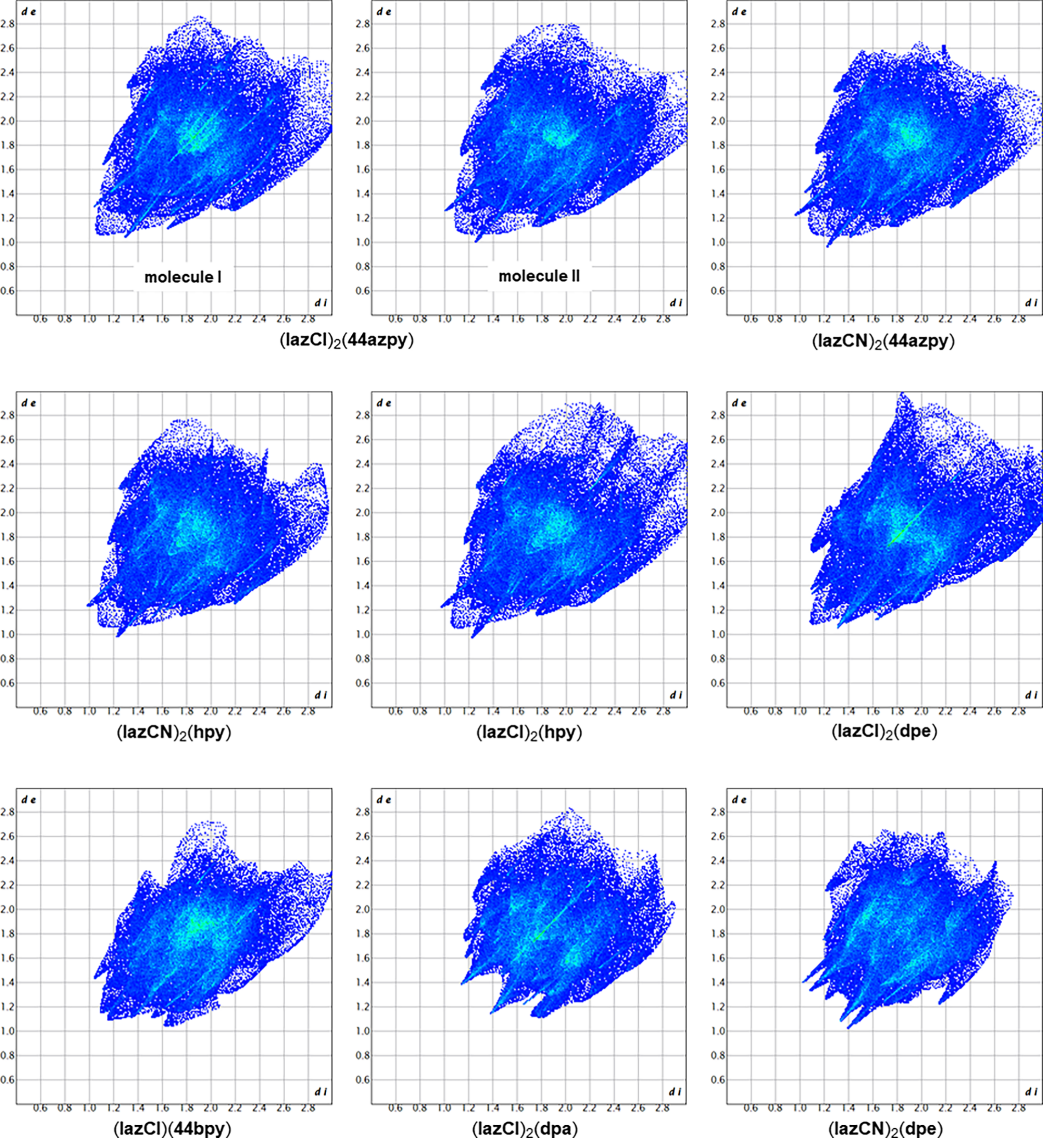
Two-dimensional fingerprint plots of donor molecules in the obtained cocrystals.

Thermal analysis using the DSC method was performed in order to ascertain the thermal stabilities of precursor compounds **IazH**, **IazCl**, and **IazCN**, and their cocrystals (Table 2). Additionally, the method was used to determine the phase purity of the compounds obtained from solution experiments that also yielded single crystals. Due to relatively poor nucleation properties in these systems, all bulk materials were obtained after complete (or near-complete) evaporation of the solvents used. Therefore, for cocrystallization experiments, it could be expected that some of the solid products, in the case of lower supramolecular yield, even when the reactants were mixed in a stoichiometric ratio, would contain a mixture of cocrystal and unreacted reactants. Indeed, only two cocrystals, (**IazCl**)­(**44bpy**) and (**IazCl**)_2_(**44azpy**), feature either one sharp endothermic signal (melting) or a sharp endothermic signal that is composite with a broader one (melting accompanied by decomposition) and could therefore be treated as pure compounds, also indicating high supramolecular yield. The other cocrystals were not obtained as pure phases, indicating lower supramolecular yield. Despite this, we can conclude that the prepared cocrystals are generally thermally stable up to at least 120 °C, and that cocrystals of **IazCN** are more thermally stable than those of **IazCl** cocrystals. **IazCl** cocrystals, with the exception of (**IazCl**)­(**44bpy**), are stable up to at least 140 °C, while **IazCN** cocrystals are stable at least up to 160 °C. This discrepancy in thermal stabilities can be explained by the type of contacts that different groups on azobenzene (in this work–Cl and–CN) form, since no simple trend can otherwise be found when comparing coformer melting and decomposition points to those of their cocrystals. Supramolecular trimers of **IazCl** are connected through weak Cl···Cl interactions, while supramolecular trimers formed from **IazCN** are connected through much stronger C_phenyl_–H···N_nitrile_ contacts, and this could be the cause for the much higher melting points of **IazCN** cocrystals.

**Table 2:** Temperatures at which the Obtained Azobenzene Ethers, Acceptors, and Their Co-Crystals Show a Significant Endothermic Signal, as Determined by DSC Experiments

compound	*t*_endo_/°C	compound	*t*_endo_/°C
**IazH**	162.9	(**IazCl**)(**44bpy**)	131.9
**IazCl**	141.5	(**IazCl**)_2_(**44azpy**)	147.2
**IazCN**	208.5	(**IazCl**)_2_(**hpy**)	148.0
**44bpy**	68.9; 112.0	(**IazCl**)_2_(**dpe**)	149.8
**44azpy**	97.2	(**IazCl**)_2_(**dpa**)	146.2
**hpy**	183.2	(**IazCN**)_2_(**44azpy**)	162.7
**dpe**	151.3	(**IazCN**)_2_(**hpy**)	168.3
**dpa**	111.0	(**IazCN**)_2_(**dpe**)	171.1

## Conclusions

To conclude, in this work, we have successfully synthesized three novel peripherally perfluorinated azobenzene ethers with three different substituents (−H, −Cl, −CN) on the azobenzene unit. In order to investigate the potential of these novel large building blocks as halogen bond donors, we performed a series of cocrystallization experiments with nitrogen-containing acceptors and calculated the molecular electrostatic potential values on their optimized geometries. Altogether, we obtained eight new cocrystals with two azobenzene ethers, **IazCl** and **IazCN**. In all cocrystal structures, the expected I···N halogen bonds are present between the azobenzene iodine atom and a bipyridine derivative nitrogen atom. Out of 8 cocrystals, 7 cocrystals display crystal packing based on discrete trimeric halogen-bonded complexes. Hirshfeld fingerprint plots show that the overall electron density around the azobenzene molecule is similar, and additionally, the **IazCl** cocrystals almost perfectly fit the expectation that shorter halogen bonds are also more linear. Our study revealed several interesting results: (i) It was determined that **IazH** does not behave as a halogen bond donor in its crystal structure as a pure compound. Furthermore, since we were not able to obtain crystal products that could be characterized, we have no evidence of whether **IazH** is capable or not of being a halogen bond donor in combination with the used nitrogen-containing acceptors. Considering the minute differences in the relatively bulky donor molecules, it is surprising that no crystalline material was obtained under the same experimental conditions as for the **IazCN** and **IazCl** cocrystals; (ii) We obtained only amorphous solids by LAG in all combinations of acceptors and **IazCl**, **IazCN**, or **IazH.** This is a rather unusual result, since grinding experiments are often considered a superior method for cocrystal screening; and (iii) We obtained cocrystals only with bulkier acceptor molecules, which is consistent with a trend that we observed based on data in the CSD. Finally, based on our data, we can presume that the presence of an electron-rich functional group on the azobenzene unit, on the opposite side of the molecule in relation to perhalogenated moiety, either (i) makes the cocrystals of the prepared azobenzene ethers more stable in comparison to pure crystal phases enabling weak secondary interactions which contribute largely to the dense packing of molecules in the cocrystal and its stability, or (ii) somewhat enhances the nucleation properties of the cocrystal phase, thus yielding a crystalline product as opposed to mostly obtained amorphous solids or oils.

## Supplementary Material



## Notes

Experimental and computational details, including DSC curves, PXRD patterns and single crystal diffraction data (DOCX)

## References

[1] Desiraju G. R. (1995). Supramolecular
Synthons in Crystal Engineering–A New Organic Synthesis. Angew. Chem., Int. Ed. Engl..

[2] Desiraju G. R. (2007). Crystal
Engineering: A Holistic View. Angew. Chem.,
Int. Ed. Engl..

[3] Gilli, P. ; Gilli, G. ; Noncovalent Interactions in Crystals in Supramolecular Chemistry: From Molecules to Nanomaterials; John Wiley & Sons, Ltd., Online, 2012.

[4] Borchers T. H., Topić F., Christopherson J. C., Bushuyev O. S., Vainauskas J., Titi H. M., Friščić T., Barrett C. J. (2022). Cold photo-carving of halogen-bonded co-crystals of
a dye and a volatile co-former using visible light. Nat. Chem..

[5] Liu Y., Li A., Xu S., Xu W., Liu Y., Tian W., Xu B. (2020). Reversible Luminescent Switching
in an Organic Cocrystal: Multi-Stimuli-Induced
Crystal-to-Crystal Phase Transformation. Angew.
Chem., Int. Ed..

[6] Fourmigué M., Batail P. (2004). Activation of Hydrogen- and Halogen-Bonding Interactions
in Tetrathiafulvalene-Based Crystalline Molecular Conductors. Chem. Rev..

[7] Jeon I.-R., Mathonière C., Clérac R., Rouzières M., Jeannin O., Trzop E., Collet E., Fourmigué M. (2017). Photoinduced
reversible spin-state switching of an Fe^III^ complex assisted
by a halogen-bonded supramolecular network. Chem. Commun..

[8] Cavallo G., Metrangolo P., Milani R., Pilati T., Priimagi A., Resnati G., Terraneo G. (2016). The Halogen Bond. Chem. Rev..

[9] Metrangolo P., Meyer F., Pilati T., Resnati G., Terraneo G. (2008). Halogen Bonding
in Supramolecular Chemistry. Angew. Chem., Int.
Ed..

[10] Metrangolo P., Neukirch H., Pilati T., Resnati G. (2005). Halogen Bonding Based
Recognition Processes: A World Parallel to Hydrogen Bonding. Acc. Chem. Res..

[11] Politzer P., Murray J. S., Clark T. (2010). Halogen bonding: an electrostatically-driven
highly directional noncovalent interaction. Phys. Chem. Chem. Phys..

[12] Eraković M., Cinčić D., Molčanov K., Stilinović V. (2019). A Crystallographic
Charge Density Study of the Partial Covalent Nature of Strong N···Br
Halogen Bonds. Angew. Chem., Int. Ed..

[13] Stilinović V., Horvat G., Hrenar T., Nemec V., Cinčić D. (2017). Halogen and
Hydrogen Bonding between (N-Halogeno)-succinimides and Pyridine Derivatives
in Solution, the Solid State and In Silico. Chem. - Eur. J..

[14] Li D., Xia T., Feng W., Cheng L. (2021). Revisiting the covalent nature of
halogen bonding: a polarized three-center four-electron bond. RSC Adv..

[15] Montis R., Arca M., Aragoni M. C., Blake A. J., Castellano C., Demartin F., Isaia F., Lippolis V., Pintus A., Lenardão E. J., Perin G., O’Connor A. E., Thurow S. (2018). New J. Chem..

[16] d’Agostino S., Braga D., Grepioni F., Taddei P. (2014). Intriguing Case of
Pseudo-Isomorphism between Chiral and Racemic Crystals of rac- and
(*S*)/(*R*)­2-(1,8-Naphthalimido)-2-quinuclidin-3-yl,
and Their Reactivity Toward I_2_ and IBr. Cryst. Growth Des..

[17] Skabara P. J., Bricklebank N., Berridge R., Long S., Light M. E., Coles S. J., Hursthouse M. B. (2000). Crystal engineering towards highly
ordered polymeric structures of 1,3-dithiole-2-thione–dihalogen
adducts. J. Chem. Soc., Dalton Trans..

[18] Aakeröy C. B., Wijethunga T. K., Desper J., Đaković M. (2015). Crystal Engineering
with Iodoethynylnitrobenzenes: A Group of Highly Effective Halogen-Bond
Donors. Cryst. Growth Des..

[19] Sun A., Lauher J. W., Goroff N. S. (2006). Preparation
of Poly­(diiododiacetylene),
an Ordered Conjugated Polymer of Carbon and Iodine. Science.

[20] Lieffrig J., Yamamoto H. M., Kusamoto T., Cui H., Jeannin O., Fourmigué M., Kato R. (2011). Halogen-Bonded, Eight-fold PtS-Type
Interpenetrated Supramolecular Network. A Study toward Redundant and
Cross-Bar Supramolecular Nanowire Crystal. Cryst.
Growth Des..

[21] Raatikainen K., Rissanen K. (2011). Interaction
between amines and N-haloimides: a new
motif for unprecedentedly short Br···N and I···N
halogen bonds. CrystEngComm.

[22] Makhotkina O., Lieffrig J., Jeannin O., Fourmigué M., Aubert E., Espinosa E. (2015). Cocrystal or Salt:
Solid State-Controlled
Iodine Shift in Crystalline Halogen-Bonded Systems. Cryst. Growth Des..

[23] Mavračić J., Cinčić D., Kaitner B. (2016). Halogen bonding of *N*-bromosuccinimide by grinding. CrystEngComm.

[24] Eraković M., Nemec V., Lež T., Porupski I., Stilinović V., Cinčić D. (2018). Halogen Bonding
of *N*-Bromophthalimide
by Grinding and Solution Crystallization. Cryst.
Growth Des..

[25] Troff R. W., Mäkelä T., Topić F., Valkonen A., Raatikainen K., Rissanen K. (2013). Alternative Motifs for Halogen Bonding. Eur. J. Org. Chem..

[26] Corradi E., Meille S. V., Messina M. T., Metrangolo P., Resnati G. (1999). Perfluorocarbon-hydrocarbon self-assembly. Part 6:1
α,ω-Diiodoperfluoroalkanes as pseudohalogens in supramolecular
synthesis. Tetrahedron Lett..

[27] Amico V., Meille S. V., Corradi E., Messina M. T., Resnati G. (1998). Perfluorocarbon–Hydrocarbon
Self-Assembling. 1D Infinite Chain Formation Driven by Nitrogen···Iodine
Interactions. J. Am. Chem. Soc..

[28] Metrangolo P., Resnati G. (2001). Halogen Bonding: A
Paradigm in Supramolecular Chemistry. Chem.
- Eur. J..

[29] Groom C. R., Bruno I. J., Lightfoot M. P., Ward S. C. (2016). The Cambridge Structural
Database. Acta Crystallogr. Sect. B Struct.
Sci. Cryst. Eng. Mater..

[30] Pfrunder M. C., Micallef A. S., Rintoul L., Arnold D. P., Davy K. J. P., McMurtrie J. (2012). Exploitation of the Menshutkin Reaction
for the Controlled
Assembly of Halogen Bonded Architectures Incorporating 1,2-Diiodotetrafluorobenzene
and 1,3,5-Triiodotrifluorobenzene. Cryst. Growth
Des..

[31] Ding X.-H., Chang Y.-Z., Ou C.-J., Lin J.-Y., Xie L.-H., Huang W. (2020). Halogen bonding in
the co-crystallization of potentially ditopic
diiodotetrafluorobenzene: a powerful tool for constructing multicomponent
supramolecular assemblies. National Science
Review.

[32] Bedeković N., Stilinović V., Friščić T., Cinčić D. (2018). Comparison of isomeric meta- and para-diiodotetrafluorobenzene
as halogen bond donors in crystal engineering. New J. Chem..

[33] Nemec V., Lisac K., Bedeković N., Fotović L., Stilinović V., Cinčić D. (2021). Crystal engineering
strategies towards halogen-bonded metal–organic multi-component
solids: salts, cocrystals and salt cocrystals. CrystEngComm.

[34] Cinčić D., Friščić T., Jones W. (2008). Isostructural Materials
Achieved by Using Structurally Equivalent Donors and Acceptors in
Halogen-Bonded Cocrystals. Chem. - Eur. J..

[35] Lisac K., Cepić S., Herak M., Cinčić D. (2022). Halogen-Bonded
Co-Crystals Containing Mono- and Dinuclear Metal-Organic Units: Three-Component
One-Pot Mechanosynthesis, Structural Analysis and Magnetic Properties. Chem.:Methods.

[36] Christopherson J. C., Topić F., Barrett C. J., Friščić T. (2018). Halogen-Bonded
Cocrystals as Optical Materials: Next-Generation Control over Light–Matter
Interactions. Cryst. Growth Des..

[37] Bushuyev O. S., Friščić T., Barrett C. J. (2016). Controlling Dichroism
of Molecular Crystals by Cocrystallization. Cryst. Growth Des..

[38] Bushuyev O. S., Tomberg A., Friščić T., Barrett C. J. (2013). Shaping Crystals with Light: Crystal-to-Crystal Isomerization
and Photomechanical Effect in Fluorinated Azobenzenes. Cryst. Growth Des..

[39] Frangville P., Kumar S., Gelbcke M., Van Hecke K., Meyer F. (2021). Stimuli Responsive Materials Supported by Orthogonal Hydrogen and
Halogen Bonding or I···Alkene Interaction. Molecules.

[40] Aakeröy C. B., Wijethunga T. K., Desper J., Đaković M. (2016). Electrostatic
Potential Differences and Halogen-Bond Selectivity. Cryst. Growth Des..

[41] Aakeröy C. B., Baldrighi M., Desper J., Metrangolo P., Resnati G. (2013). Supramolecular Hierarchy among Halogen-Bond Donors. Chem. - Eur. J..

[42] Rautiainen J. M., Valkonen A., Lundell J., Rissanen K., Puttreddy R. (2024). The Geometry
and Nature of C—I···O—N Interactions
in Perfluoroiodobenzene-Pyridine N-oxide Halogen-Bonded Complexes. Adv. Sci..

[43] Puttreddy R., Topić F., Valkonen A., Rissanen K. (2017). Halogen-Bonded Co-Crystals
of Aromatic N-oxides: Polydentate Acceptors for Halogen and Hydrogen
Bonds. Crystals.

[44] Happonen L., Mattila M., Peshev I., Lehikoinen A., Valkonen A. (2023). Thiourea-Based Tritopic Halogen-Bonding Acceptors. Chem. - Asian J..

[45] Happonen L., Rautiainen J. M., Valkonen A. (2021). Halogen Bonding between Thiocarbonyl
Compounds and 1,2- and 1,4-Diiodotetrafluorobenzenes. Cryst. Growth Des..

[46] Nemec V., Sušanj R., Baus Topić N., Cinčić D. (2025). Competition
vs. Cooperativity of I···O_morpholinyl_ and
I···Cl–M Halogen Bonds in Cocrystals of Zinc­(II)
and Copper­(II) Coordination Compounds Carrying Multiple Acceptor Sites. Chem. - Asian J..

[47] Sušanj R., Bedeković N., Cerovski S., Baus Topić N., Nemec V., Cinčić D. (2024). Evaluation of halogen
bonding proclivity of oxazole derivatives carrying multiple acceptor
sites in cocrystals with perfluorinated iodobenzenes. CrystEngComm.

[48] Kučas F., Posavec L., Nemec V., Bedeković N., Cinčić D. (2023). 2,2′-Bipyridine Derivatives as Halogen Bond
Acceptors in Multicomponent Crystals. Cryst.
Growth Des..

[49] Nemec V., Cinčić D. (2022). The Halogen Bonding Proclivity of the sp^3^ Sulfur Atom as a Halogen Bond Acceptor in Cocrystals of Tetrahydro-4H-thiopyran-4-one
and Its Derivatives. Cryst. Growth Des..

[50] Bedeković N., Piteša T., Eraković M., Stilinović V., Cinčić D. (2022). Anticooperativity
of Multiple Halogen Bonds and Its
Effect on Stoichiometry of Cocrystals of Perfluorinated Iodobenzenes. Cryst. Growth Des..

[51] Uran E., Fotović L., Bedeković N., Stilinović V., Cinčić D. (2021). The Amine Group as Halogen Bond Acceptor in Cocrystals
of Aromatic Diamines and Perfluorinated Iodobenzenes. Crystals.

[52] Nemec V., Lisac K., Liović M., Brekalo I., Cinčić D. (2020). Exploring
the Halogen-Bonded Cocrystallization Potential of a Metal-Organic
Unit Derived from Copper­(II) Chloride and 4-Aminoacetophenone. Materials.

[53] Nemec V., Piteša T., Friščić T., Cinčić D. (2020). The Morpholinyl
Oxygen Atom as an Acceptor Site for
Halogen Bonded Cocrystallization of Organic and Metal–Organic
Units. Cryst. Growth Des..

[54] Carletta A., Zbačnik M., Van Gysel M., Vitković M., Tumanov N., Stilinović V., Wouters J., Cinčić D. (2018). Playing with
Isomerism: Cocrystallization of Isomeric *N*-Salicylideneaminopyridines
with Perfluorinated Compounds as Halogen Bond Donors and Its Impact
on Photochromism. Cryst. Growth Des..

[55] Carletta A., Zbačnik M., Vitković M., Tumanov N., Stilinović V., Wouters J., Cinčić D. (2018). Playing with Isomerism:
Halogen-bonded cocrystals of N-salicylidene Schiff bases and iodoperfluorinated
benzenes: hydroxyl oxygen as a halogen bond acceptor. CrystEngComm.

[56] Rigaku Oxford Diffraction; Gemini CCD System, CrysAlis Pro Software, Version 171.41.93a, 2020.

[57] Sheldrick G. M. (2008). A short
history of SHELX. Acta Cryst. A.

[58] Sheldrick G. M. (2015). Crystal
structure refinement with SHELXL. Acta Cryst.
A.

[59] Farrugia L. J. (2012). J. Appl. Crystallogr..

[60] Macrae C. F., Bruno I. J., Chisholm J. A., Edgington P. R., McCabe P., Pidcock E., Rodriguez-Monge L., Taylor R., Streek J. v. d., Wood P. A. (2008). Mercury CSD 2.0
- new features for the visualization and investigation of crystal
structures. J. Appl. Crystallogr..

[61] Spackman P. R., Turner M. J., McKinnon J. J., Wolff S. K., Grimwood D. J., Jayatilaka D., Spackman M. A. (2021). CrystalExplorer:
a program for Hirshfeld
surface analysis, visualization and quantitative analysis of molecular
crystals. J. Appl. Crystallogr..

[62] STARe Evaluation Software Version 15.00; Mettler–Toledo GmbH, 2016.

[63] Frisch, M. J. ; Trucks, G. W. ; Schlegel, H. B. ; Scuseria, G. E. ; Robb, M. A. ; Cheeseman, J. R. ; Scalmani, G. ; Barone, V. ; Petersson, G. A. ; Nakatsuji, H. ; Li, X. ; Caricato, M. ; Marenich, A. V. ; Bloino, J. ; Janesko, B. G. ; Gomperts, R. ; Mennucci, B. ; Hratchian, H. P. ; Ortiz, J. V. ; Izmaylov, A. F. ; Sonnenberg, J. L. ; Williams-Young, D. ; Ding, F. ; Lipparini, F. ; Egidi, F. ; Goings, J. ; Peng, B. ; Petrone, A. ; Henderson, T. ; Ranasinghe, D. ; Zakrzewski, V. G. ; Gao, J. ; Rega, N. ; Zheng, G. ; Liang, W. ; Hada, M. ; Ehara, M. ; Toyota, K. ; Fukuda, R. ; Hasegawa, J. ; Ishida, M. ; Nakajima, T. ; Honda, Y. ; Kitao, O. ; Nakai, H. ; Vreven, T. ; Throssell, K. ; Montgomery, Jr., J. A. ; Peralta, J. E. ; Ogliaro, F. ; Bearpark, M. J. ; Heyd, J. J. ; Brothers, E. N. ; Kudin, K. N. ; Staroverov, V. N. ; Keith, T. A. ; Kobayashi, R. ; Normand, J. ; Raghavachari, K. ; Rendell, A. P. ; Burant, J. C. ; Iyengar, S. S. ; Tomasi, J. ; Cossi, M. ; Millam, J. M. ; Klene, M. ; Adamo, C. ; Cammi, R. ; Ochterski, J. W. ; Martin, R. L. ; Morokuma, K. ; Farkas, O. ; Foresman, J. B. ; Fox, D. J. Gaussian 16, Revision C.01; Gaussian, Inc.: Wallingford CT, 2016.

[64] Zhao Y., Truhlar D. G. (2008). The M06 suite of density functionals for main group
thermochemistry, thermochemical kinetics, noncovalent interactions,
excited states, and transition elements: Two new functionals and systematic
testing of four M06-class functionals and 12 other functionals. Theor. Chem. Acc..

[65] Dennington, R. ; Keith, T. A. ; Millam, J. M. (Eds.) GaussView, Version 5.1; Semichem Inc.: Shawnee, KS, USA, 2008.

[66] Friščić T., Jones W. (2009). Recent Advances in Understanding the Mechanism of Cocrystal Formation
via Grinding. Cryst. Growth Des..

[67] Bondi A. (1964). Van der Waals
Volumes and Radii. J. Phys. Chem..

