# Supplementary material for: Evaluation of Azobenzene Ethers Carrying a Perhalogenated Moiety as Halogen Bond Donors by Cocrystallization with Nitrogen-Containing Acceptors
Source: Cryst Growth Des. 2025 Jul 28;25(16):6810–9. doi: 10.1021/acs.cgd.5c00749 (PMC12372885; doi:10.1021/acs.cgd.5c00749)
Supplement: Supplementary file 1 [file cg5c00749_si_001.docx]

**SUPPORTING INFORMATION**

**Evaluation of Azobenzene Ethers Carrying a Perhalogenated Moiety as Halogen Bond Donors by Cocrystallization with Nitrogen-containing Acceptors**

Filip Kučas^a^, Lidija Posavec^a^, Nikola Bedeković^a^, Vinko Nemec^a^*, Dominik Cinčić^a^*

^a^ Department of Chemistry, Faculty of Science, University of Zagreb, Horvatovac 102a,
HR-10000 Zagreb, Croatia

Email: vnemec@chem.pmf.hr, dominik@chem.pmf.hr

Fax: +385 1 4606 341

Tel: +385 1 4606 362

**Table of Contents**

| Experimental details | Mechanochemical experiments, solution syntheses, thermal analysis, powder X-ray diffraction experiments, single crystal X-ray diffraction experiments | 5–9 |
| --- | --- | --- |
|  |  |  |
| Table S1 | Experimental data for mechanochemical experiments. Reaction mixtures were placed in 10 mL stainless steel jars along with 10 μL of acetonitrile and one stainless steel ball 7 mm in diameter. The reaction mixtures were then milled in a Retsch MM200 Shaker Mill operating at 25 Hz | 5 |
|  |  |  |
| Table S2 | Experimental data for solution experiments. Reaction mixtures were carefully heated to dissolve the reactants, if necessary, and then left to crystallize at room temperature. | 6–8 |
|  |  |  |
| Table S3 | Crystal data and refinement details for the prepared compounds. | 10–15 |
|  |  |  |
| Figure S1 | Molecular structure of IazH showing the atom-labeling scheme. Displacement ellipsoids are drawn at the 50 % probability level, and H atoms are shown as small spheres of arbitrary radius. | 16 |
|  |  |  |
| Figure S2 | Molecular structure of IazCl showing the atom-labeling scheme. Displacement ellipsoids are drawn at the 50 % probability level, and H atoms are shown as small spheres of arbitrary radius. | 16 |
|  |  |  |
| Figure S3 | Molecular structure of IazCN showing the atom-labeling scheme. Displacement ellipsoids are drawn at the 50 % probability level, and H atoms are shown as small spheres of arbitrary radius. | 17 |
|  |  |  |
| Figure S4 | Molecular structure of (IazCl)(44bpy) showing the atom-labeling scheme. Displacement ellipsoids are drawn at the 50 % probability level, and H atoms are shown as small spheres of arbitrary radius. | 17 |
|  |  |  |
| Figure S5 | Molecular structure of (IazCl)_2_(44azpy) showing the atom-labeling scheme. Displacement ellipsoids are drawn at the 50 % probability level, and H atoms are shown as small spheres of arbitrary radius. | 18 |
|  |  |  |
| Figure S6 | Molecular structure of (IazCl)_2_(dpe) showing the atom-labeling scheme. Displacement ellipsoids are drawn at the 50 % probability level, and H atoms are shown as small spheres of arbitrary radius. | 18 |
|  |  |  |
| Figure S7 | Molecular structure of (IazCl)_2_(hpy) showing the atom-labeling scheme. Displacement ellipsoids are drawn at the 50 % probability level, and H atoms are shown as small spheres of arbitrary radius. | 19 |
|  |  |  |
| Figure S8 | Molecular structure of (IazCl)_2_(dpa) showing the atom-labeling scheme. Displacement ellipsoids are drawn at the 50 % probability level, and H atoms are shown as small spheres of arbitrary radius. | 19 |
| Figure S9 | Molecular structure of (IazCN)_2_(44azpy) showing the atom-labeling scheme. Displacement ellipsoids are drawn at the 50 % probability level, and H atoms are shown as small spheres of arbitrary radius. | 20 |
|  |  |  |
| Figure S10 | Molecular structure of (IazCN)_2_(hpy) showing the atom-labeling scheme. Displacement ellipsoids are drawn at the 50 % probability level, and H atoms are shown as small spheres of arbitrary radius. | 20 |
|  |  |  |
| Figure S11 | Molecular structure of (IazCN)_2_(dpe) showing the atom-labeling scheme. Displacement ellipsoids are drawn at the 50 % probability level, and H atoms are shown as small spheres of arbitrary radius. | 21 |
|  |  |  |
| Figure S12 | PXRD patterns of: a) IazCN calculated pattern from single crystal data, b) IazCN obtained by synthesis. | 22 |
|  |  |  |
| Figure S13 | PXRD patterns of: a) IazCl calculated pattern from single crystal data, b) IazCl obtained by synthesis. | 22 |
|  |  |  |
| Figure S14 | PXRD patterns of: a) IazH calculated pattern from single crystal data, b) IazH obtained by synthesis. | 23 |
|  |  |  |
| Figure S15 | PXRD patterns of: a) IazCN, b) 44azpy, c) product obtained by solution crystallization of 44azpy and IazCN in a 1:2 stoichiometric ratio from acetone d) calculated pattern from single crystal data. | 23 |
|  |  |  |
| Figure S16 | PXRD patterns of: a) IazCN, b) hpy, c) product obtained by solution crystallization of hpy and IazCN in a 1:2 stoichiometric ratio from acetone d) calculated pattern from single crystal data. | 24 |
|  |  |  |
| Figure S17 | PXRD patterns of: a) IazCl, b) 44azpy, c) product obtained by solution crystallization of 44azpy and IazCl in a 1:2 stoichiometric ratio from acetone d) calculated pattern from single crystal data. | 24 |
|  |  |  |
| Figure S18 | PXRD patterns of: a) IazCl, b) hpy, c) product obtained by solution crystallization of hpy and IazCl in a 1:2 stoichiometric ratio from acetone d) calculated pattern from single crystal data. | 25 |
|  |  |  |
| Figure S19 | PXRD patterns of: a) IazCl, b) dpe, c) product obtained by solution crystallization of dpe and IazCl in a 1:2 stoichiometric ratio from nitromethane d) calculated pattern from single crystal data. | 25 |
|  |  |  |
| Figure S20 | PXRD patterns of: a) IazCl, b) dpa, c) product obtained by solution crystallization of dpa and IazCl in a 1:2 stoichiometric ratio from acetonitrile d) calculated pattern from single crystal data. | 26 |
|  |  |  |
| Figure S21 | PXRD patterns of: a) IazCl, b) 44bpy, c) product obtained by solution crystallization of 44bpy and IazCl in a 1:2 stoichiometric ratio from EtOH d) calculated pattern from single crystal data. | 26 |
|  |  |  |
| Figure S22 | PXRD patterns of: a) IazCN, b) 44bpy, c) product obtained by solution crystallization of 44azpy and IazCN in a 1:2 stoichiometric ratio from acetone d) product obtained by solution crystallization of 44azpy and IazCN in a 1:2 stoichiometric ratio from ethanol. | 27 |
|  |  |  |
| Figure S23 | PXRD patterns of: a) IazCN, b) dpa, c) product obtained by solution crystallization of dpa and IazCN in a 1:2 stoichiometric ratio from acetone d) product obtained by solution crystallization of dpa and IazCN in a 1:2 stoichiometric ratio from ethanol. | 27 |
|  |  |  |
| Figure S24 | PXRD patterns of: a) IazCl, b) 44bpy, c) product obtained by grinding 44bpy and IazCl in a 1:2 stoichiometric ratio d) calculated pattern from single crystal data. | 28 |
|  |  |  |
| Figure S25 | PXRD patterns of: a) IazCl, b) 44azpy, c) product obtained by grinding 44azpy and IazCl in a 1:2 stoichiometric ratio d) calculated pattern from single crystal data. | 28 |
|  |  |  |
| Figure S26 | PXRD patterns of: a) IazCl, b) hpy, c) product obtained by grinding hpy and IazCl in a 1:2 stoichiometric ratio d) calculated pattern from single crystal data. | 29 |
|  |  |  |
| Figure S27 | PXRD patterns of: a) IazCl, b) dpe, c) product obtained by grinding dpe and IazCl in a 1:2 stoichiometric ratio d) calculated pattern from single crystal data. | 29 |
|  |  |  |
| Figure S28 | PXRD patterns of: a) IazCl, b) dpa, c) product obtained by grinding dpa and IazCl in a 1:2 stoichiometric ratio d) calculated pattern from single crystal data. | 30 |
|  |  |  |
| Figure S29 | PXRD patterns of: a) IazCN, b) 44bpy, c) product obtained by grinding 44bpy and IazCN in a 1:2 stoichiometric ratio. | 30 |
|  |  |  |
| Figure S30 | PXRD patterns of: a) IazCN, b) 44azpy, c) product obtained by grinding 44azpy and IazCN in a 1:2 stoichiometric ratio d) calculated pattern from single crystal data. | 31 |
|  |  |  |
| Figure S31 | PXRD patterns of: a) IazCN, b) hpy, c) product obtained by grinding hpy and IazCN in a 1:2 stoichiometric ratio d) calculated pattern from single crystal data. | 31 |
|  |  |  |
| Figure S32 | PXRD patterns of: a) IazCN, b) dpe, c) product obtained by grinding dpe and IazCN in a 1:2 stoichiometric ratio d) calculated pattern from single crystal data. | 32 |
|  |  |  |
| Figure S33 | PXRD patterns of: a) IazCN, b) dpa, c) product obtained by grinding dpa and IazCN in a 1:2 stoichiometric ratio. | 32 |
|  |  |  |
| Figure S34 | PXRD patterns of: a) IazH, b) 44bpy, c) product obtained by grinding 44bpy and IazH in a 1:2 stoichiometric ratio. | 33 |
|  |  |  |
| Figure S35 | PXRD patterns of: a) IazH, b) 44azpy, c) product obtained by grinding 44azpy and IazH in a 1:2 stoichiometric ratio. | 33 |
|  |  |  |
| Figure S36 | PXRD patterns of: a) IazH, b) hpy, c) product obtained by grinding hpy and IazH in a 1:2 stoichiometric ratio. | 34 |
|  |  |  |
| Figure S37 | PXRD patterns of: a) IazH, b) dpa, c) product obtained by grinding dpa and IazH in a 1:2 stoichiometric ratio. | 34 |
|  |  |  |
| Figure S38 | PXRD patterns of: a) IazH, b) dpe, c) product obtained by grinding dpe and IazH in a 1:2 stoichiometric ratio. | 35 |
|  |  |  |
| Figure S39 | PXRD patterns of: a) IazH, products obtained by solution crystallization of IazH and b) 44bpy, c) 44azpy, d) hpy, e) dpe, f) dpa in a 2:1 stoichiometric ratio from ethanol. | 35 |
|  |  |  |
| Figure S40 | PXRD patterns of: a) IazH, b) 22bpy, c) product obtained by grinding 22bpy and IazH in a 1:2 stoichiometric ratio. | 36 |
|  |  |  |
| Figure S41 | PXRD patterns of: a) IazCl, b) 22bpy, c) product obtained by grinding 22bpy and IazCl in a 1:2 stoichiometric ratio. | 36 |
|  |  |  |
| Figure S42 | PXRD patterns of: a) IazCN, b) 22bpy, c) product obtained by grinding 22bpy and IazCN in a 1:2 stoichiometric ratio. | 37 |
|  |  |  |
| Figure S43 | PXRD patterns of: a) IazH, b) 22bpy, c) product obtained by solution crystallization of 22bpy and IazH in a 1:2 stoichiometric ratio from ethanol, d) product obtained by solution crystallization of 22bpy and IazH in a 1:2 stoichiometric ratio from acetone. | 37 |
|  |  |  |
| Figure S44 | PXRD patterns of: a) IazCl, b) 22bpy, c) product obtained by solution crystallization of 22bpy and IazCl in a 1:2 stoichiometric ratio from ethanol, d) product obtained by solution crystallization of 22bpy and IazCl in a 1:2 stoichiometric ratio from acetone. | 38 |
|  |  |  |
| Figure S45 | PXRD patterns of: a) IazCN, b) 22bpy, c) product obtained by solution crystallization of 22bpy and IazCN in a 1:2 stoichiometric ratio from ethanol, d) product obtained by solution crystallization of 22bpy and IazCN in a 1:2 stoichiometric ratio from acetone. | 38 |
|  |  |  |
| Figure S46 | PXRD patterns of: a) IazH, b) dmap, c) product obtained by solution crystallization of dmap and IazH in a 1:1 stoichiometric ratio from ethanol, d) product obtained by solution crystallization of dmap and IazH in a 1:1 stoichiometric ratio from acetone. | 39 |
|  |  |  |
| Figure S47 | PXRD patterns of: a) IazCl, b) dmap, c) product obtained by solution crystallization of dmap and IazCl in a 1:1 stoichiometric ratio from ethanol, d) product obtained by solution crystallization of dmap and IazCl in a 1:1 stoichiometric ratio from acetone. | 39 |
|  |  |  |
| Figure S48 | PXRD patterns of: a) IazCN, b) dmap, c) product obtained by solution crystallization of dmap and IazCN in a 1:1 stoichiometric ratio from ethanol, d) product obtained by solution crystallization of dmap and IazCN in a 1:1 stoichiometric ratio from acetone. | 40 |
|  |  |  |
| Figure S49 | PXRD patterns of: a) IazH, b) dabco, c) product obtained by solution crystallization of dabco and IazH in a 1:2 stoichiometric ratio from ethanol, d) product obtained by solution crystallization of dabco and IazH in a 1:2 stoichiometric ratio from acetone. | 40 |
|  |  |  |
| Figure S50 | PXRD patterns of: a) IazCl, b) dabco, c) product obtained by solution crystallization of dabco and IazCl in a 1:2 stoichiometric ratio from ethanol, d) product obtained by solution crystallization of dabco and IazCl in a 1:2 stoichiometric ratio from acetone. | 41 |
|  |  |  |
| Figure S51 | PXRD patterns of: a) IazCN, b) dabco, c) product obtained by solution crystallization of dabco and IazCN in a 1:2 stoichiometric ratio from ethanol, d) product obtained by solution crystallization of dabco and IazCN in a 1:2 stoichiometric ratio from acetone. | 41 |
|  |  |  |
| Figure S52 | DSC curve of IazH. | 42 |
|  |  |  |
| Figure S53 | DSC curve of IazCN. | 42 |
|  |  |  |
| Figure S54 | DSC curve of IazCl. | 43 |
|  |  |  |
| Figure S55 | DSC curve of (IazCl)(44bpy). | 43 |
|  |  |  |
| Figure S56 | DSC curve of (IazCl)_2_(44azpy). | 44 |
|  |  |  |
| Figure S57 | DSC curve of (IazCl)_2_(dpe). | 44 |
|  |  |  |
| Figure S58 | DSC curve of (IazCl)_2_(hpy). | 45 |
|  |  |  |
| Figure S59 | DSC curve of (IazCl)_2_(dpa). | 45 |
|  |  |  |
| Figure S60 | DSC curve of (IazCN)_2_(44azpy). | 46 |
|  |  |  |
| Figure S61 | DSC curve of (IazCN)_2_(dpe). | 46 |
|  |  |  |
| Figure S62 | DSC curve of (IazCN)_2_(hpy). | 47 |
|  |  |  |
| Figure S63 | DSC curve of 44bpy | 47 |
|  |  |  |
| Figure S64 | DSC curve of 44azpy | 48 |
|  |  |  |
| Figure S65 | DSC curve of hpy | 48 |
|  |  |  |
| Figure S66 | DSC curve of dpa | 49 |
|  |  |  |
| Figure S67 | DSC curve of dpe | 49 |
|  |  |  |
| Table S4 | Percentages for individual intermolecular interactions of IazCl and IazCN molecules in cocrystals with five bipyridyl acceptors | 50 |
|  |  |  |
| Table S5 | Halogen bond, halogen contact and hydrogen bond lengths, angles and distances in the prepared cocrystals. | 51 |
|  |  |  |
| Computational details | Molecular electrostatic potential map of 44azpy, 44bpy, dpa, dpe, hpy, dmap, dabco and 22bpy. | 52؎53 |

**Experimental details**

**Mechanochemical experiments**

**Table S1.** Experimental data for mechanochemical experiments. Reaction mixtures were placed in 10 mL stainless steel jars along with 10 μL of acetonitrile and one stainless steel ball 7 mm in diameter. The reaction mixtures were then milled in a Retsch MM200 Shaker Mill operating at 25 Hz

| acceptor | *m* / mg | donor | *m* / mg | *t* / min | result |
| --- | --- | --- | --- | --- | --- |
| 44bpy | 8.1 | **IazH** | 51.9 | 20 | amorphous phase |
| 44azpy | 9.4 | **IazH** | 50.6 | 20 | amorphous phase |
| hpy | 10.5 | **IazH** | 49.5 | 20 | amorphous phase |
| dpe | 9.3 | **IazH** | 50.7 | 20 | amorphous phase |
| dpa | 9.4 | **IazH** | 50.6 | 20 | amorphous phase |
| 22bpy | 8.1 | **IazH** | 51.5 | 20 | amorphous phase |
| dmap | 12.4 | **IazH** | 47.6 | 20 | oil |
| dabco | 6.4 | **IazH** | 53.6 | 20 | oil |
| 44bpy | 8.0 | **IazCl** | 52.0 | 20 | amorphous phase |
| 44azpy | 9.2 | **IazCl** | 50.8 | 20 | amorphous phase |
| hpy | 10.3 | **IazCl** | 49.7 | 20 | amorphous phase |
| dpe | 9.1 | **IazCl** | 50.9 | 20 | amorphous phase |
| dpa | 9.2 | **IazCl** | 50.8 | 20 | amorphous phase |
| 22bpy | 8.0 | **IazCl** | 52.0 | 20 | amorphous phase |
| dmap | 6.2 | **IazCl** | 53.8 | 20 | oil |
| dabco | 6.0 | **IazCl** | 54.0 | 20 | oil |
| 44bpy | 8.1 | **IazCN** | 51.9 | 20 | amorphous phase |
| 44azpy | 9.4 | **IazCN** | 50.6 | 20 | amorphous phase |
| hpy | 10.5 | **IazCN** | 49.5 | 20 | amorphous phase |
| dpe | 9.3 | **IazCN** | 50.7 | 20 | amorphous phase |
| dpa | 9.4 | **IazCN** | 50.6 | 20 | amorphous phase |
| 22bpy | 8.1 | **IazCN** | 51.9 | 20 | amorphous phase |
| dmap | 11.8 | **IazCN** | 58.2 | 20 | oil |
| dabco | 6.1 | **IazCN** | 53.9 | 20 | oil |

**Solution syntheses**

**Table S2.** Experimental data for solution experiments. Reaction mixtures were carefully heated to dissolve the reactants, if necessary, and then left to crystallize at room temperature.

| reaction mixture | stoichiometric ratio | solvent | result |
| --- | --- | --- | --- |
| 30.0 mg IazH | / | acetone | **IazH**  single crystals |
| 30.0 mg IazCl | / | nitromethane | **IazH**  single crystals |
| 30.0 mg IazCN | / | nitromethane | **IazH**  single crystals |
| 22.9 mg IazCl  7.1 mg 44bpy | 1:1 | 2.0 mL ethanol | (**IazCl**)(**44bpy**)  single crystals |
| 25.4 mg IazCl 4.6 mg 44azpy | 2:1 | 2.0 mL acetone | (**IazCl**)_2_(**44azpy**)  single crystals |
| 25.4 mg IazCl 4.6 mg dpe | 2:1 | 2.0 mL nitromethane | (**IazCl**)_2_(**dpe**)  single crystals |
| 25.4 mg IazCl 4.6 mg dpa | 2:1 | 1. mL acetonitrile   + 1.0 mL acetone | (**IazCl**)_2_(**dpa**)  single crystals |
| 24.8 mg IazCl 5.2 mg hpy | 2:1 | 2.0 mL acetone | (**IazCl**)_2_(**hpy**)  single crystals |
| 26.0 mg IazCl  4.0 mg 22bpy | 2:1 | 2.0 mL ethanol | amorphous phase |
| 26.0 mg IazCl  4.0 mg 22bpy | 2:1 | 2.0 mL acetone | amorphous phase |
| 24.2 mg IazCl  5.8 mg dmap | 1:1 | 2.0 mL ethanol | amorphous phase |
| 26.9 mg IazCl  3.1 mg dmap | 1:1 | 2.0 mL acetone | poorly crystalline |
| 27.0 mg IazCl  3.0 mg dabco | 2:1 | 2.0 mL ethanol | amorphous phase |
| 27.0 mg IazCl  3.0 mg dabco | 2:1 | 2.0 mL acetone | amorphous phase |
| 25.3 mg IazCN 4.7 mg 44azpy | 2:1 | 2.0 mL acetone | (**IazCN**)_2_(**44azpy**)  single crystals |
| 25.3 mg IazCN 4.7 mg dpe | 2:1 | 2.0 mL acetone | (**IazCN**)_2_(**dpe**)  single crystals |
| 24.8 mg IazCN 5.2 mg hpy | 2:1 | 2.0 mL acetone | (**IazCN**)_2_(**hpy**)  single crystals |
| 25.9 mg IazCN 4.1 mg 44bpy | 2:1 | 2.0 mL acetone | poorly crystalline; reactant mixture |
| 25.9 mg IazCN 4.1 mg 44bpy | 2:1 | 2.0 mL ethanol | poorly crystalline; reactant mixture |

**Table S2.** continued

| reaction mixture | stoichiometric ratio | solvent | result |
| --- | --- | --- | --- |
| 25.3 mg IazCN 4.7 mg dpa | 2:1 | 2.0 mL acetone | poorly crystalline; reactant mixture |
| 25.3 mg IazCN 4.7 mg dpa | 2:1 | 2.0 mL ethanol | poorly crystalline; reactant mixture |
| 25.9 mg IazCN 4.1 mg 22bpy | 2:1 | 2.0 mL ethanol | amorphous phase |
| 25.9 mg IazCN 4.1 mg 22bpy | 2:1 | 2.0 mL acetone | poorly crystalline |
| 24.1 mg IazCN 5.9 mg dmap | 1:1 | 2.0 mL ethanol | poorly crystalline |
| 24.1 mg IazCN 5.9 mg dmap | 1:1 | 2.0 mL acetone | poorly crystalline |
| 26.9 mg IazCN 3.1 mg dabco | 2:1 | 2.0 mL ethanol | amorphous phase |
| 26.9 mg IazCN 3.1 mg dabco | 2:1 | 2.0 mL acetone | amorphous phase |
| 25.7 mg IazH  4.3 mg 44bpy | 2:1 | 2.0 mL acetone | oil/glassy residue |
| 25.7 mg IazH  4.3 mg 44bpy | 2:1 | 2.0 mL ethanol | amorphous phase |
| 25.1 mg IazH  4.9 mg 44azpy | 2:1 | 2.0 mL acetone | oil/glassy residue |
| 25.1 mg IazH  4.9 mg 44azpy | 2:1 | 2.0 mL ethanol | amorphous phase |
| 25.1 mg IazH  4.9 mg dpe | 2:1 | 2.0 mL acetone | oil/glassy residue |
| 25.1mg IazH  4.9 mg dpe | 2:1 | 2.0 mL ethanol | amorphous phase |
| 25.1 mg IazH  4.9 mg dpa | 2:1 | 2.0 mL acetone | oil/glassy residue |
| 25.1 mg IazH  4.9 mg dpa | 2:1 | 2.0 mL ethanol | amorphous phase |
| 24.5 mg IazH  5.5 mg hpy | 2:1 | 2.0 mL acetone | oil/glassy residue |
| 24.5 mg IazH  5.5 mg hpy | 2:1 | 2.0 mL ethanol | amorphous phase |
| 25.7 mg IazH  4.3 mg 22bpy | 2:1 | 2.0 mL ethanol | amorphous phase |
| 25.7 mg IazH  4.3 mg 22bpy | 2:1 | 2.0 mL acetone | amorphous phase |

**Table S2.** continued

| reaction mixture | stoichiometric ratio | solvent | result |
| --- | --- | --- | --- |
| 26.6 mg IazH  3.4 mg dmap | 1:1 | 2.0 mL ethanol | amorphous phase |
| 23.8 mg IazH  6.2 mg dmap | 1:1 | 2.0 mL acetone | amorphous phase |
| 23.8 mg IazH  6.2 mg dabco | 2:1 | 2.0 mL ethanol | amorphous phase |
| 26.8 mg IazH  3.2 mg dabco | 2:1 | 2.0 mL acetone | amorphous phase |

**Thermal analysis**

DSC measurements were performed on a Mettler-Toledo DSC823^e^ module. The samples were placed in sealed aluminium pans (40 μL) with two pinholes made on the top cover, and heated in flowing nitrogen (50 mL min^−1^) from 25 °C to 500 °C at a rate of 10 °C min^−1^. The data collection and analysis was performed using the program package STAR^e^ Software 15.00.^1^

**Powder X-ray diffraction experiments**

PXRD experiments were performed on a Malvern PANalytical Aeris X-ray diffractometer with Cu*K*α1 (1.54056 Å) radiation at 15 mA and 40 kV. The scattered intensities were measured with a scintillation counter. The angular range was from 5 to 40° (2*θ* ) with steps of 0.02 – 0.03°, and the measuring time was 0.2 – 0.5 s per step. Data collection and analysis was performed using the program package Data Viewer.^2^

**Single-crystal X-ray diffraction experiments**

The crystal and molecular structures of the prepared cocrystals were determined by single crystal X-ray diffraction. Details of data collection and crystal structure refinement are listed in Table S1, S2, S3 and S4. Diffraction measurements were made on an Oxford Diffraction Xcalibur Kappa CCD X-ray diffractometer and Rigaku Synergy XtaLAB X-ray diffractometer with graphite-monochromated MoKα (*λ* = 0.71073Å) radiation. The data sets were collected using the ω scan mode over the 2*θ* range up to 54° (Xcalibur Kappa CCD) and up to 64° (Synergy XtaLAB). Programs CrysAlis CCD, CrysAlis RED and CrysAlisPro were employed for data collection, cell refinement, and data reduction.^3,4^ The structures were solved by direct methods and refined using the SHELXS, SHELXT, and SHELXL programs, respectively.^5,6^ The structural refinement was performed on *F*^2^ using all data. Hydrogen atoms were placed in calculated positions and treated as riding on their parent atoms. All calculations were performed using the WINGX crystallographic suite of programs.^7^ The molecular structures of compounds and their molecular packing projections were prepared by Mercury.^8^

**References**

1. STARe Evaluation Software Version 15.00, Mettler–Toledo GmbH, 2016.

2. Data Viewer Version 1.9a, PANalytical B.V. Amelo, The Netherlands, 2018.

3. Oxford Diffraction, Oxford Diffraction Ltd., Xcalibur CCD system, CrysAlis CCD and CrysAlis RED software, Version 1.170, 2003.

4. Rigaku Oxford Diffraction, Gemini CCD system, CrysAlis Pro software, Version 171.41.93a, 2020.

5. (a) G. M. Sheldrick, *Acta Cryst. A*, 2008, **64**, 112–122; (b) G. M. Sheldrick, *Acta Cryst. C*, 2015, **71**, 3–8.

6. G. M. Sheldrick, *Acta Cryst. A*, 2015, **71**, 3–8.

7. L. J. Farrugia, *J. Appl. Cryst.*, 2012, **45**, 849–854.

8. C. F. Macrae, I. J. Bruno, J. A. Chisholm, P. R. Edgington, P. McCabe, E. Pidcock, L. Rodriguez-Monge, R. Taylor, J. v. d. Streek and P. A. Wood, *J. Appl. Crystallogr.* **2008**, 41, 466.

**Table S3.** Crystal data and refinement details for the prepared compounds.

|  | **IazH** | **IazCl** |
| --- | --- | --- |
| Molecular formula | C_18_H_9_F_4_IN_2_O | C_18_H_8_ClF_4_IN_2_O |
| CCDC number | 2386716 | 2386719 |
| *M*_r_ | 472.17 | 506.61 |
| Crystal system | orthorhombic | monoclinic |
| Space group | *P* 2_1_2_1_2_1_ | *P* 2_1_/c |
| Crystal data: |  |  |
| *a* / Å | 5.9657(4) | 8.0319(2) |
| *b* / Å | 7.6733(7) | 5.83980(10) |
| *c* / Å | 36.872(2) | 36.9927(9) |
| *α* / ° | 90 | 90 |
| *β* / ° | 90 | 94.049(2) |
| *γ* / ° | 90 | 90 |
| *V* / Å^3^ | 1687.9(2) | 1730.80(7) |
| *Z* | 4 | 4 |
| *D*_calc_ / g cm^−3^ | 1.858 | 1.832 |
| *λ*(Mo*K*_α_) / Å | 0.71073 | 0.71073 |
| *T* / K | 295 | 169.99(10) |
| Crystal size / mm^3^ | 0.52 x 0.17 x 0.07 | 0.52 x 0.27 x 0.05 |
| *μ* / mm^−1^ | 1.948 | 2.056 |
| *F*(000) | 912 | 976 |
| Refl. collected/unique | 16912 / 4931 | 41624 / 19680 |
| Parameters/restraints | 235 / 0 | 244 / 0 |
| Δ*ρ*_max_ , Δ*ρ*_min_ / e Å^−3^ | 0.534; -0.593 | 2.648; -0.845 |
| *R*[*F*^2^ > 4*σ*(*F*^2^)] | 0.0459 | 0.0513 |
| w*R*(*F*^2^) | 0.1042 | 0.1256 |
| Goodness-of-fit, *S* | 1.035 | 1.207 |

**Table S3.** Crystal data and refinement details for the prepared compounds. (continued)

|  | **IazCN** | (**IazCl**)(**44bpy**) |
| --- | --- | --- |
| Molecular formula | C_19_H_8_F_4_IN_3_O | (C_18_H_8_ClF_4_IN_2_O)(C_10_H_8_N_2_) |
| CCDC number | 2386722 | 2386718 |
| *M*_r_ | 497.18 | 662.80 |
| Crystal system | monoclinic | triclinic |
| Space group | *C* 2/c | *P* -1 |
| Crystal data: |  |  |
| *a* / Å | 22.0482(3) | 7.4889(2) |
| *b* / Å | 5.70770(10) | 13.3949(3) |
| *c* / Å | 29.0370(4) | 13.8064(4) |
| *α* / ° | 90 | 79.366(2) |
| *β* / ° | 98.6890(10) | 77.913(2) |
| *γ* / ° | 90 | 74.125(2) |
| *V* / Å^3^ | 3612.21(9) | 1290.63(6) |
| *Z* | 8 | 2 |
| *D*_calc_ / g cm^−3^ | 1.828 | 1.706 |
| *λ*(Mo*K*_α_) / Å | 0.71073 | 0.71073 |
| *T* / K | 295 | 295 |
| Crystal size / mm^3^ | 0.41 x 0.30 x 0.13 | 0.87 x 0.46 x 0.07 |
| *μ* / mm^−1^ | 1.827 | 1.403 |
| *F*(000) | 1920 | 652 |
| Refl. collected/unique | 134885 / 6266 | 81409 / 8775 |
| Parameters/restraints | 253 / 0 | 352 / 0 |
| Δ*ρ*_max_ , Δ*ρ*_min_ / e Å^−3^ | 1.247; -0.552 | 1.124; -0.641 |
| *R*[*F*^2^ > 4*σ*(*F*^2^)] | 0.0487 | 0.0513 |
| w*R*(*F*^2^) | 0.1301 | 0.1256 |
| Goodness-of-fit, *S* | 1.059 | 1.076 |

**Table S3.** Crystal data and refinement details for the prepared compounds. (continued)

|  | (**IazCl)**_2_(**44azpy**) | (**IazCl**)_2_(**dpe**) |
| --- | --- | --- |
| Molecular formula | (C_18_ H_8_ Cl F_4_ I N_2_ O)_2_ (C_10_ H_8_ N_4_) | (C_18_ H_8_ C_l_ F_4_ I N_2_ O)_2_ (C_12_ H_10_ N_2_) |
| CCDC number | 2386723 | 2386713 |
| *M*_r_ | 1197.43 | 1195.45 |
| Crystal system | monoclinic | triclinic |
| Space group | *P* 2_1_/n | *P* -1 |
| Crystal data: |  |  |
| *a* / Å | 26.8575(4) | 5.7689(2) |
| *b* / Å | 5.89280(10) | 13.3191(5) |
| *c* / Å | 28.4972(6) | 16.2168(5) |
| *α* / ° | 90 | 70.925(3) |
| *β* / ° | 92.629(2) | 85.940(3) |
| *γ* / ° | 90 | 79.577(3) |
| *V* / Å^3^ | 4505.39(14) | 1158.10(7) |
| *Z* | 4 | 1 |
| *D*_calc_ / g cm^−3^ | 1.765 | 1.714 |
| *λ*(Mo*K*_α_) / Å | 0.71073 | 0.71073 |
| *T* / K | 295 | 295 |
| Crystal size / mm^3^ | 0.55 x 0.18 x 0.11 | 0.70 x 0.18 x 0.03 |
| *μ* / mm^−1^ | 1.598 | 1.552 |
| *F*(000) | 2336 | 584 |
| Refl. collected/unique | 102140 / 9823 | 15467/ 8592 |
| Parameters/restraints | 613 / 0 | 307 / 0 |
| Δ*ρ*_max_ , Δ*ρ*_min_ / e Å^−3^ | 0.715; -0.591 | 1.212; -0.810 |
| *R*[*F*^2^ > 4*σ*(*F*^2^)] | 0.0632 | 0.0563 |
| w*R*(*F*^2^) | 0.1013 | 0.1541 |
| Goodness-of-fit, *S* | 1.042 | 1.024 |

**Table S3.** Crystal data and refinement details for the prepared compounds. (continued)

|  | (**IazCl**)_2_(**dpa**) | (**IazCl**)_2_(**hpy**) |
| --- | --- | --- |
| Molecular formula | (C_18_ H_8_ Cl F_4_ I N_2_ O)_2_ (C_12_ H_12_ N_2_) | (C_18_ H_8_ Cl F_4_ I N_2_ O)_2_ (C_12_ H_10_ N_4_) |
| CCDC number | 2386717 | 2386714 |
| *M*_r_ | 1197.46 | 1223.47 |
| Crystal system | monoclinic | monoclinic |
| Space group | *P* 2_1_/c | *P* 2_1_/c |
| Crystal data: |  |  |
| *a* / Å | 5.5638(4) | 12.9037(7) |
| *b* / Å | 8.6682(4) | 5.8897(3) |
| *c* / Å | 48.157(3) | 31.1507(17) |
| *α* / ° | 90 | 90 |
| *β* / ° | 90.121(5) | 93.806(5) |
| *γ* / ° | 90 | 90 |
| *V* / Å^3^ | 2322.5(2) | 2362.2(2) |
| *Z* | 2 | 2 |
| *D*_calc_ / g cm^−3^ | 1.712 | 1.720 |
| *λ*(Mo*K*_α_) / Å | 0.71073 | 0.71073 |
| *T* / K | 295 | 295 |
| Crystal size / mm^3^ | 0.70 x 0.37 x 0.18 | 0.82 x 0.20 x 0.09 |
| *μ* / mm^−1^ | 1.548 | 1.526 |
| *F*(000) | 1172 | 1196 |
| Refl. collected/unique | 16077 / 6094 | 21181/ 5140 |
| Parameters/restraints | 307/ 0 | 316/ 0 |
| Δ*ρ*_max_ , Δ*ρ*_min_ / e Å^−3^ | 0.991; -1.055 | 0.527; -0.759 |
| *R*[*F*^2^ > 4*σ*(*F*^2^)] | 0.0789 | 0.0424 |
| w*R*(*F*^2^) | 0.2119 | 0.0958 |
| Goodness-of-fit, *S* | 1.031 | 1.061 |

**Table S3.** Crystal data and refinement details for the prepared compounds. (continued)

|  | (**IazCN**)_2_(**44azpy**) | (**IazCN**)_2_(**dpe**) |
| --- | --- | --- |
| Molecular formula | (C_19_ H_8_ F_4_ I N_3_ O)_2_ C_10_ H_8_ N_4_ | (C_19_ H_8_ F_4_ I N_3_ O)_2_ (C_12_ H_10_ N_2_) |
| CCDC number | 2386721 | 2386720 |
| *M*_r_ | 1178.57 | 1176.59 |
| Crystal system | monoclinic | monoclinic |
| Space group | *P* 2_1_/c | *C* 2/c |
| Crystal data: |  |  |
| *a* / Å | 13.0285(6) | 22.0765(9) |
| *b* / Å | 5.9342(3) | 5.5677(2) |
| *c* / Å | 28.9849(14) | 37.3204(15) |
| *α* / ° | 90 | 90 |
| *β* / ° | 92.399(4) | 97.951(4) |
| *γ* / ° | 90 | 90 |
| *V* / Å^3^ | 2238.97(19) | 4543.2(3) |
| *Z* | 2 | 4 |
| *D*_calc_ / g cm^−3^ | 1.748 | 1.720 |
| *λ*(Mo*K*_α_) / Å | 0.71073 | 0.71073 |
| *T* / K | 295 | 295 |
| Crystal size / mm^3^ | 0.35 x 0.18 x 0.06 | 0.85 x 0.15 x 0.05 |
| *μ* / mm^−1^ | 1.492 | 1.469 |
| *F*(000) | 1152 | 2304 |
| Refl. collected/unique | 24786 / 6338 | 20691 / 4951 |
| Parameters/restraints | 316/ 0 | 316/ 0 |
| Δ*ρ*_max_ , Δ*ρ*_min_ / e Å^−3^ | 0.789; -0.560 | 0.642; -0.358 |
| *R*[*F*^2^ > 4*σ*(*F*^2^)] | 0.0448 | 0.0329 |
| w*R*(*F*^2^) | 0.1113 | 0.0889 |
| Goodness-of-fit, *S* | 1.028 | 1.028 |

**Table S3.** Crystal data and refinement details for the prepared compounds. (continued)

|  | (**IazCN**)_2_(**hpy**) |
| --- | --- |
| Molecular formula | (C_19_ H_8_ F_4_ I N_3_ O)_2_ (C_12_ H_10_ N_4_) |
| CCDC number | 2386715 |
| *M*_r_ | 1204.61 |
| Crystal system | monoclinic |
| Space group | *P* 2_1_/c |
| Crystal data: |  |
| *a* / Å | 12.8051(5) |
| *b* / Å | 5.8965(3) |
| *c* / Å | 31.1864(15) |
| *α* / ° | 90 |
| *β* / ° | 93.537(4) |
| *γ* / ° | 90 |
| *V* / Å^3^ | 2350.25(19) |
| *Z* | 2 |
| *D*_calc_ / g cm^−3^ | 1.702 |
| *λ*(Mo*K*_α_) / Å | 0.71073 |
| *T* / K | 295 |
| Crystal size / mm^3^ | 0.71 x 0.17 x 0.08 |
| *μ* / mm^−1^ | 1.423 |
| *F*(000) | 1180 |
| Refl. collected/unique | 27600/ 9919 |
| Parameters/restraints | 325/ 0 |
| Δ*ρ*_max_ , Δ*ρ*_min_ / e Å^−3^ | 0.761; -0.708 |
| *R*[*F*^2^ > 4*σ*(*F*^2^)] | 0.0376 |
| w*R*(*F*^2^) | 0.1009 |
| Goodness-of-fit, *S* | 1.055 |

**
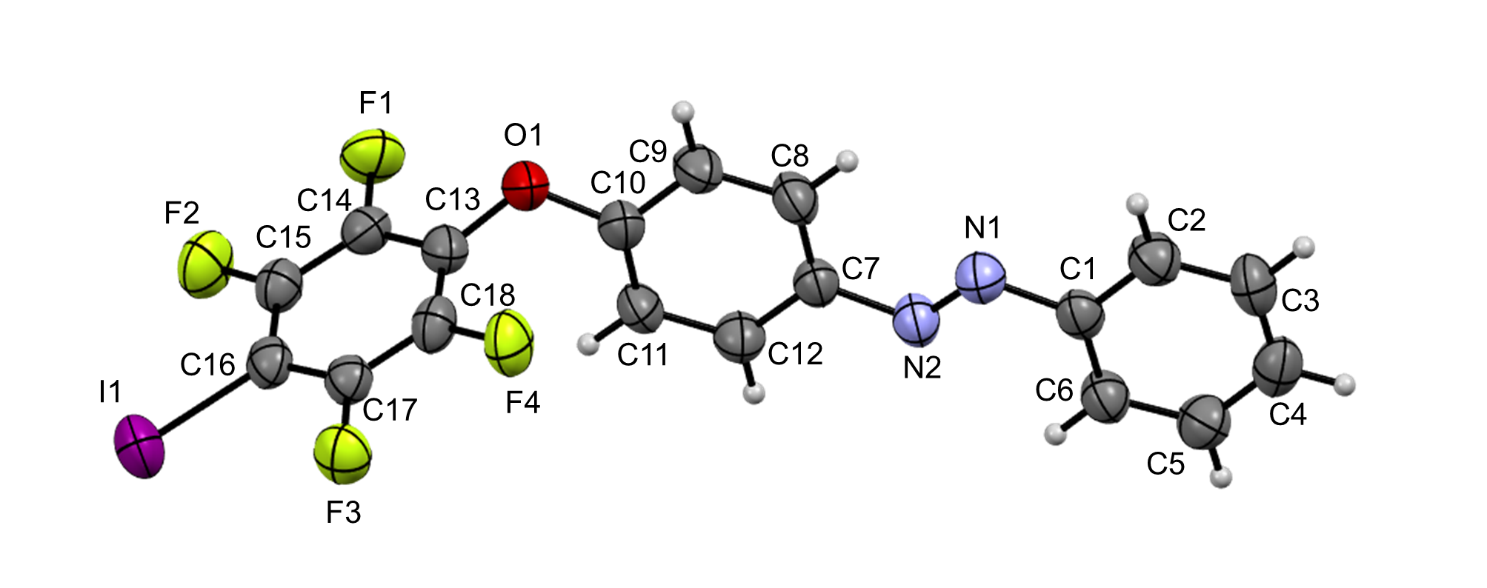
**

**Figure S1.** Molecular structure of **IazH** showing the atom-labeling scheme. Displacement ellipsoids are drawn at the 50 % probability level, and H atoms are shown as small spheres of arbitrary radius.


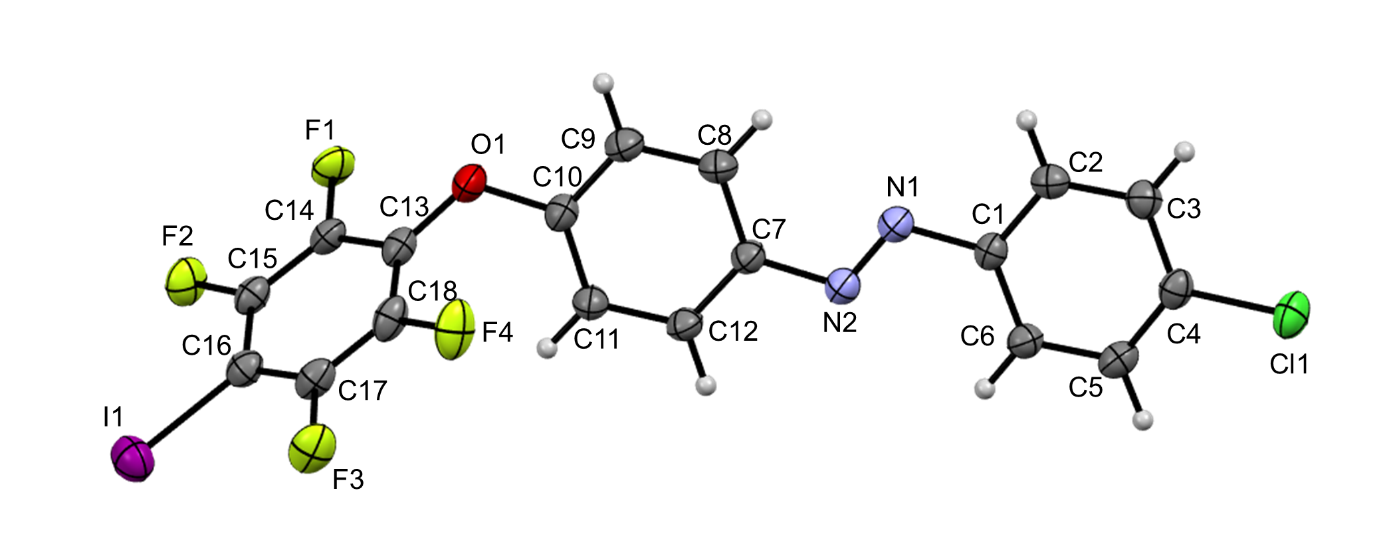


**Figure S2.** Molecular structure of **IazCl** showing the atom-labeling scheme. Displacement ellipsoids are drawn at the 50 % probability level, and H atoms are shown as small spheres of arbitrary radius.


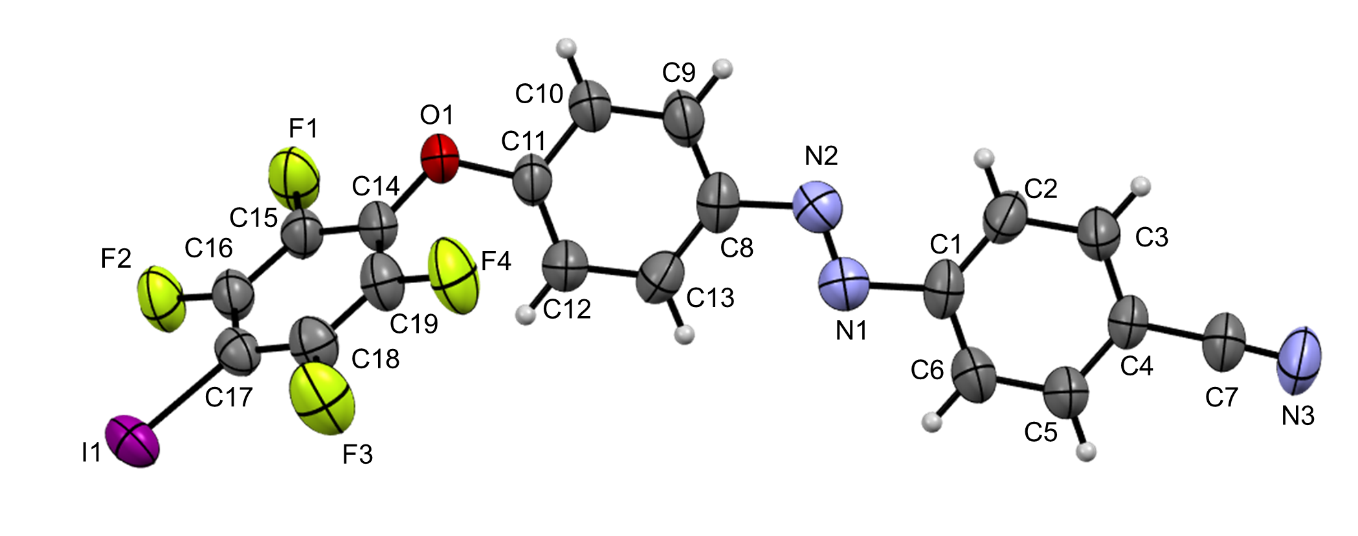


**Figure S3.** Molecular structure of **IazCN** showing the atom-labeling scheme. Displacement ellipsoids are drawn at the 50 % probability level, and H atoms are shown as small spheres of arbitrary radius.


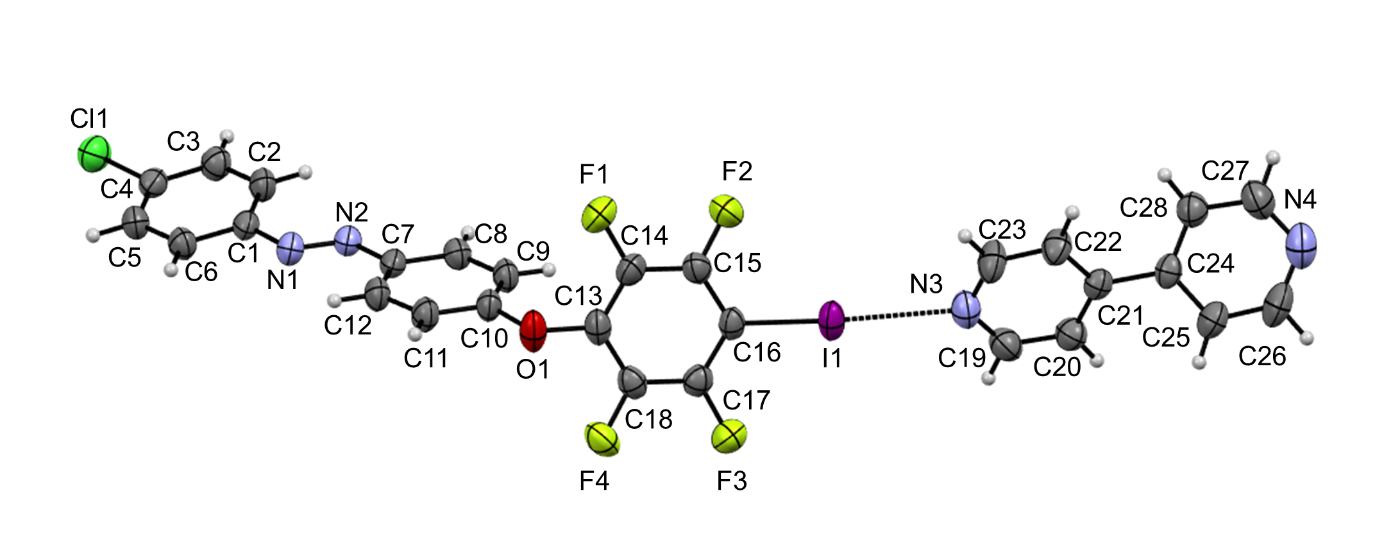


**Figure S4.** Molecular structure of (**IazCl**)(**44bpy**) showing the atom-labeling scheme. Displacement ellipsoids are drawn at the 50 % probability level, and H atoms are shown as small spheres of arbitrary radius.


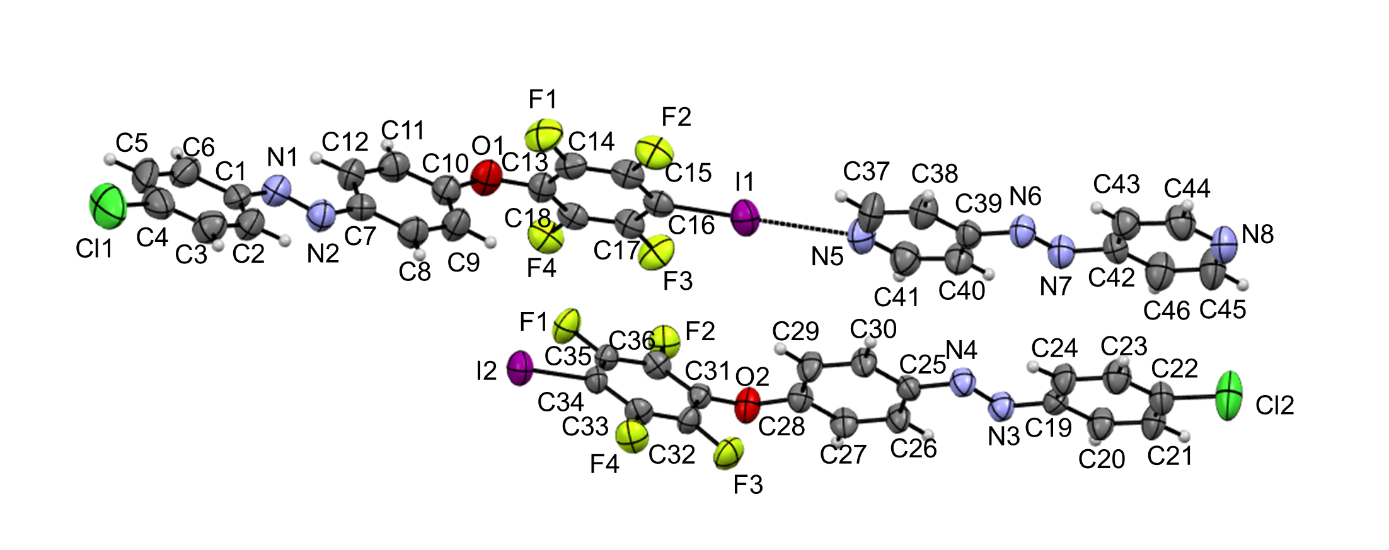


**Figure S5.** Molecular structure of (**IazCl**)_2_(**44azpy**) showing the atom-labeling scheme. Displacement ellipsoids are drawn at the 50 % probability level, and H atoms are shown as small spheres of arbitrary radius.


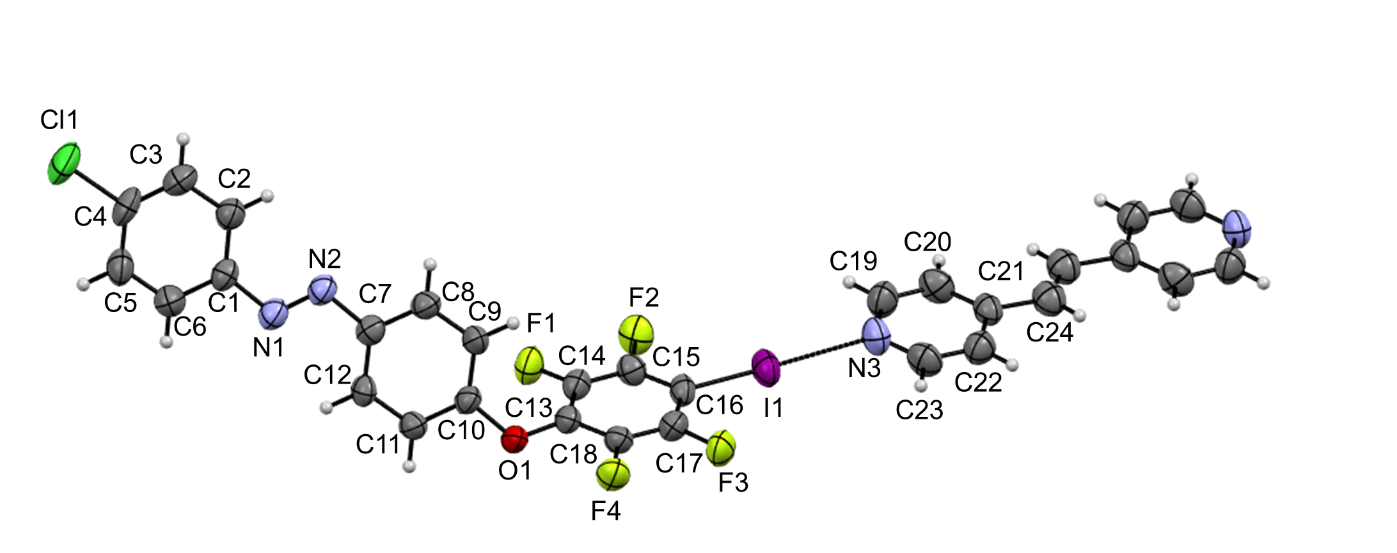


**Figure S6.** Molecular structure of (**IazCl**)_2_(**dpe**) showing the atom-labeling scheme. Displacement ellipsoids are drawn at the 50 % probability level, and H atoms are shown as small spheres of arbitrary radius.


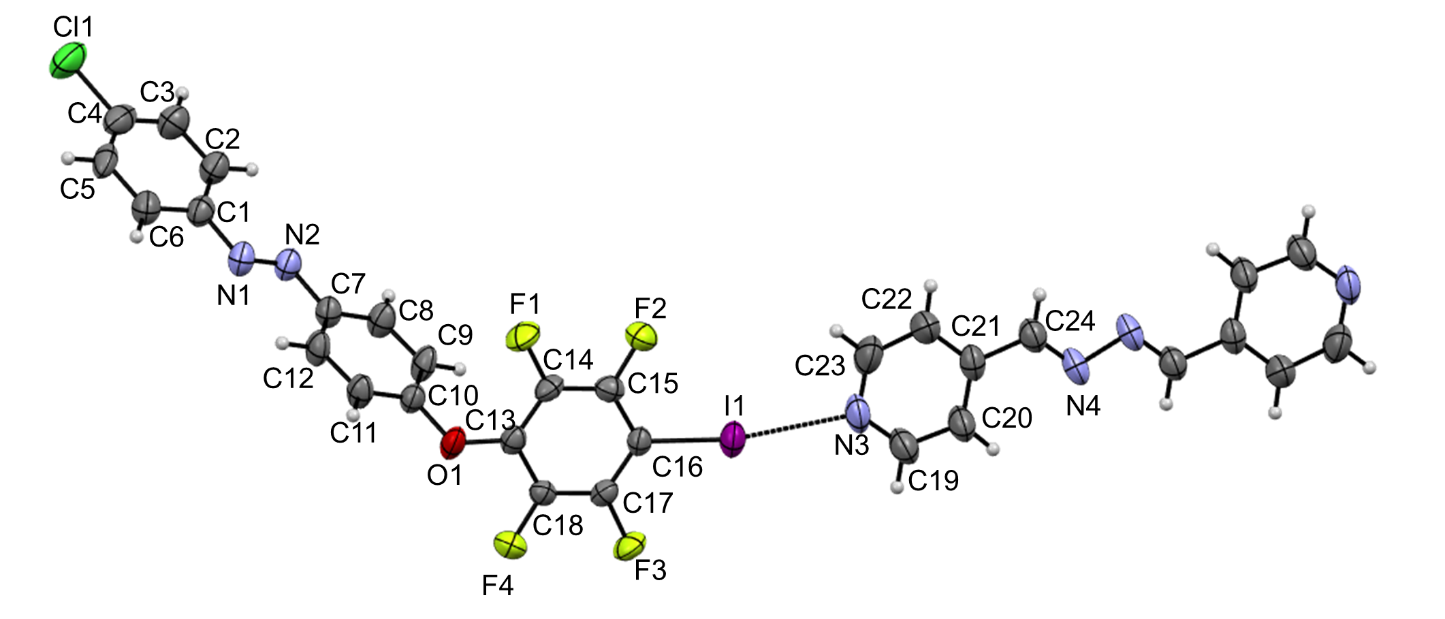


**Figure S7.** Molecular structure of (**IazCl**)_2_(**hpy**) showing the atom-labeling scheme. Displacement ellipsoids are drawn at the 50 % probability level, and H atoms are shown as small spheres of arbitrary radius.


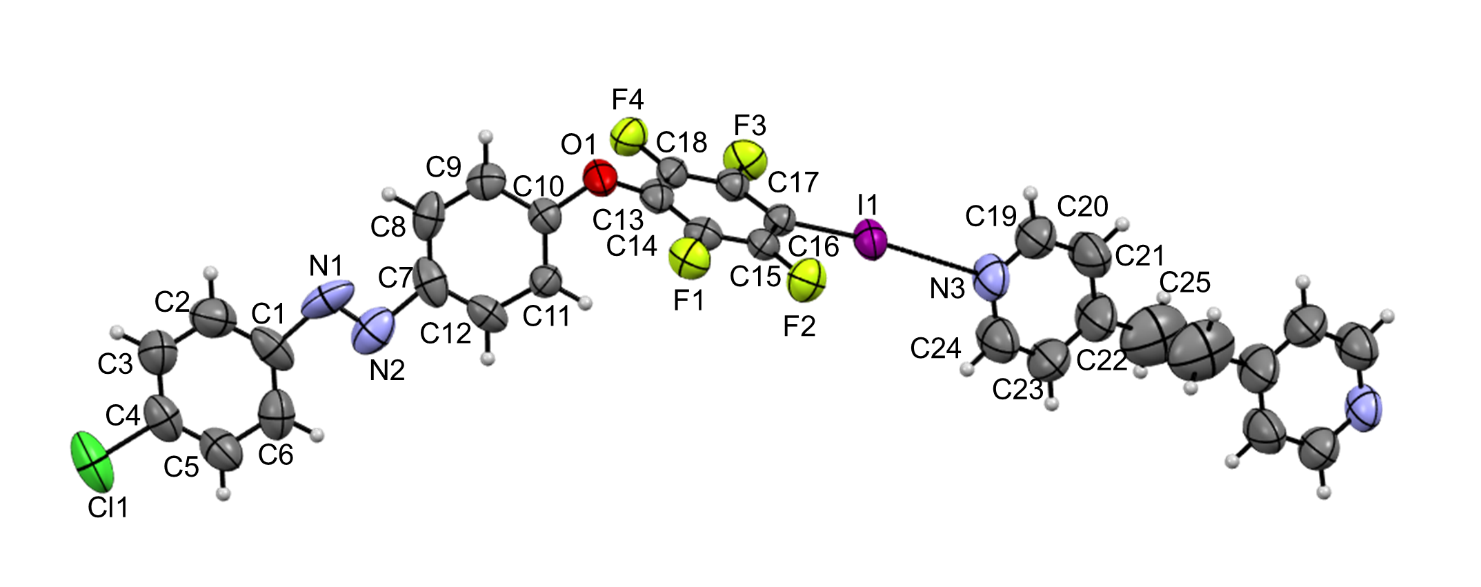


**Figure S8.** Molecular structure of (**IazCl**)_2_(**dpa**) showing the atom-labeling scheme. Displacement ellipsoids are drawn at the 50 % probability level, and H atoms are shown as small spheres of arbitrary radius.


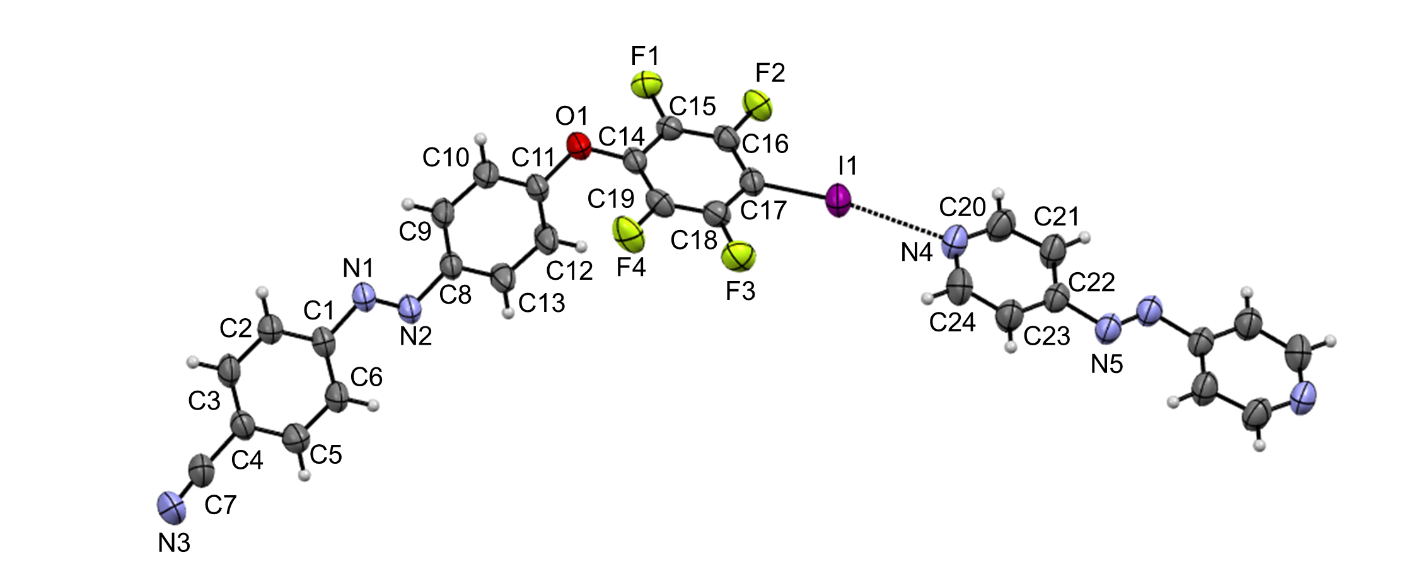


**Figure S9.** Molecular structure of (**IazCN**)_2_(**44azpy**) showing the atom-labeling scheme. Displacement ellipsoids are drawn at the 50 % probability level, and H atoms are shown as small spheres of arbitrary radius.


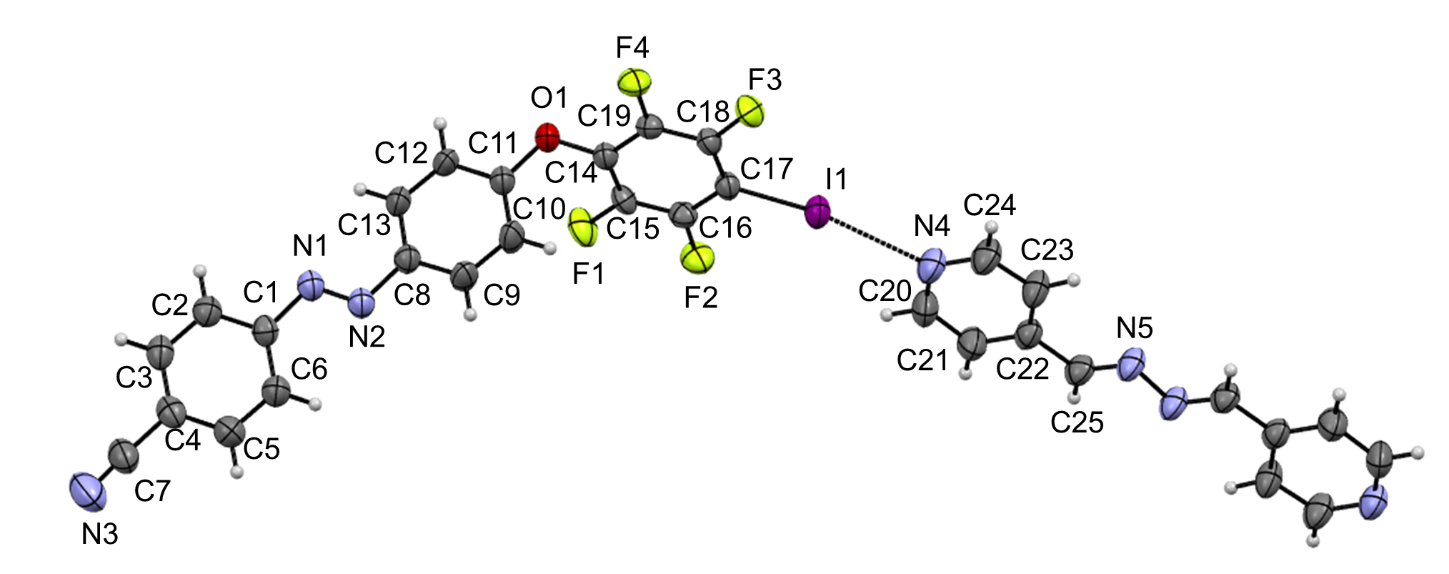


**Figure S10.** Molecular structure of (**IazCN**)_2_(**hpy**) showing the atom-labeling scheme. Displacement ellipsoids are drawn at the 50 % probability level, and H atoms are shown as small spheres of arbitrary radius.


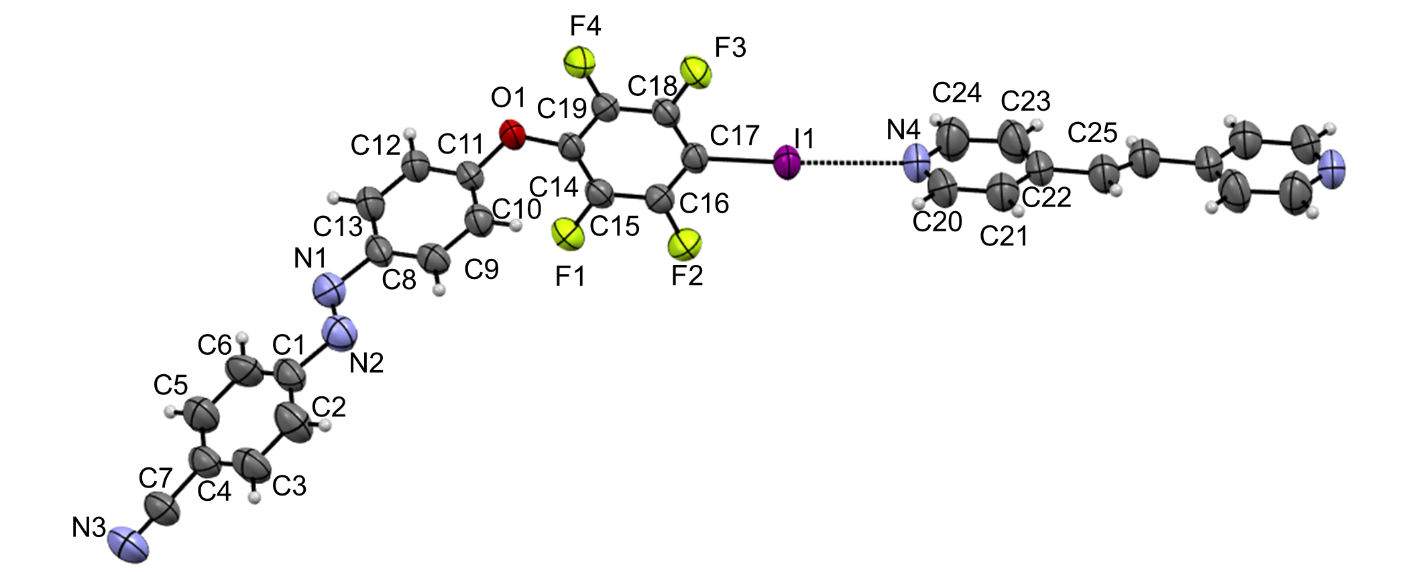


**Figure S11.** Molecular structure of (**IazCN**)_2_(**dpe**) showing the atom-labeling scheme. Displacement ellipsoids are drawn at the 50 % probability level, and H atoms are shown as small spheres of arbitrary radius.


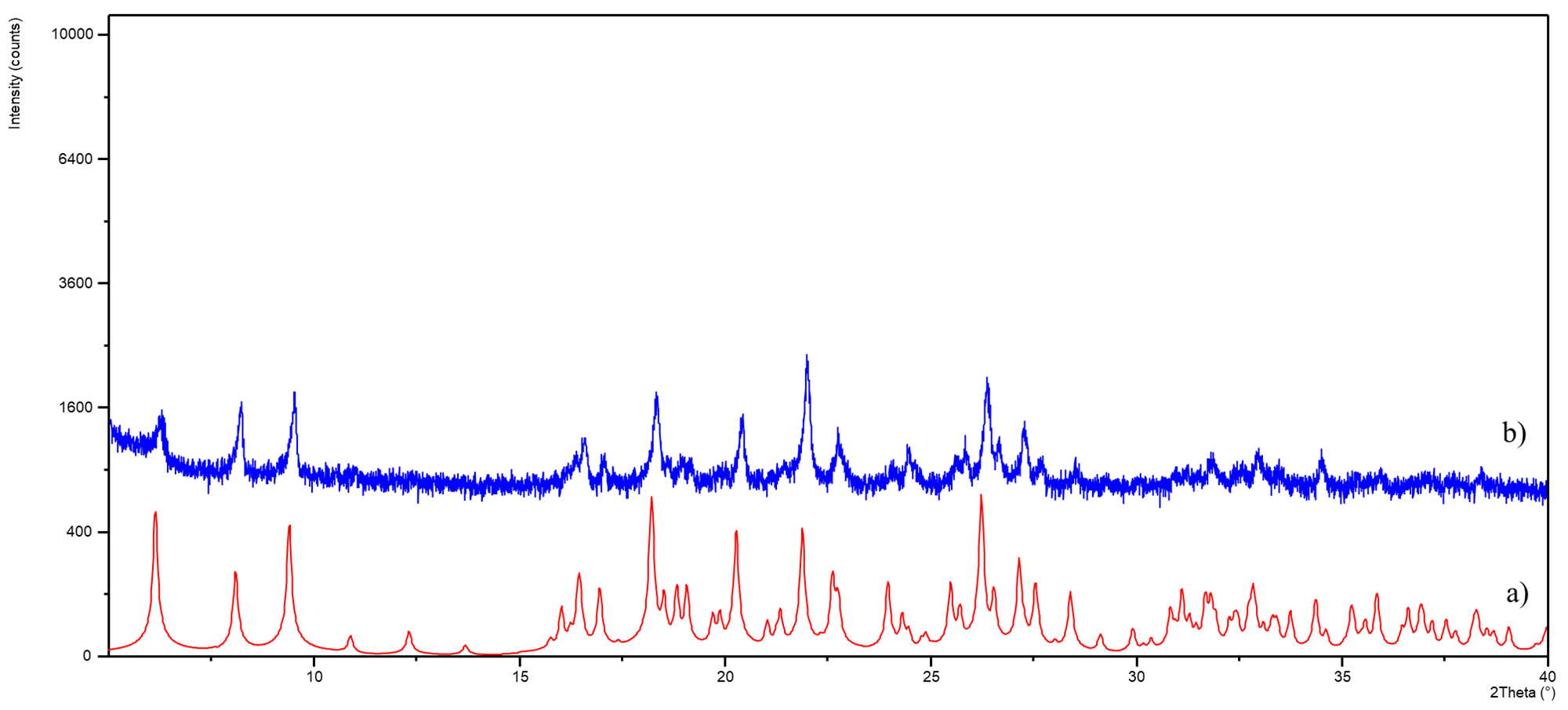


**Figure S12.** PXRD patterns of: a) **IazCN** calculated pattern from single crystal data, b) **IazCN** obtained by synthesis.


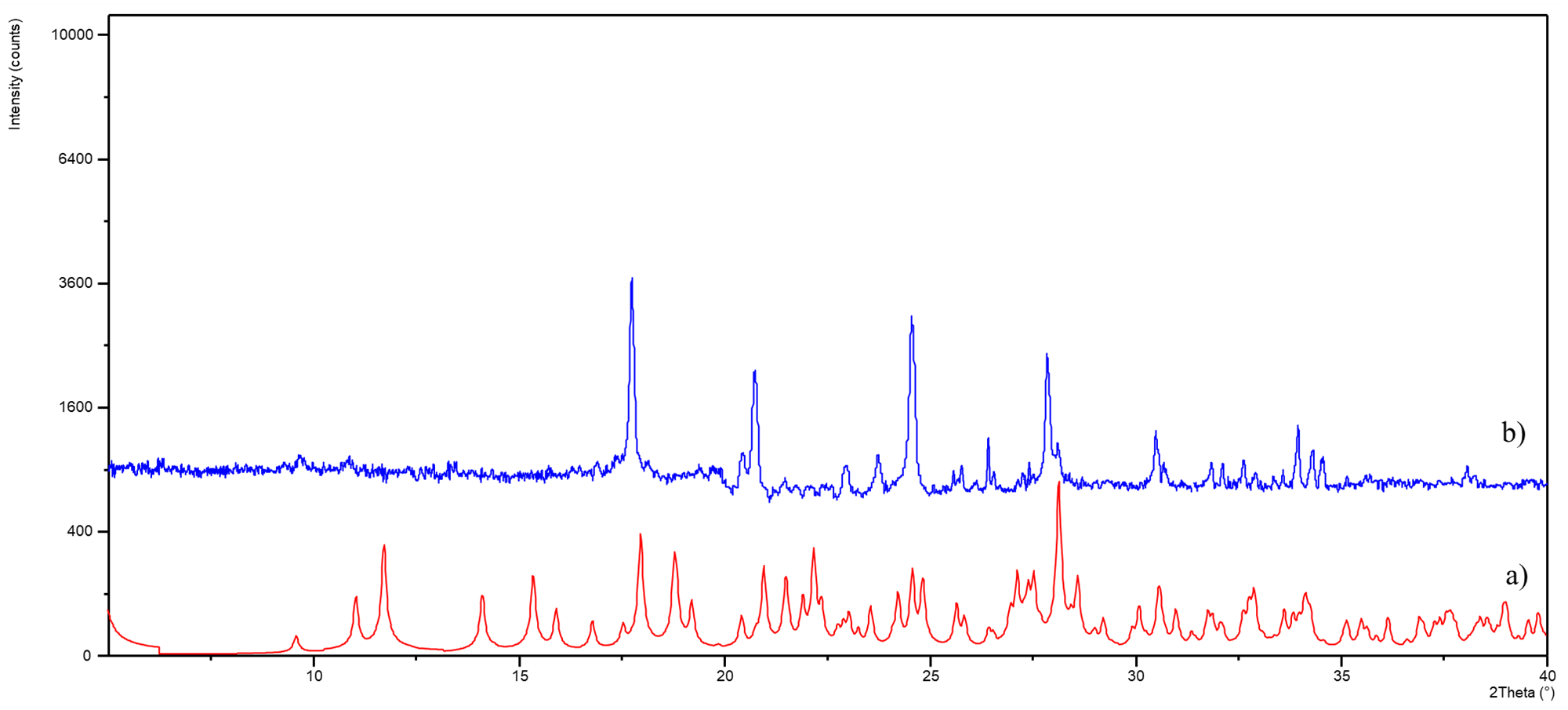


**Figure S13.** PXRD patterns of: a) **IazCl** calculated pattern from single crystal data, b) **IazCl** obtained by synthesis.


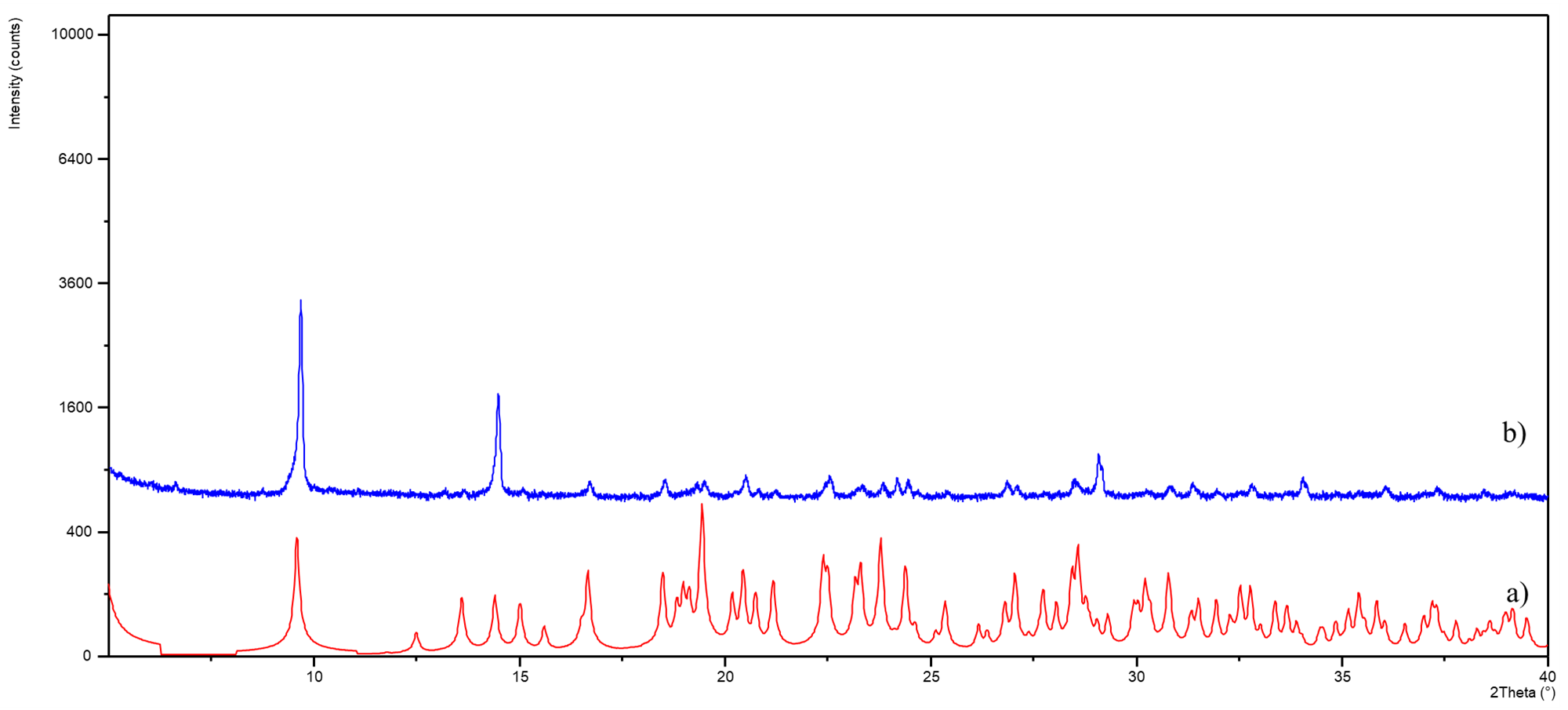


**Figure S14.** PXRD patterns of: a) **IazH** calculated pattern from single crystal data, b) **IazH** obtained by synthesis.


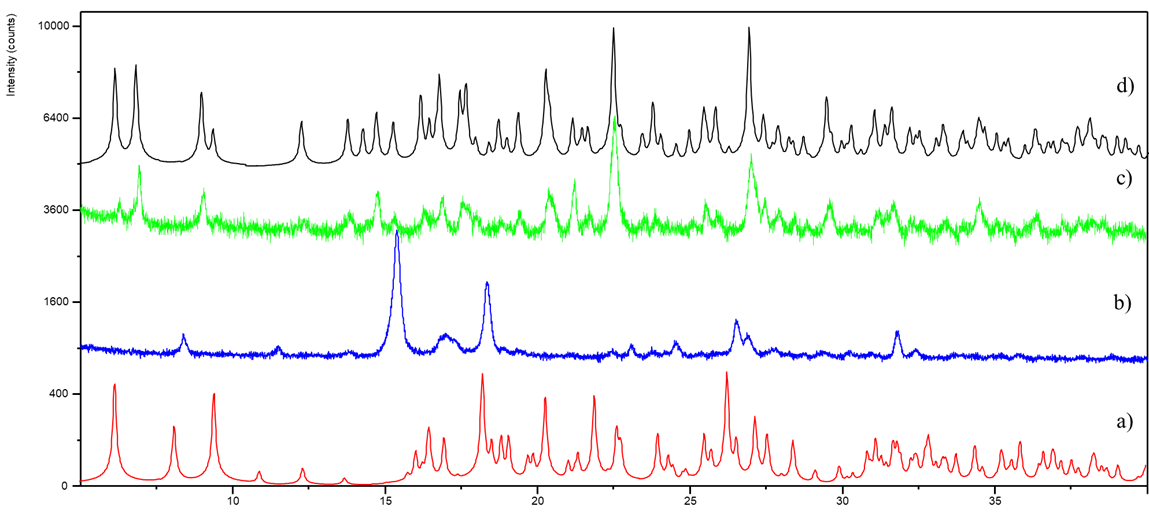


**Figure S15.** PXRD patterns of: a) **IazCN**, b) **44azpy**, c) product obtained by solution crystallization of **44azpy** and **IazCN** in a 1:2 stoichiometric ratio from acetone d) calculated pattern from single crystal data.


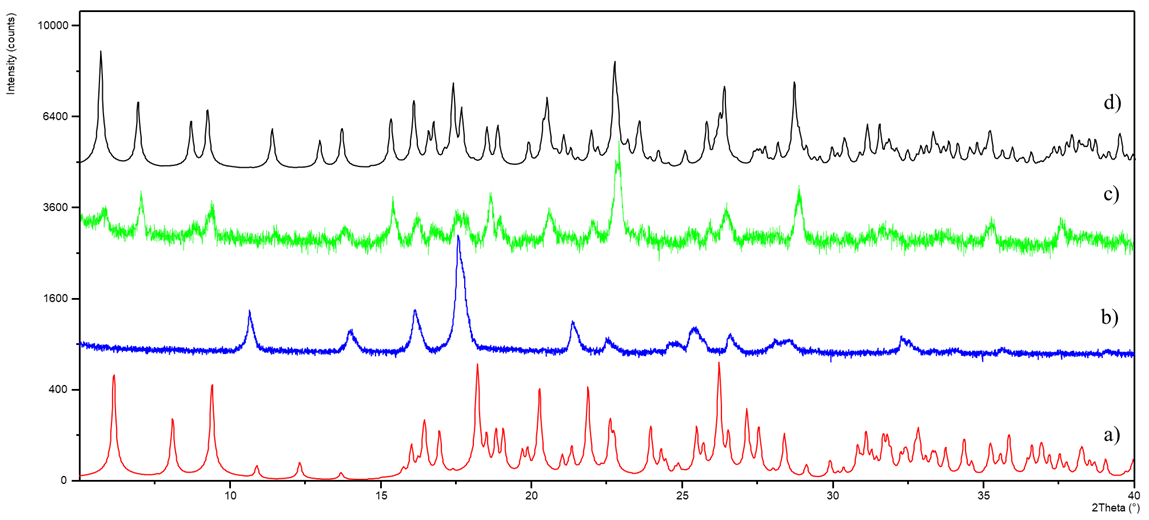


**Figure S16.** PXRD patterns of: a) **IazCN**, b) **hpy**, c) product obtained by solution crystallization of **hpy** and **IazCN** in a 1:2 stoichiometric ratio from acetone d) calculated pattern from single crystal data.


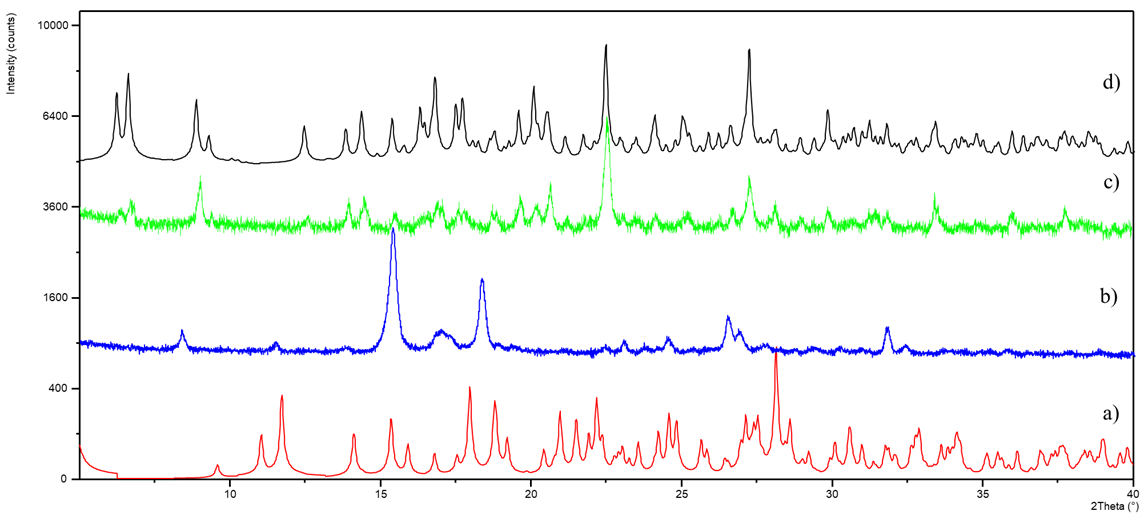


**Figure S17.** PXRD patterns of: a) **IazCl**, b) **44azpy**, c) product obtained by solution crystallization of **44azpy** and **IazCl** in a 1:2 stoichiometric ratio from acetone d) calculated pattern from single crystal data.


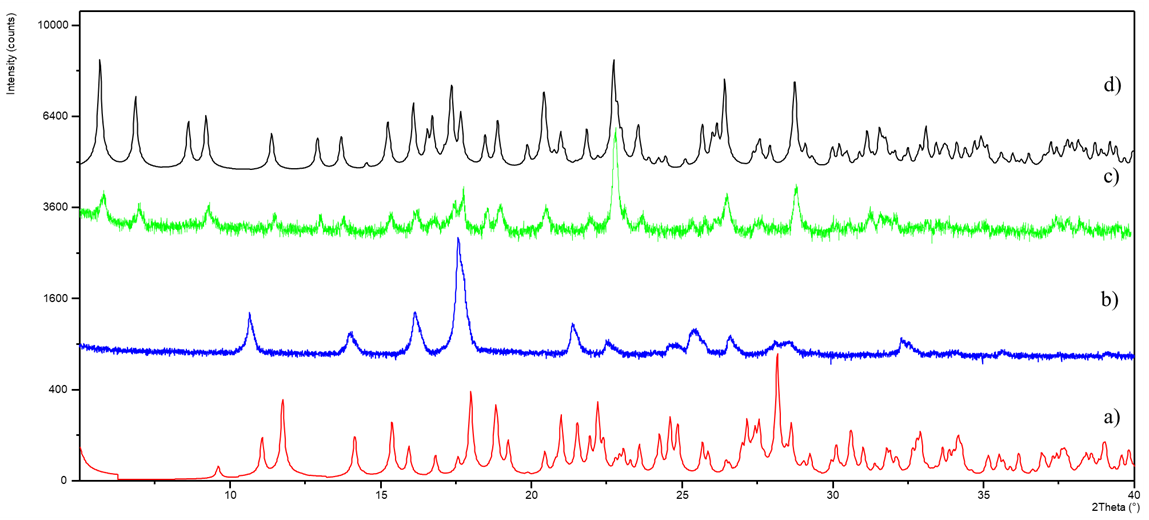


**Figure S18.** PXRD patterns of: a) **IazCl**, b) **hpy**, c) product obtained by solution crystallization of **hpy** and **IazCl** in a 1:2 stoichiometric ratio from acetone d) calculated pattern from single crystal data.


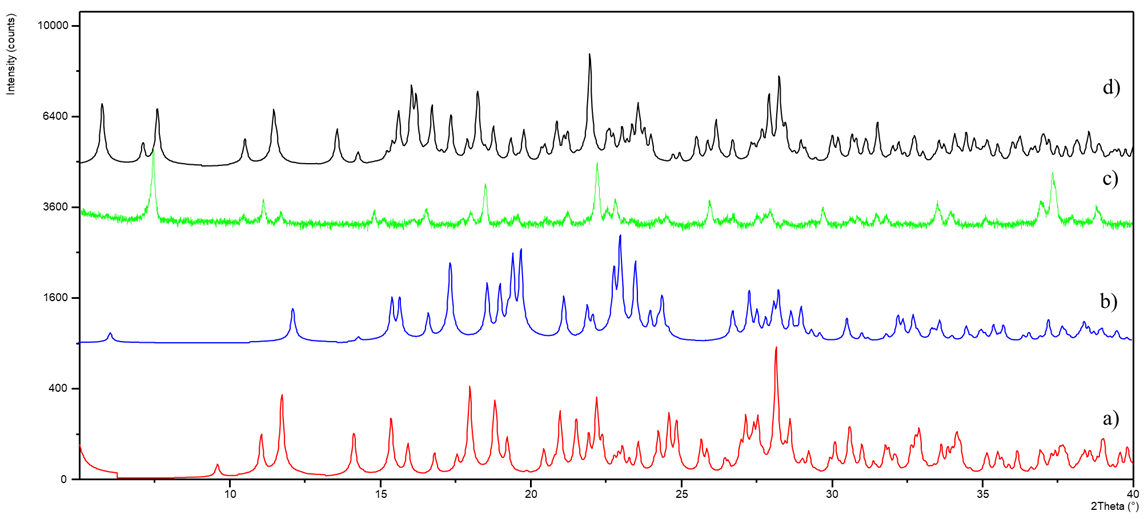


**Figure S19.** PXRD patterns of: a) **IazCl**, b) **dpe**, c) product obtained by solution crystallization of **dpe** and **IazCl** in a 1:2 stoichiometric ratio from nitromethane d) calculated pattern from single crystal data.


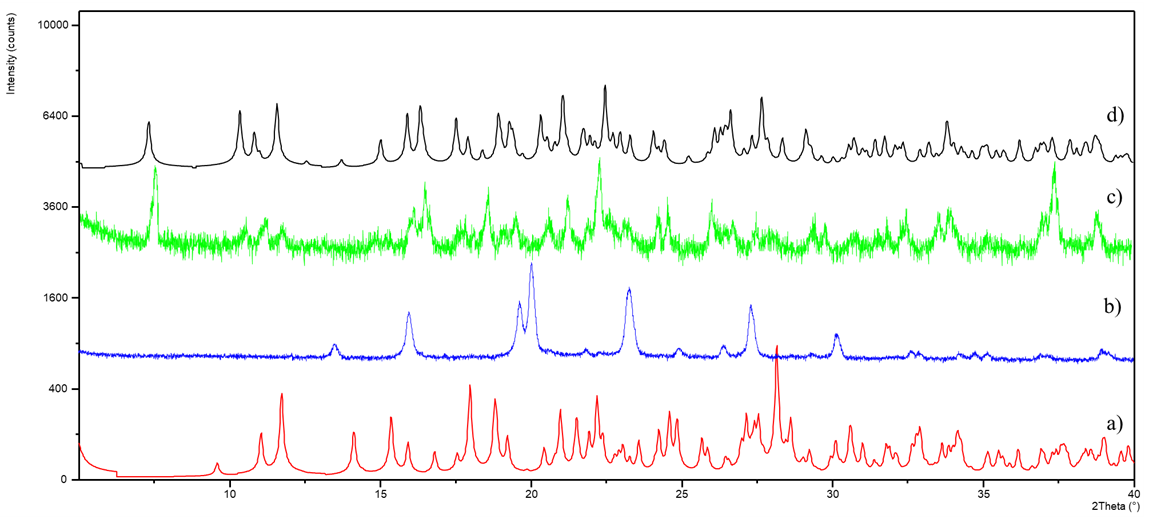


**Figure S20.** PXRD patterns of: a) **IazCl**, b) **dpa**, c) product obtained by solution crystallization of **dpa** and **IazCl** in a 1:2 stoichiometric ratio from acetonitrile d) calculated pattern from single crystal data.

**
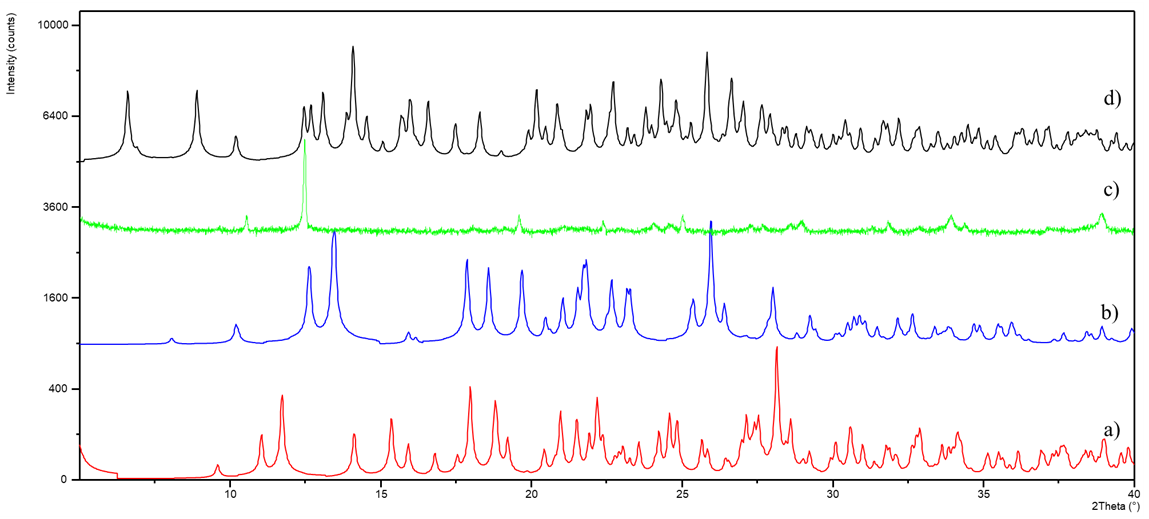
**

**Figure S21**. PXRD patterns of: a) **IazCl**, b) **44bpy**, c) product obtained by solution crystallization of **44bpy** and **IazCl** in a 1:2 stoichiometric ratio from EtOH d) calculated pattern from single crystal data.


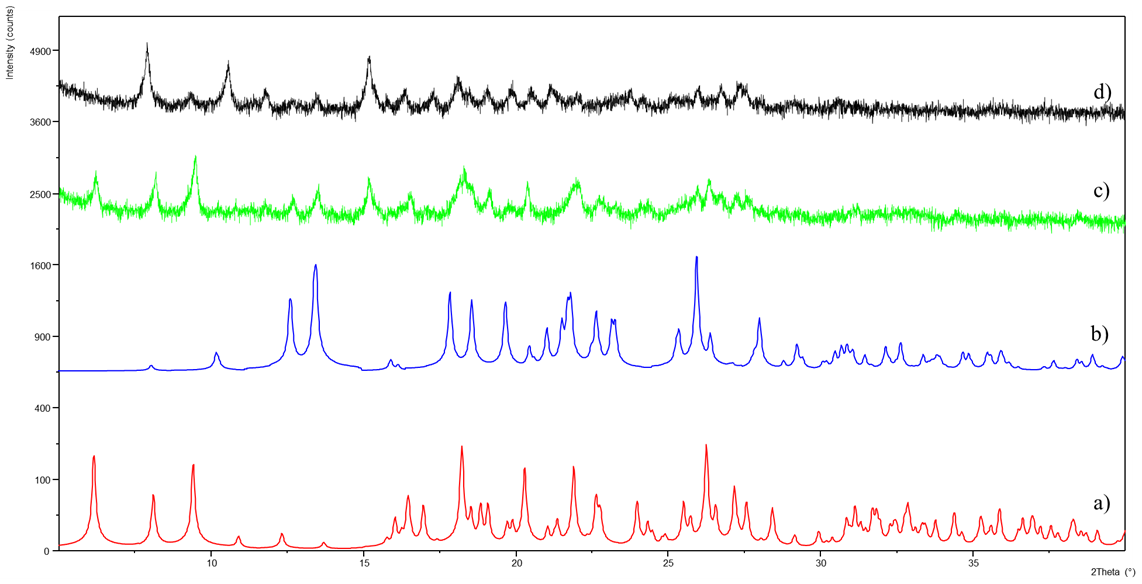


**Figure S22**. PXRD patterns of: a) **IazCN**, b) **44bpy**, c) product obtained by solution crystallization of **44azpy** and **IazCN** in a 1:2 stoichiometric ratio from acetone d) product obtained by solution crystallization of **44azpy** and **IazCN** in a 1:2 stoichiometric ratio from ethanol.


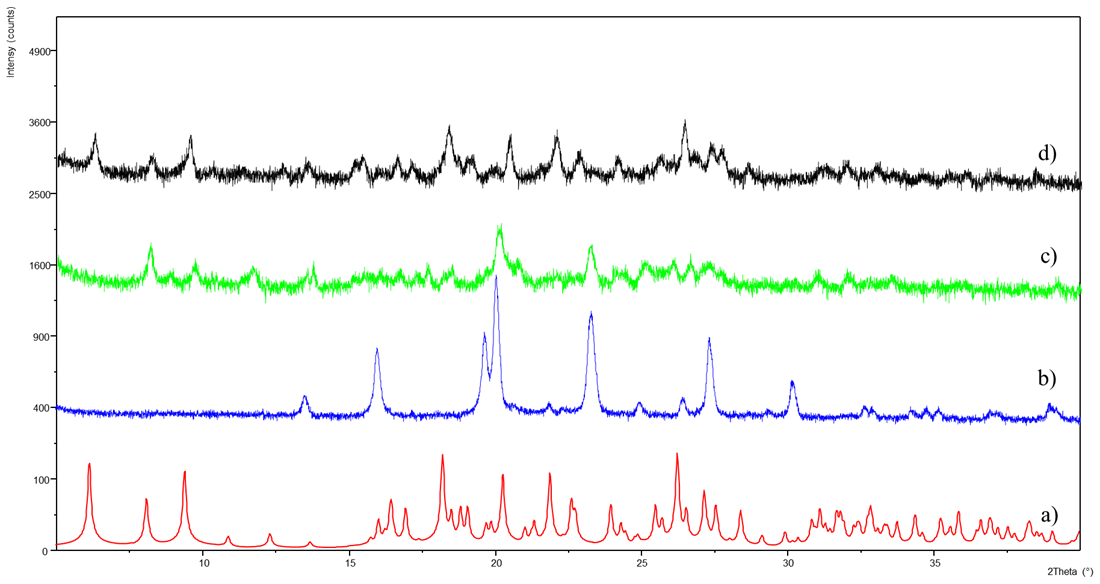


**Figure S23.** PXRD patterns of: a) **IazCN**, b) **dpa**, c) product obtained by solution crystallization of **dpa** and **IazCN** in a 1:2 stoichiometric ratio from acetone d) product obtained by solution crystallization of **dpa** and **IazCN** in a 1:2 stoichiometric ratio from ethanol.


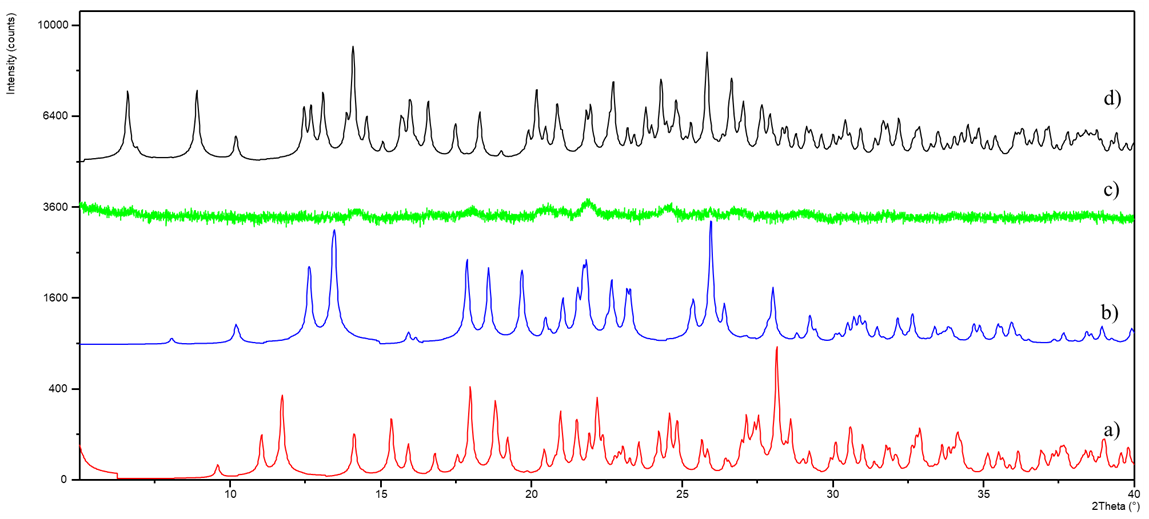


**Figure S24.** PXRD patterns of: a) **IazCl**, b) **44bpy**, c) product obtained by grinding **44bpy** and **IazCl** in a 1:2 stoichiometric ratio d) calculated pattern from single crystal data.


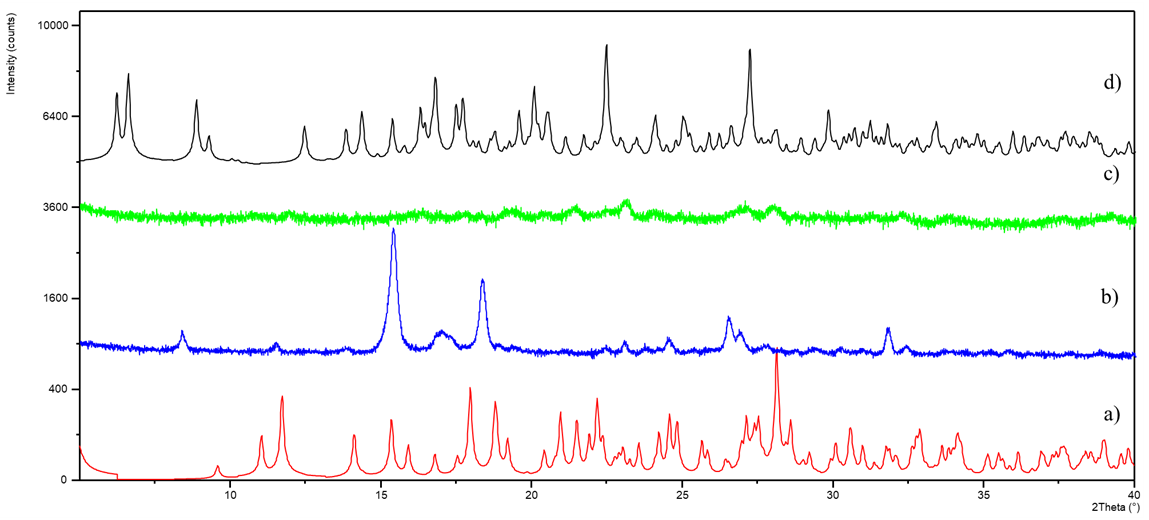


**Figure S25.** PXRD patterns of: a) **IazCl**, b) **44azpy**, c) product obtained by grinding **44azpy** and **IazCl** in a 1:2 stoichiometric ratio d) calculated pattern from single crystal data.


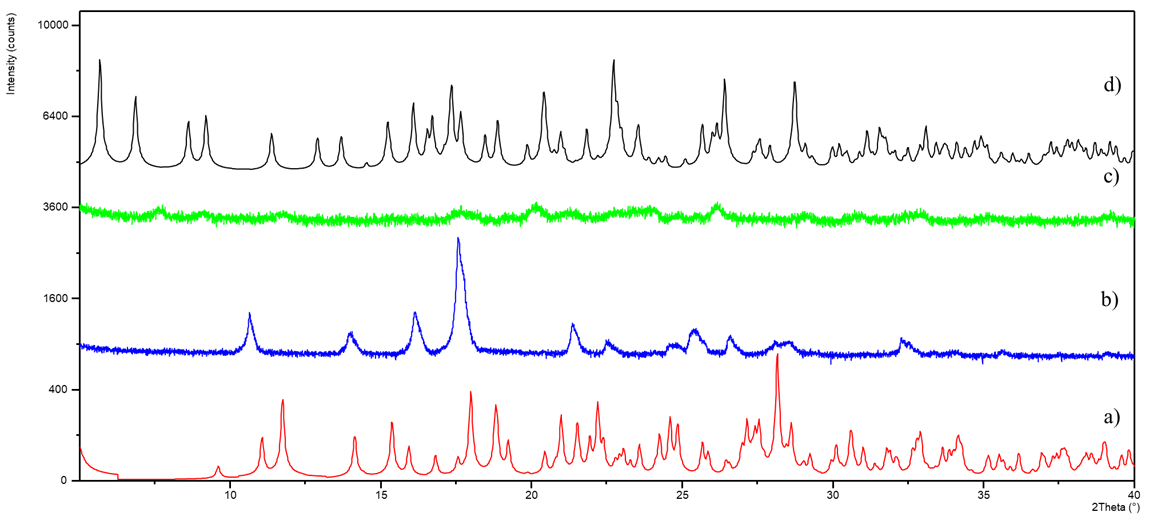


**Figure S26.** PXRD patterns of: a) **IazCl**, b) **hpy**, c) product obtained by grinding **hpy** and **IazCl** in a 1:2 stoichiometric ratio d) calculated pattern from single crystal data.

**
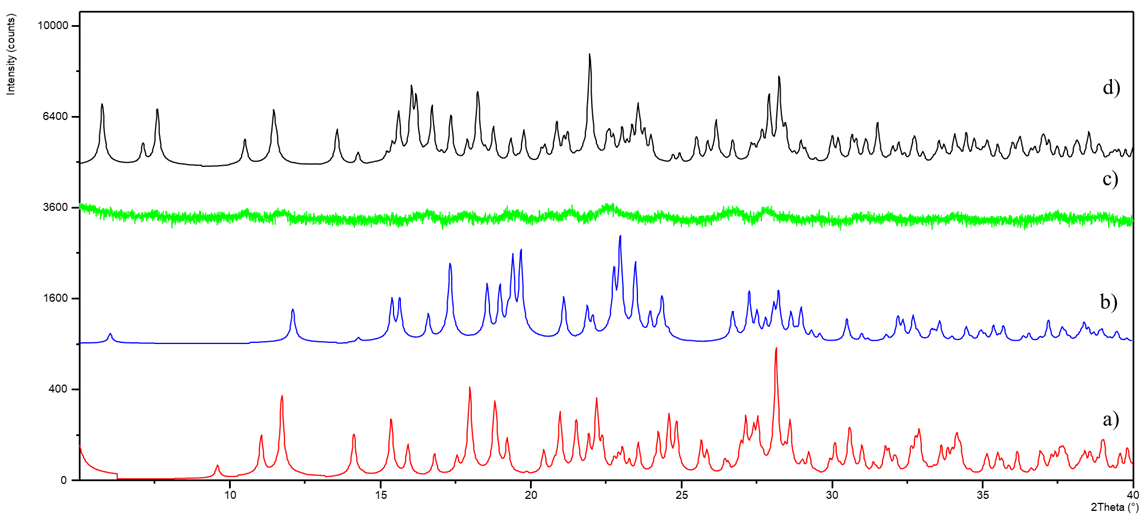
**

**Figure S27.** PXRD patterns of: a) **IazCl**, b) **dpe**, c) product obtained by grinding **dpe** and **IazCl** in a 1:2 stoichiometric ratio d) calculated pattern from single crystal data.


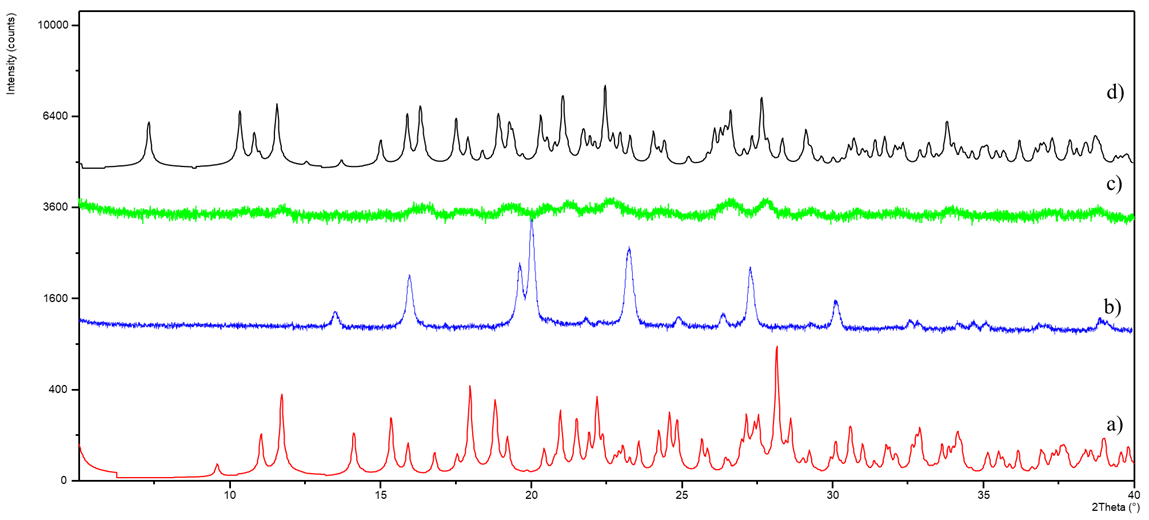


**Figure S28.** PXRD patterns of: a) **IazCl**, b) **dpa**, c) product obtained by grinding **dpa** and **IazCl** in a 1:2 stoichiometric ratio d) calculated pattern from single crystal data.

**
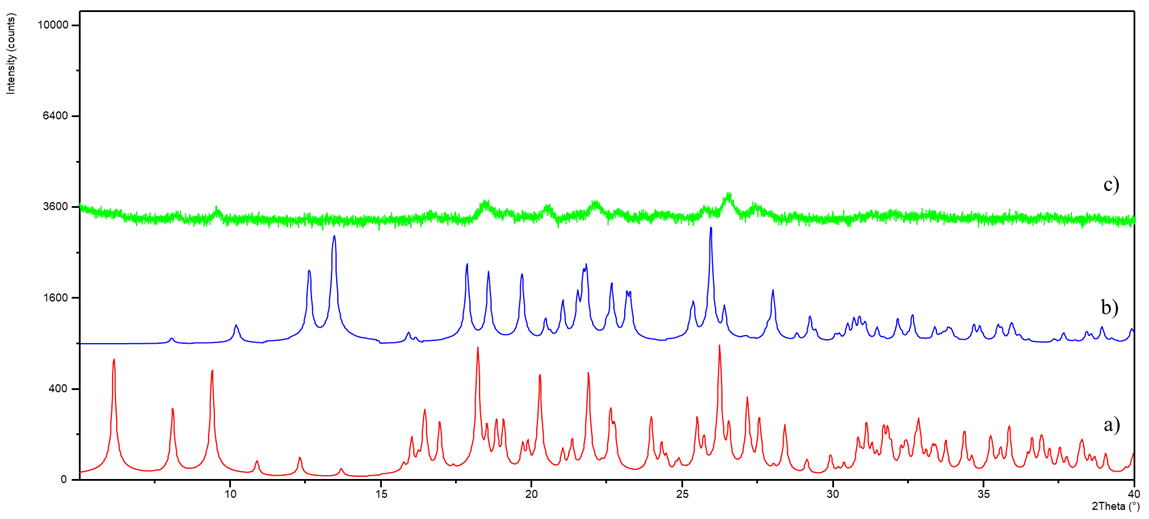
**

**Figure S29.** PXRD patterns of: a) **IazCN**, b) **44bpy**, c) product obtained by grinding **44bpy** and **IazCN** in a 1:2 stoichiometric ratio.


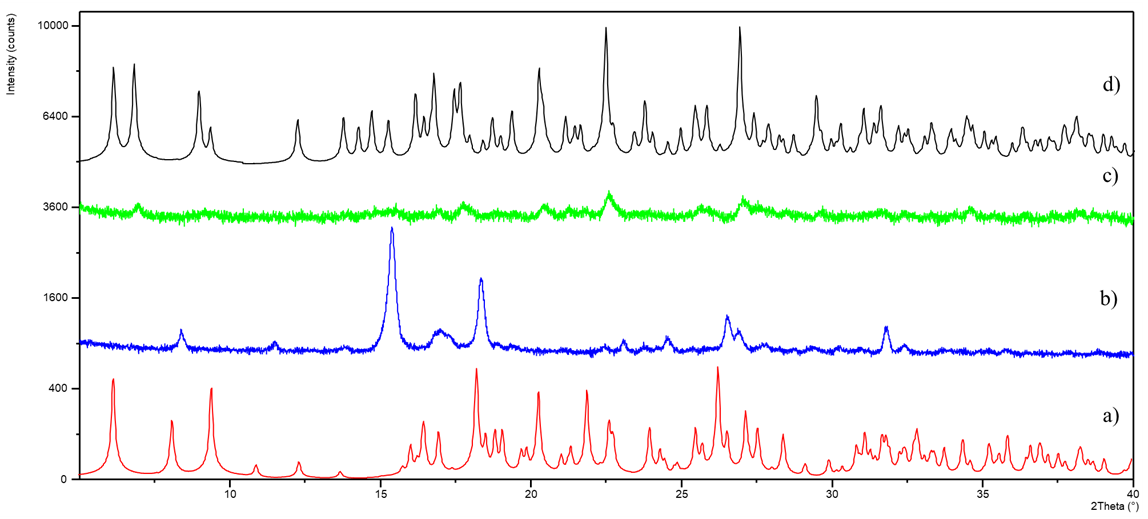


**Figure S30.** PXRD patterns of: a) **IazCN**, b) **44azpy**, c) product obtained by grinding **44azpy** and **IazCN** in a 1:2 stoichiometric ratio d) calculated pattern from single crystal data.


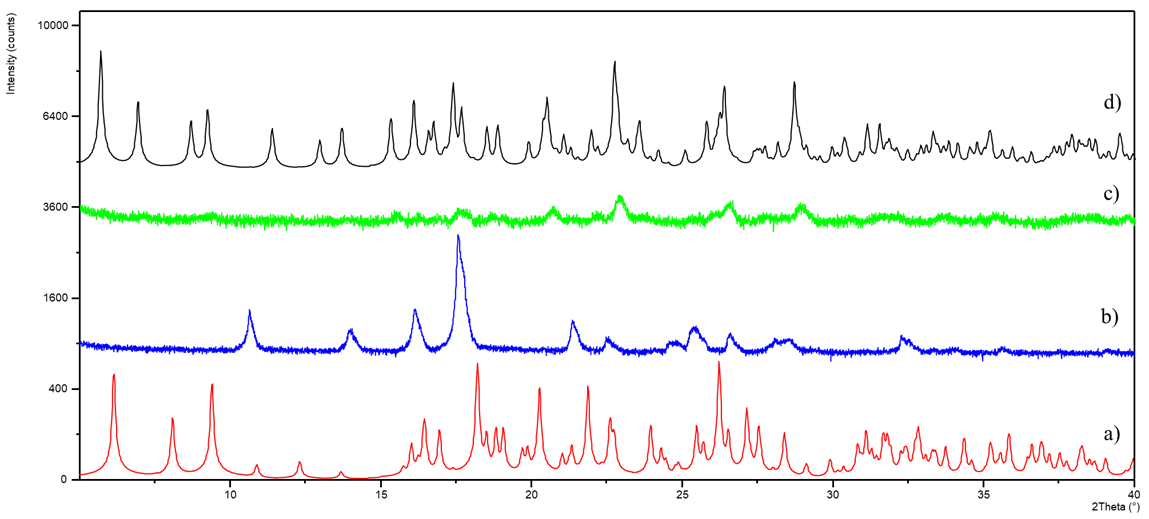


**Figure S31.** PXRD patterns of: a) **IazCN**, b) **hpy**, c) product obtained by grinding **hpy** and **IazCN** in a 1:2 stoichiometric ratio d) calculated pattern from single crystal data.


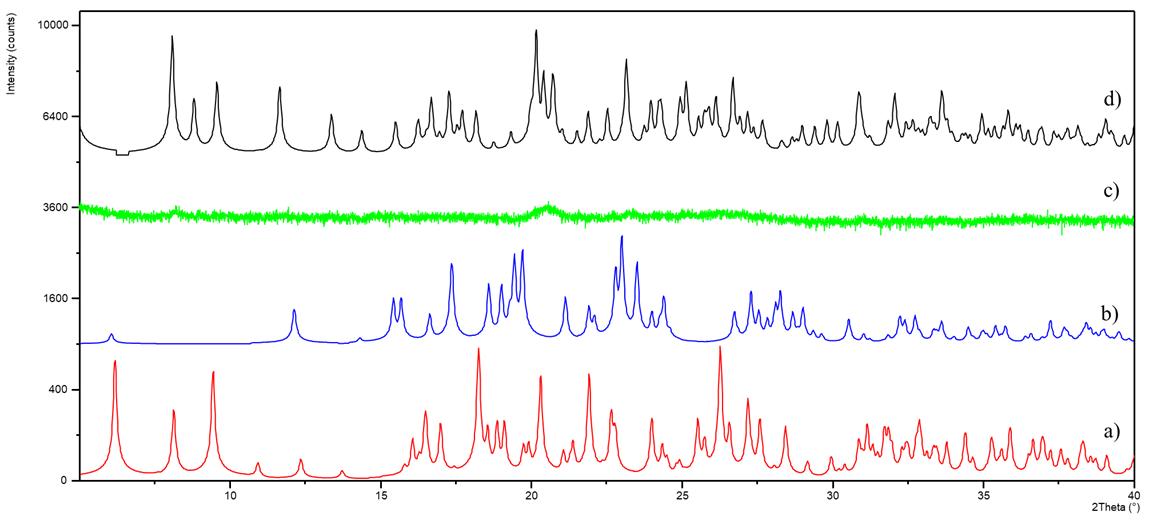


**Figure S32.** PXRD patterns of: a) **IazCN**, b) **dpe**, c) product obtained by grinding **dpe** and **IazCN** in a 1:2 stoichiometric ratio d) calculated pattern from single crystal data.


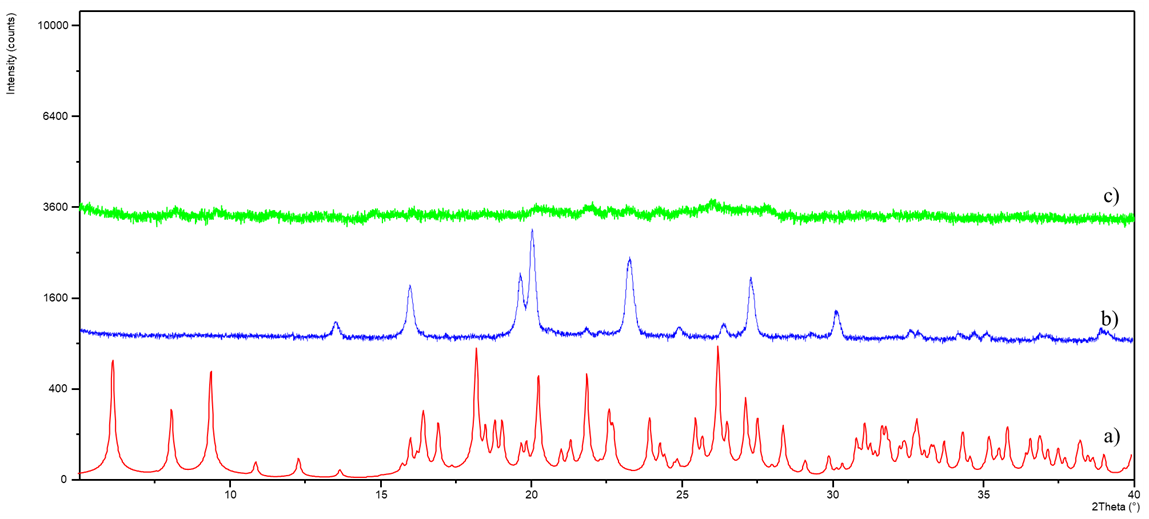


**Figure S33.** PXRD patterns of: a) **IazCN**, b) **dpa**, c) product obtained by grinding **dpa** and **IazCN** in a 1:2 stoichiometric ratio.


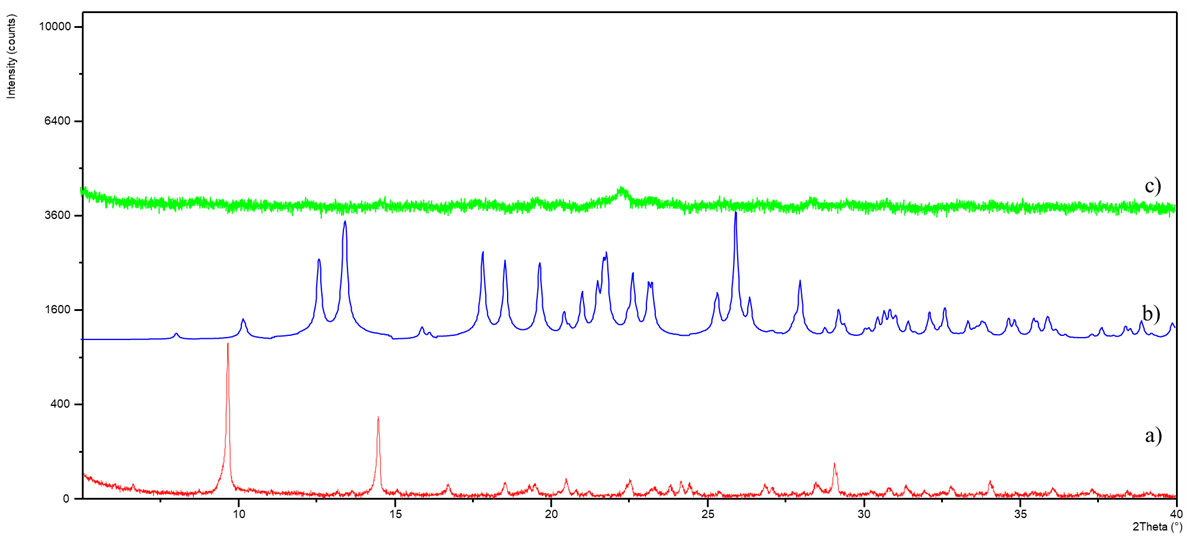


**Figure S34.** PXRD patterns of: a) **IazH**, b) **44bpy**, c) product obtained by grinding **44bpy** and **IazH** in a 1:2 stoichiometric ratio.


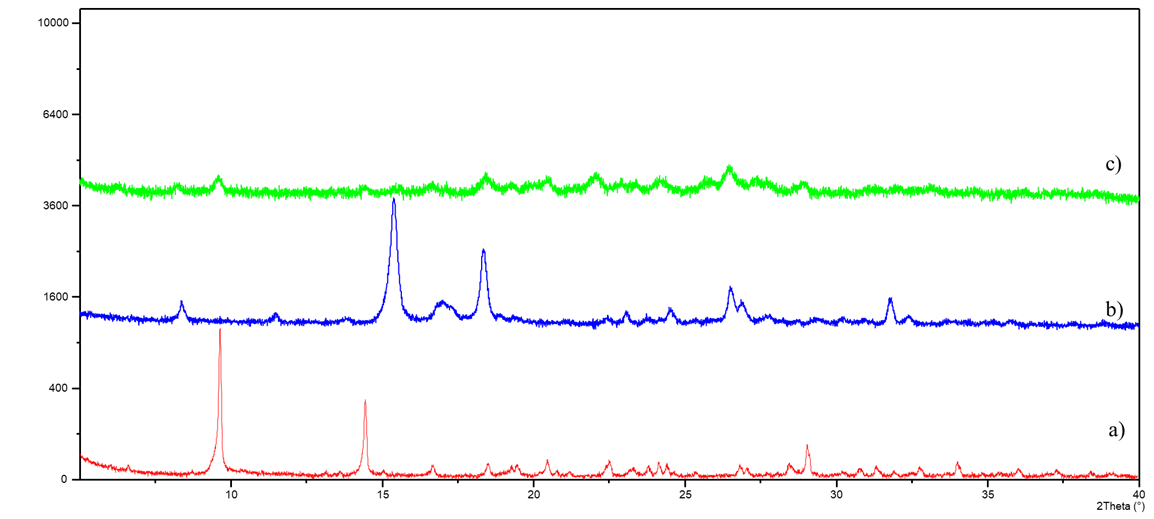


**Figure S35.** PXRD patterns of: a) **IazH**, b) **44azpy**, c) product obtained by grinding **44azpy** and **IazH** in a 1:2 stoichiometric ratio.


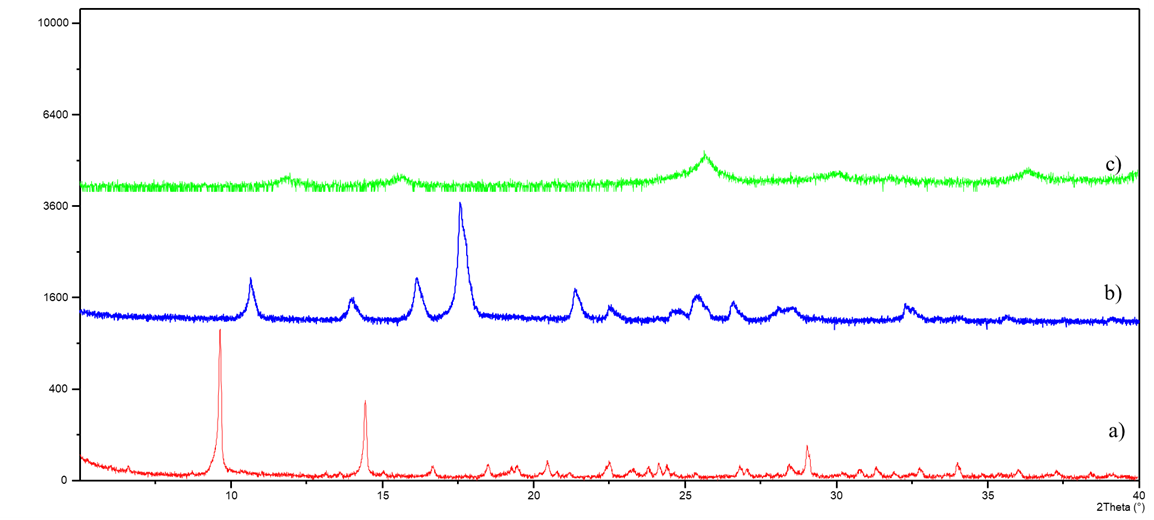


**Figure S36.** PXRD patterns of: a) **IazH**, b) **hpy**, c) product obtained by grinding **hpy** and **IazH** in a 1:2 stoichiometric ratio.


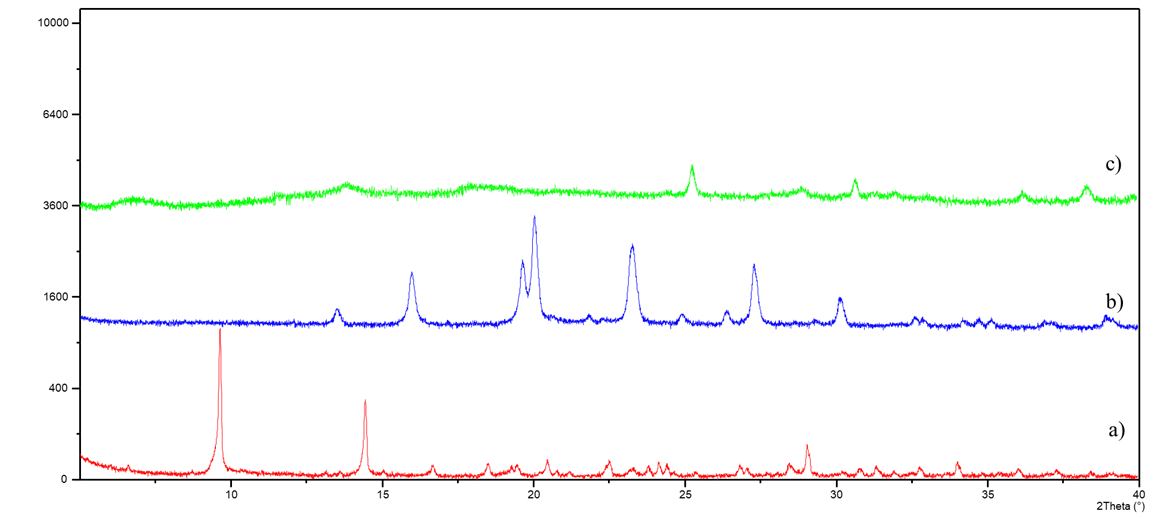


**Figure S37.** PXRD patterns of: a) **IazH**, b) **dpa**, c) product obtained by grinding **dpa** and **IazH** in a 1:2 stoichiometric ratio.


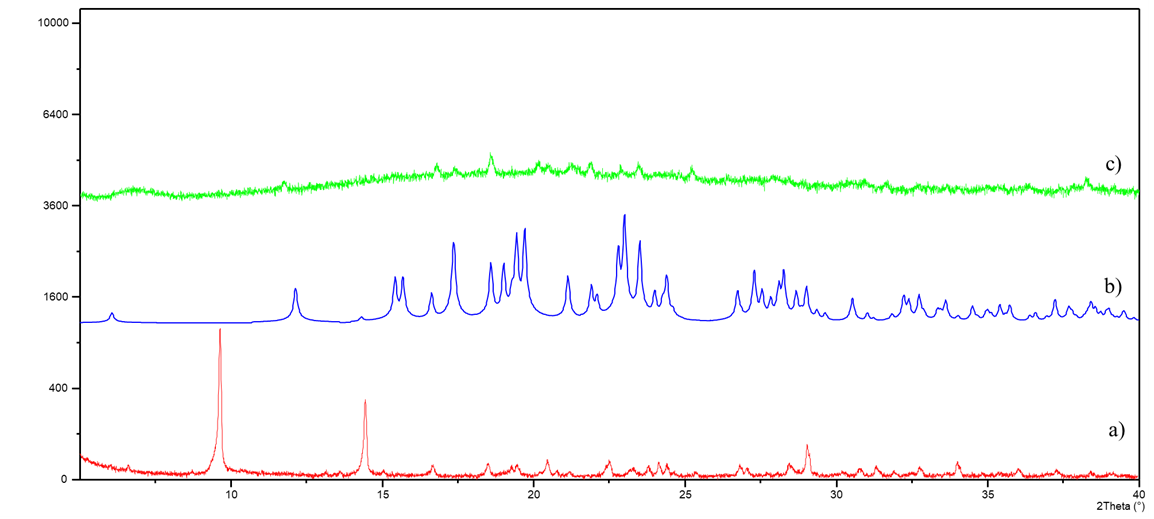


**Figure S38.** PXRD patterns of: a) **IazH**, b) **dpe**, c) product obtained by grinding **dpe** and **IazH** in a 1:2 stoichiometric ratio.


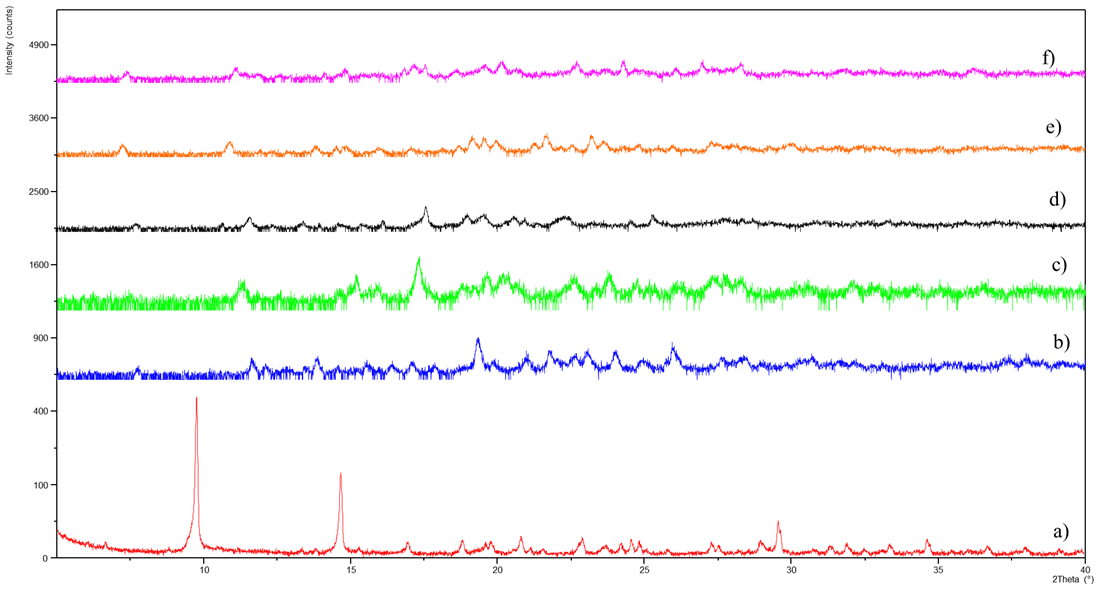


**Figure S39.** PXRD patterns of: a) **IazH**, products obtained by solution crystallization of **IazH** and b) **44bpy**, c) **44azpy**, d) **hpy**, e) **dpe**, f) **dpa** in a 2:1 stoichiometric ratio from ethanol.


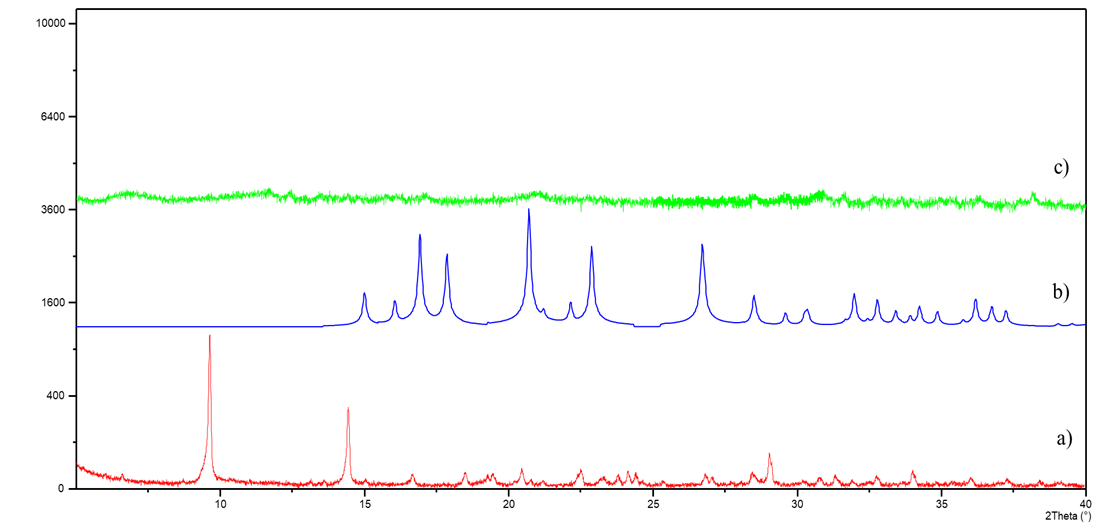


**Figure S40.** PXRD patterns of: a) **IazH**, b) **22bpy**, c) product obtained by grinding **22bpy** and **IazH** in a 1:2 stoichiometric ratio.


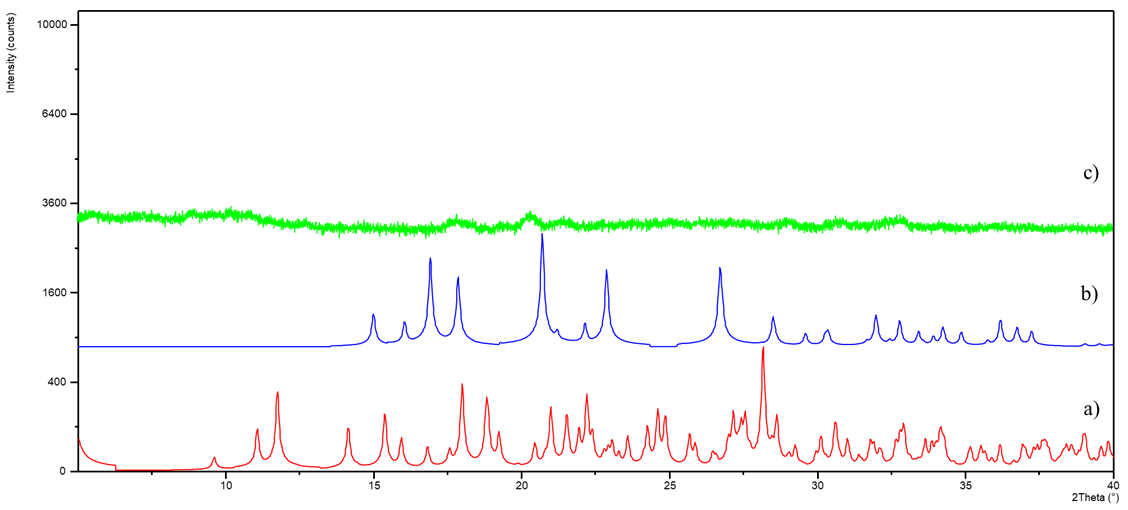


**Figure S41.** PXRD patterns of: a) **IazCl**, b) **22bpy**, c) product obtained by grinding **22bpy** and **IazCl** in a 1:2 stoichiometric ratio.


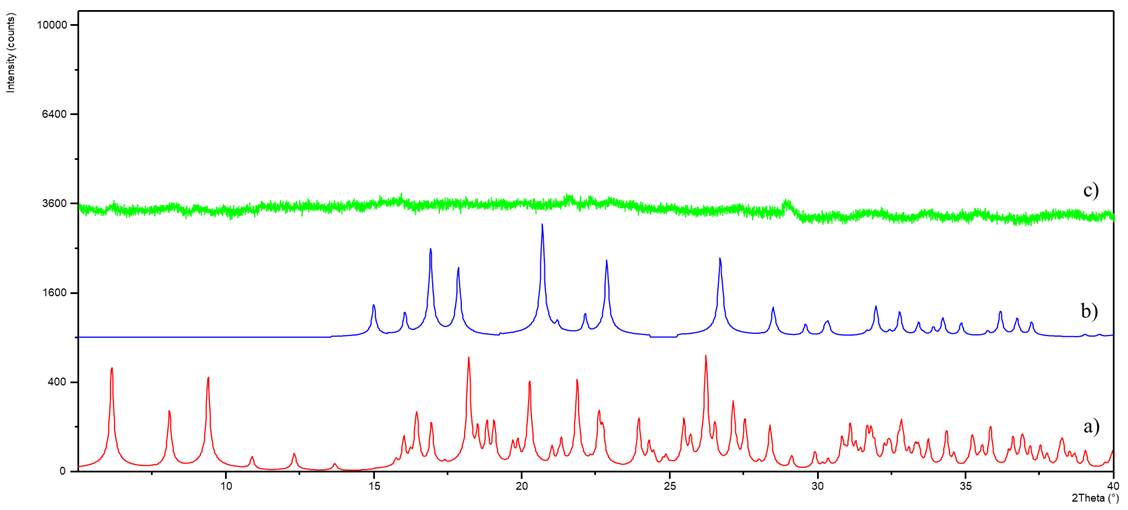


**Figure S42.** PXRD patterns of: a) **IazCN**, b) **22bpy**, c) product obtained by grinding **22bpy** and **IazCN** in a 1:2 stoichiometric ratio.


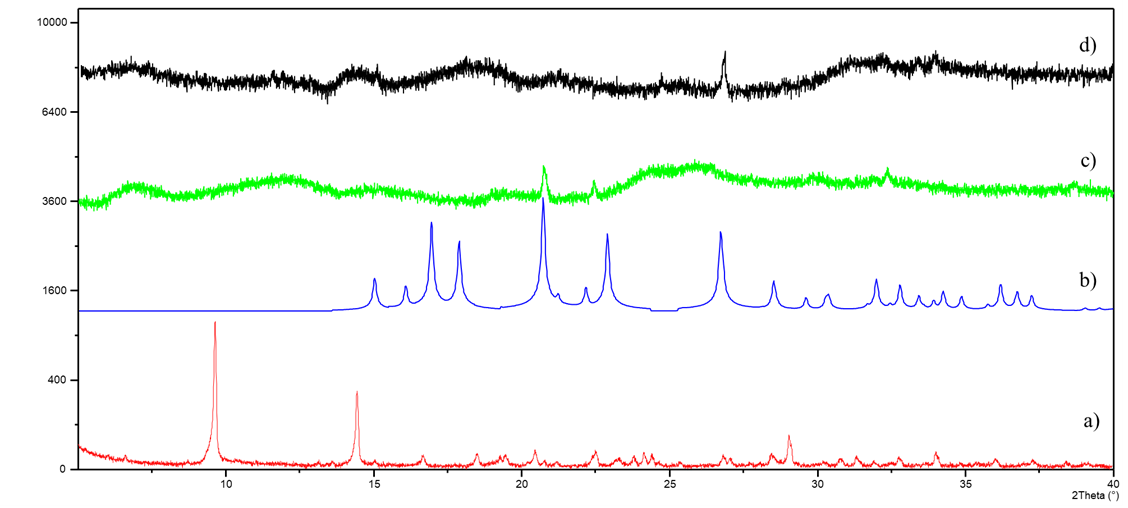


**Figure S43.** PXRD patterns of: a) **IazH**, b) **22bpy**, c) product obtained by solution crystallization of **22bpy** and **IazH** in a 1:2 stoichiometric ratio from ethanol, d) product obtained by solution crystallization of **22bpy** and **IazH** in a 1:2 stoichiometric ratio from acetone.


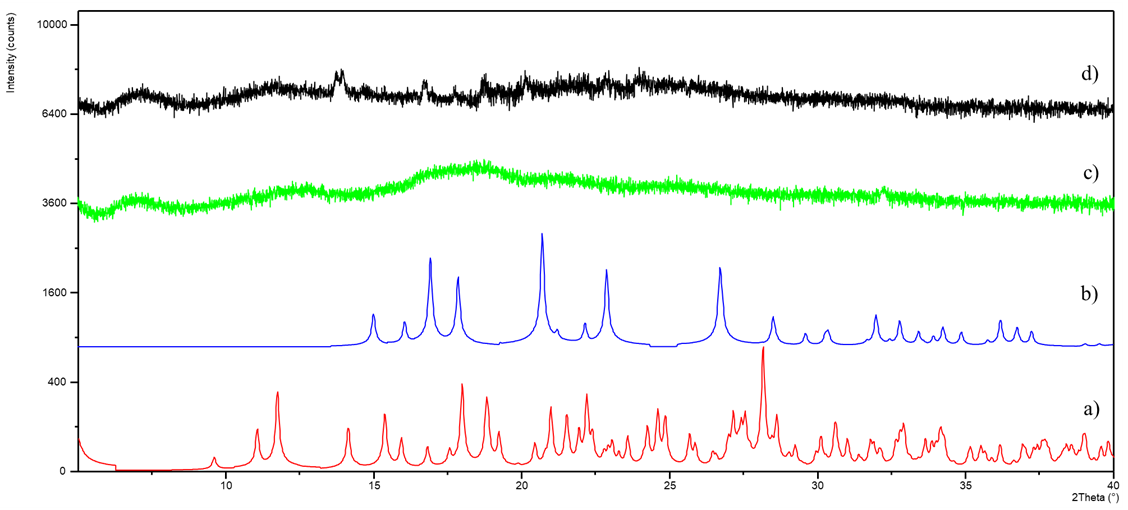


**Figure S44.** PXRD patterns of: a) **IazCl**, b) **22bpy**, c) product obtained by solution crystallization of **22bpy** and **IazCl** in a 1:2 stoichiometric ratio from ethanol, d) product obtained by solution crystallization of **22bpy** and **IazCl** in a 1:2 stoichiometric ratio from acetone.


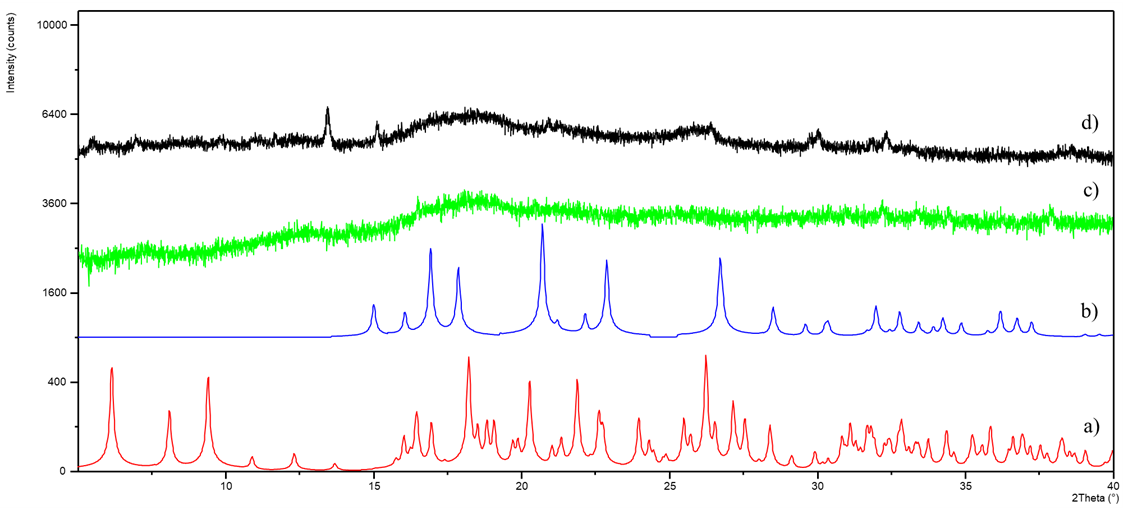


**Figure S45.** PXRD patterns of: a) **IazCN**, b) **22bpy**, c) product obtained by solution crystallization of **22bpy** and **IazCN** in a 1:2 stoichiometric ratio from ethanol, d) product obtained by solution crystallization of **22bpy** and **IazCN** in a 1:2 stoichiometric ratio from acetone.


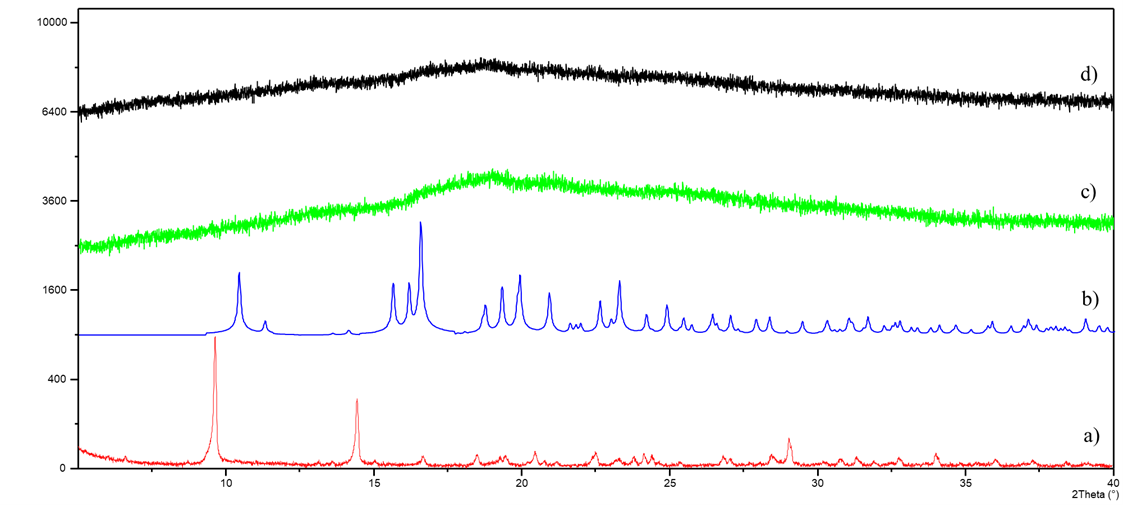


**Figure S46.** PXRD patterns of: a) **IazH**, b) **dmap**, c) product obtained by solution crystallization of **dmap** and **IazH** in a 1:1 stoichiometric ratio from ethanol, d) product obtained by solution crystallization of **dmap** and **IazH** in a 1:1 stoichiometric ratio from acetone.


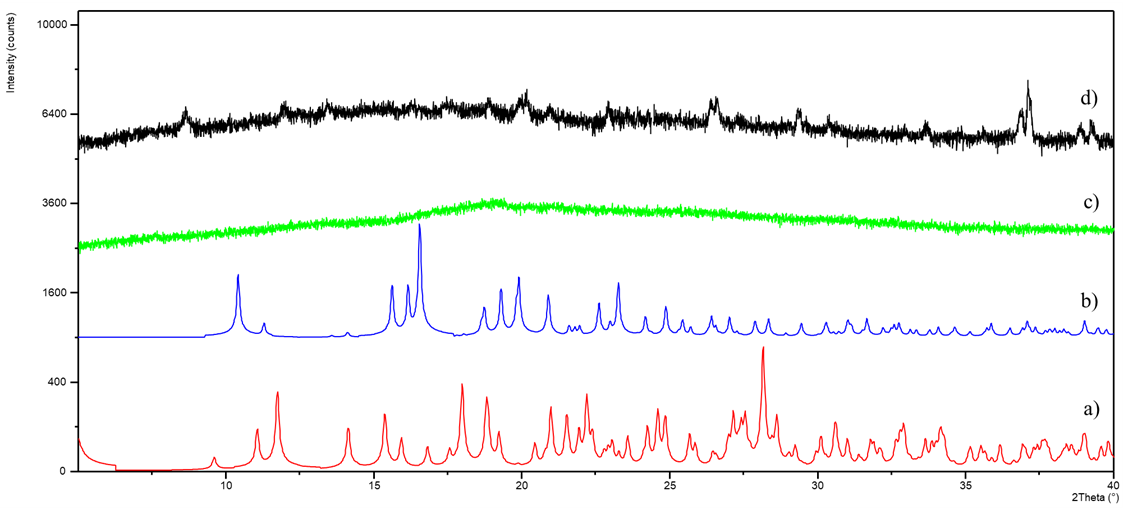


**Figure S47.** PXRD patterns of: a) **IazCl**, b) **dmap**, c) product obtained by solution crystallization of **dmap** and **IazCl** in a 1:1 stoichiometric ratio from ethanol, d) product obtained by solution crystallization of **dmap** and **IazCl** in a 1:1 stoichiometric ratio from acetone.


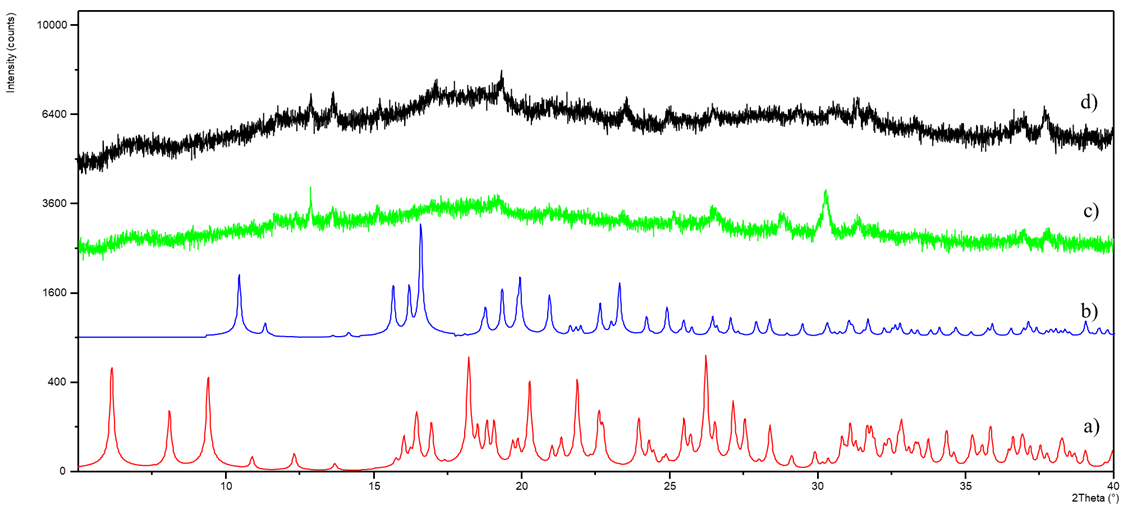


**Figure S48.** PXRD patterns of: a) **IazCN**, b) **dmap**, c) product obtained by solution crystallization of **dmap** and **IazCN** in a 1:1 stoichiometric ratio from ethanol, d) product obtained by solution crystallization of **dmap** and **IazCN** in a 1:1 stoichiometric ratio from acetone.


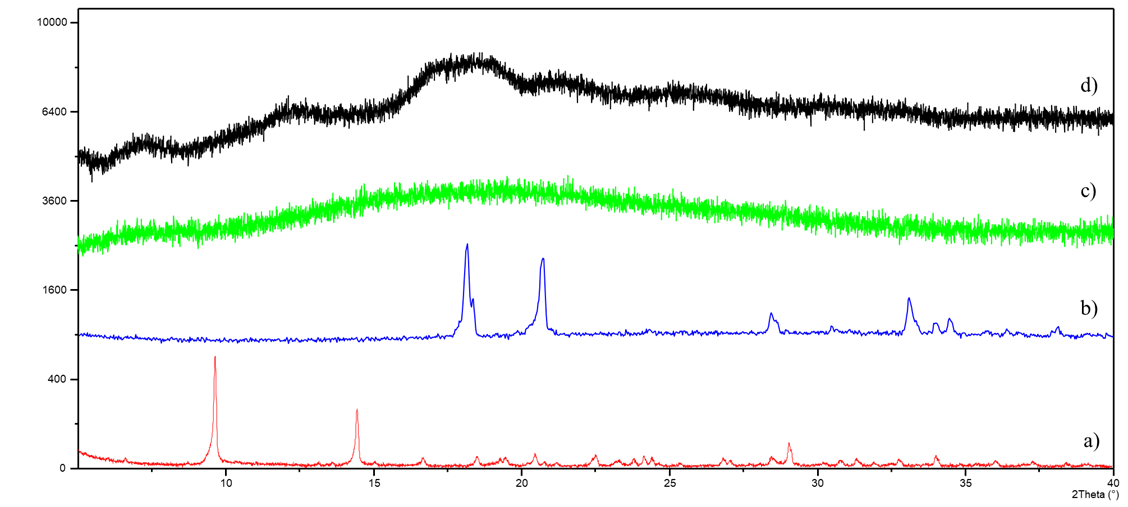


**Figure S49.** PXRD patterns of: a) **IazH**, b) **dabco**, c) product obtained by solution crystallization of **dabco** and **IazH** in a 1:2 stoichiometric ratio from ethanol, d) product obtained by solution crystallization of **dabco** and **IazH** in a 1:2 stoichiometric ratio from acetone.


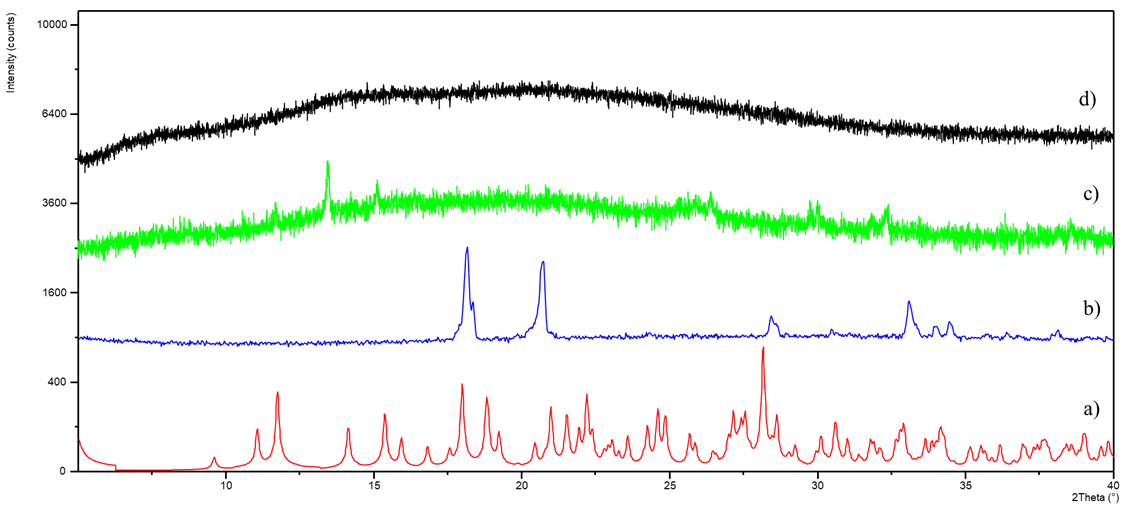


**Figure S50.** PXRD patterns of: a) **IazCl**, b) **dabco**, c) product obtained by solution crystallization of **dabco** and **IazCl** in a 1:2 stoichiometric ratio from ethanol, d) product obtained by solution crystallization of **dabco** and **IazCl** in a 1:2 stoichiometric ratio from acetone.


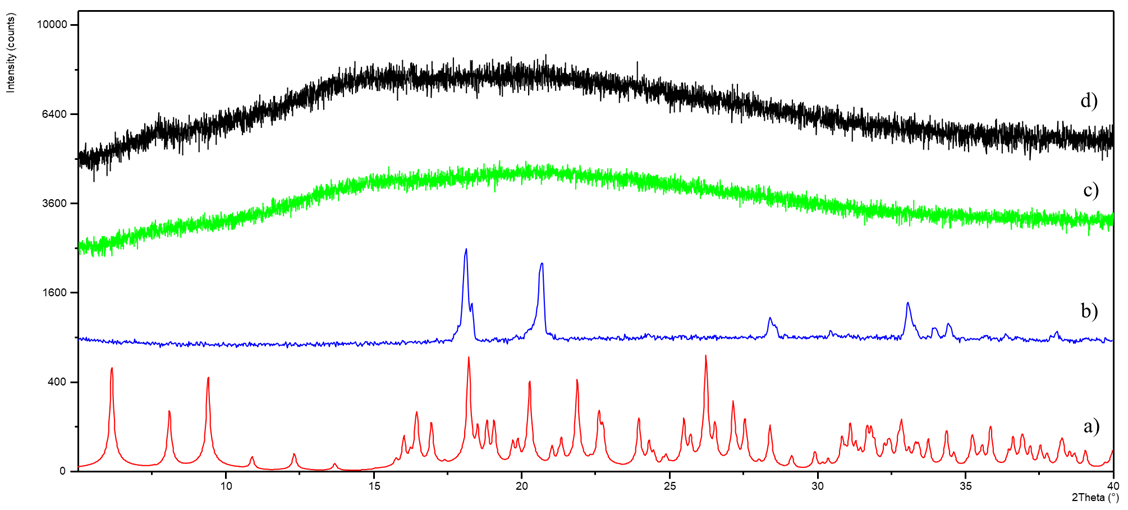


**Figure S51.** PXRD patterns of: a) **IazCN**, b) **dabco**, c) product obtained by solution crystallization of **dabco** and **IazCN** in a 1:2 stoichiometric ratio from ethanol, d) product obtained by solution crystallization of **dabco** and **IazCN** in a 1:2 stoichiometric ratio from acetone.


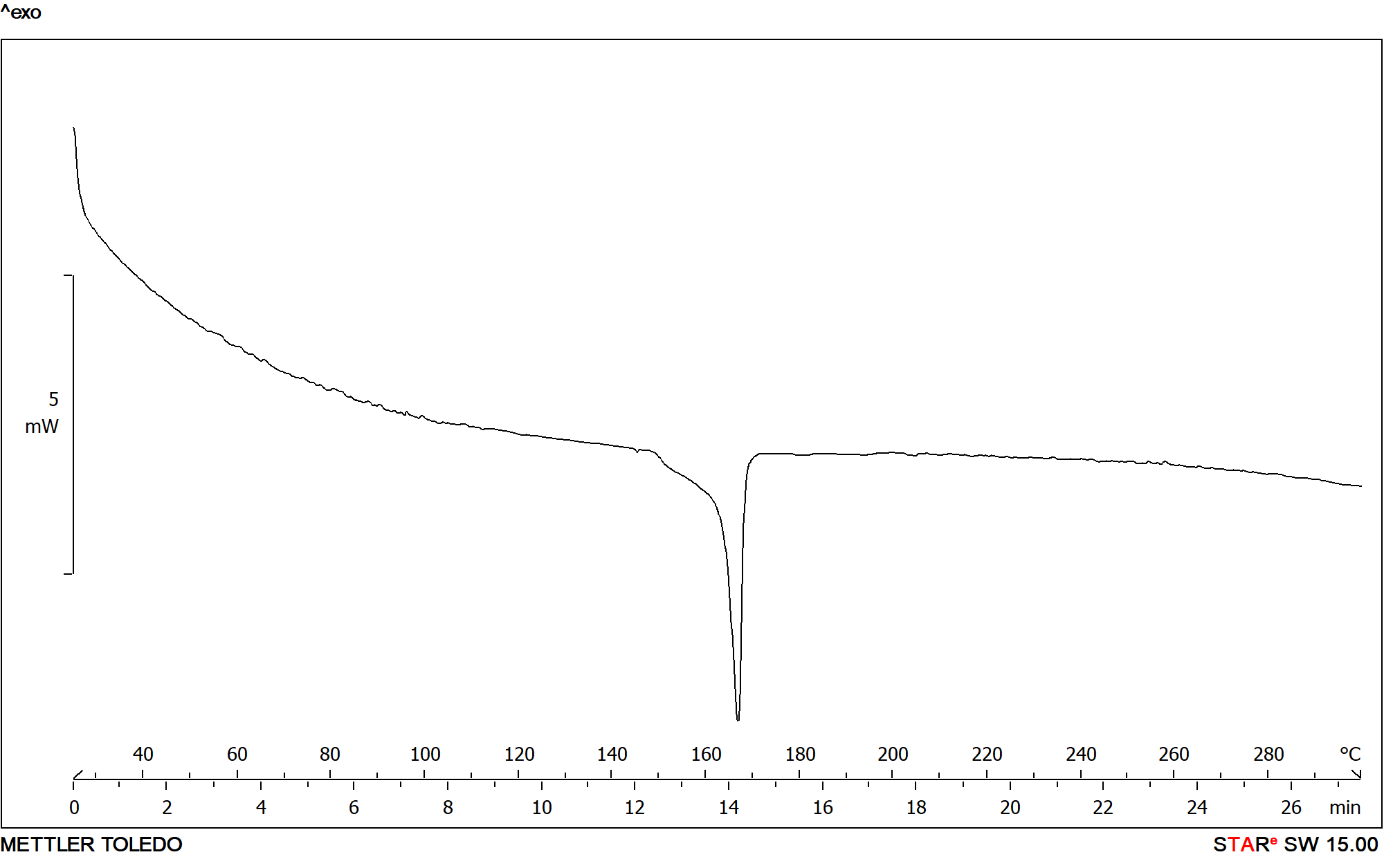


**Figure S52.** DSC curve of **IazH**.


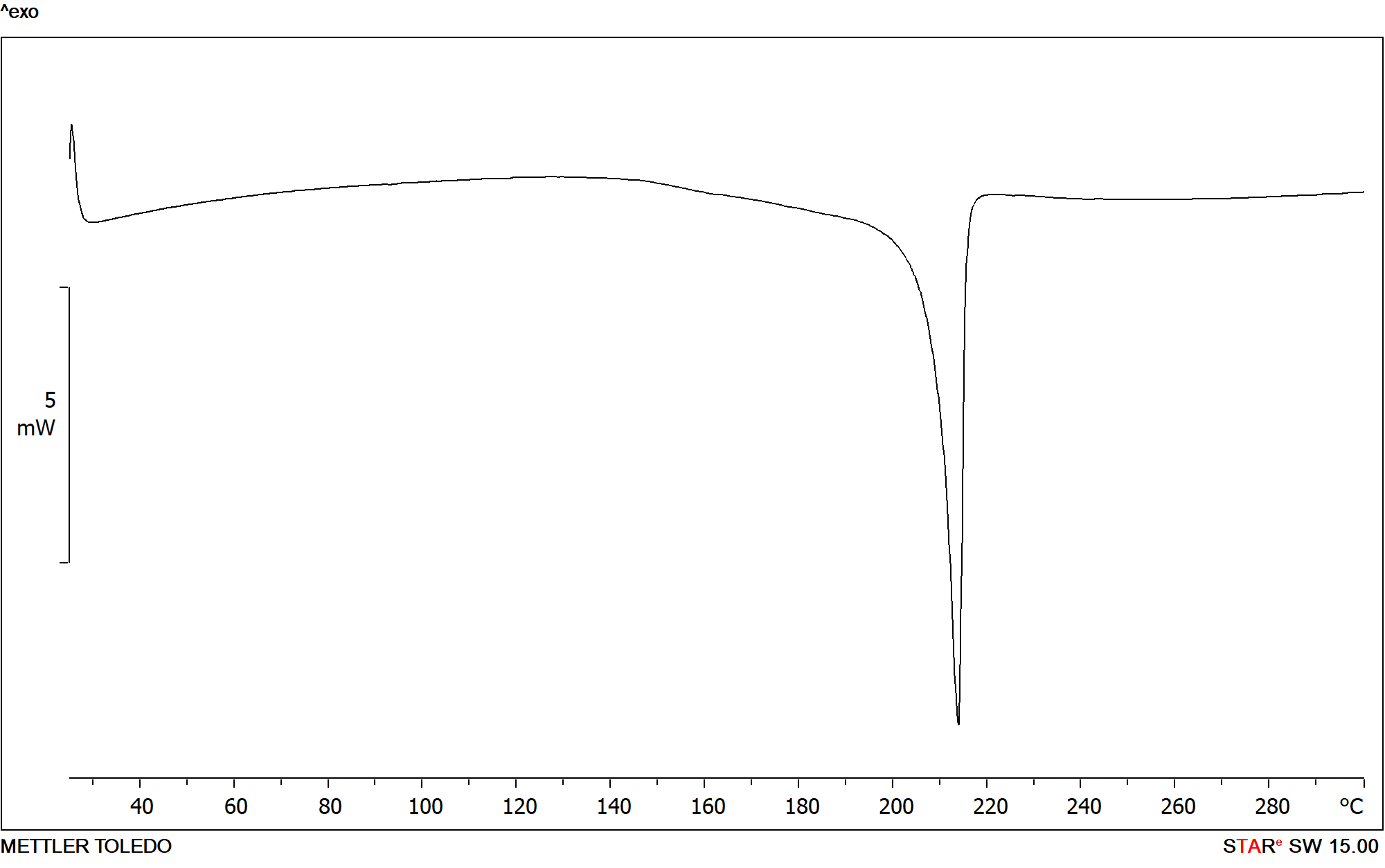


**Figure S53.** DSC curve of **IazCN**.


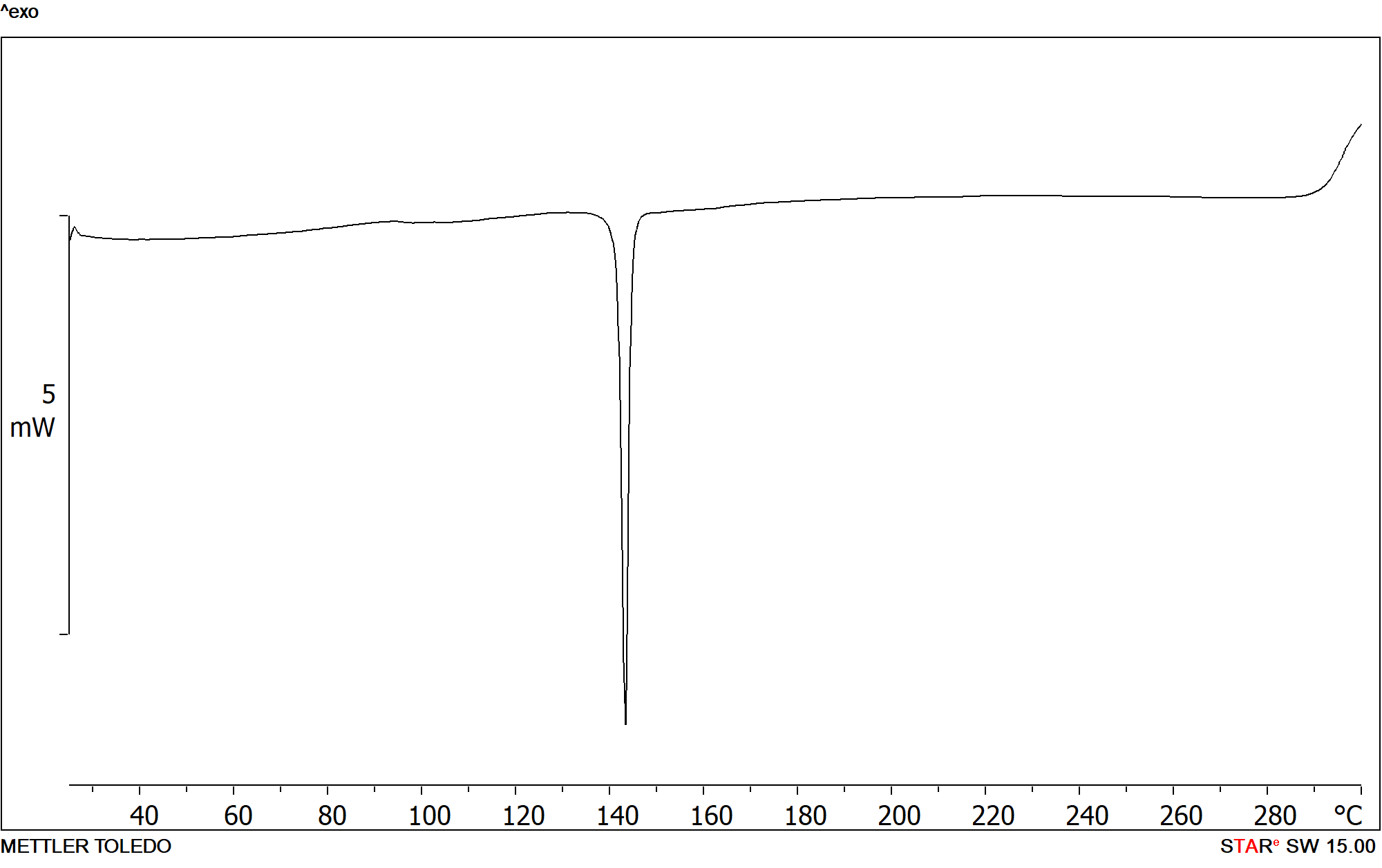


**Figure S54.** DSC curve of **IazCl**.


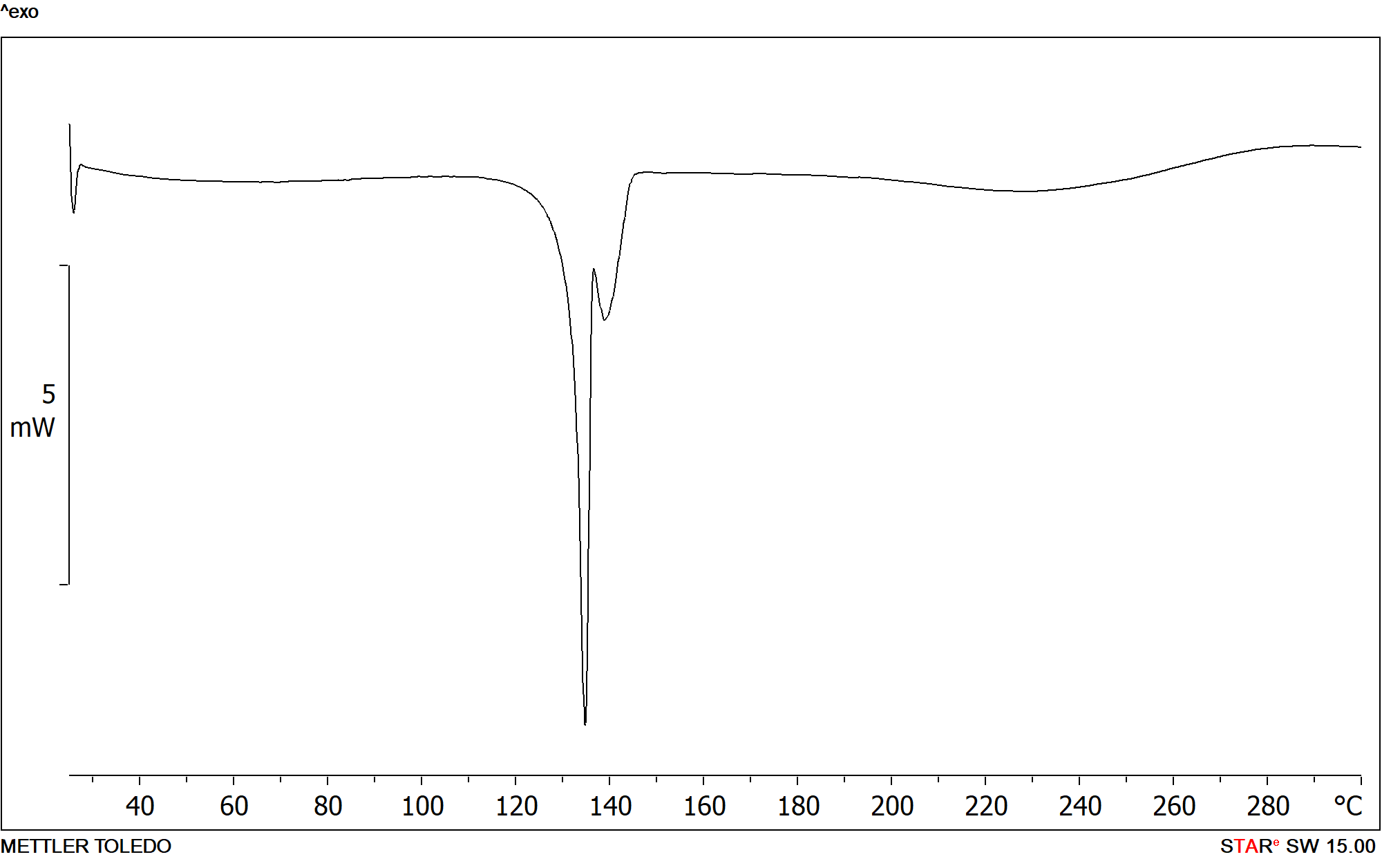


**Figure S55.** DSC curve of (**IazCl**)(**44bpy**).


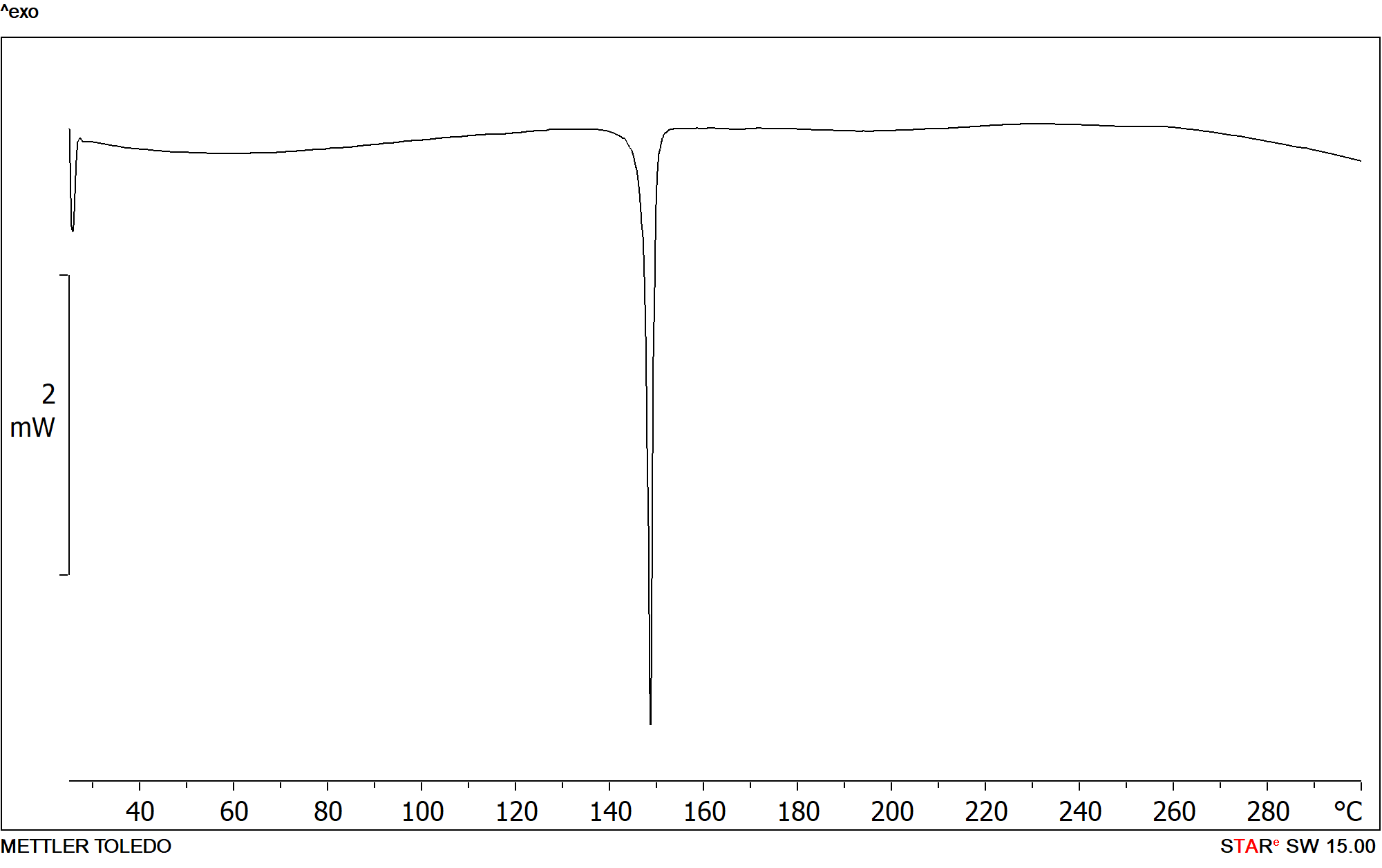


**Figure S56.** DSC curve of (**IazCl**)_2_(**44azpy**).


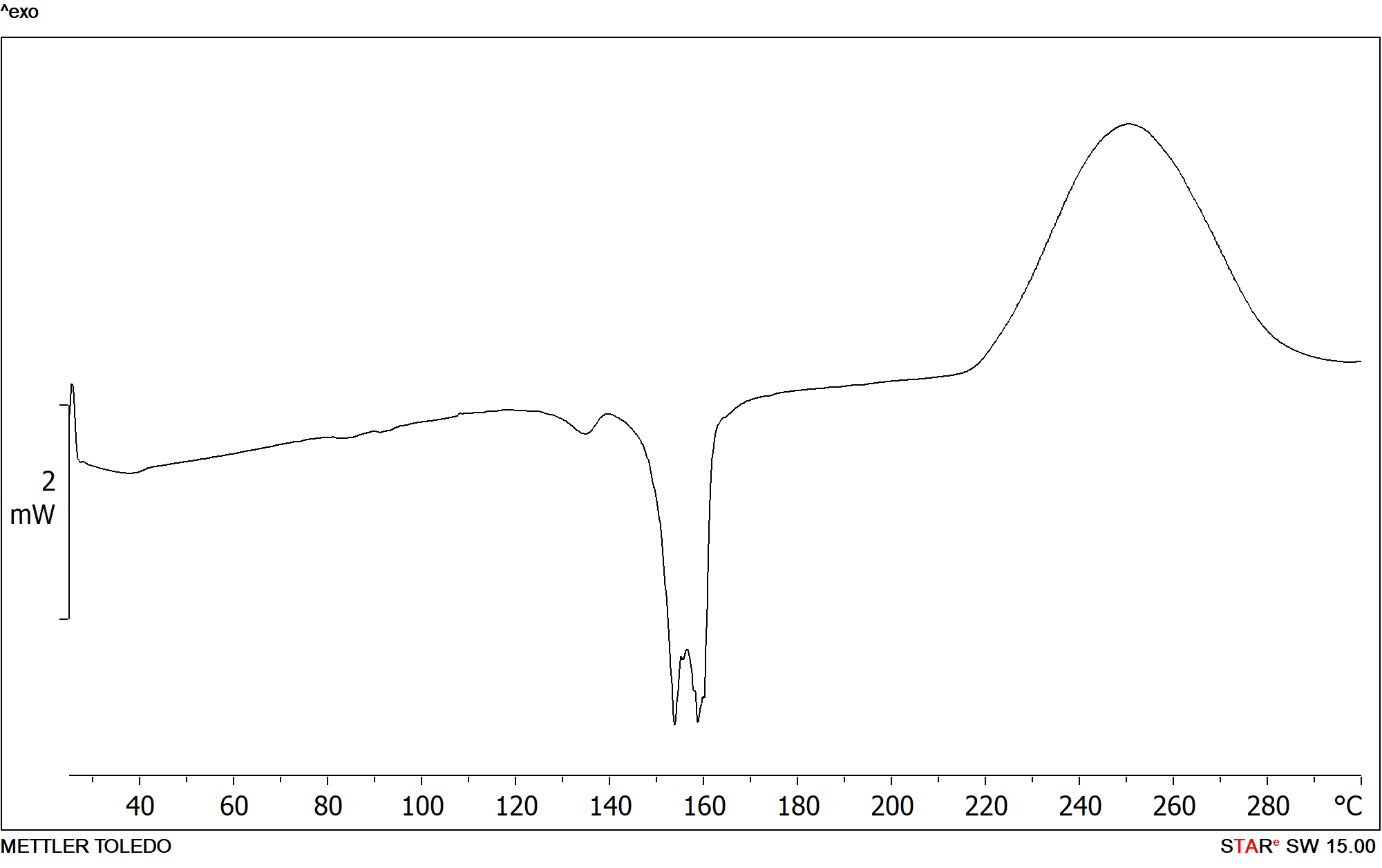


**Figure S57.** DSC curve of (**IazCl**)_2_(**dpe**).


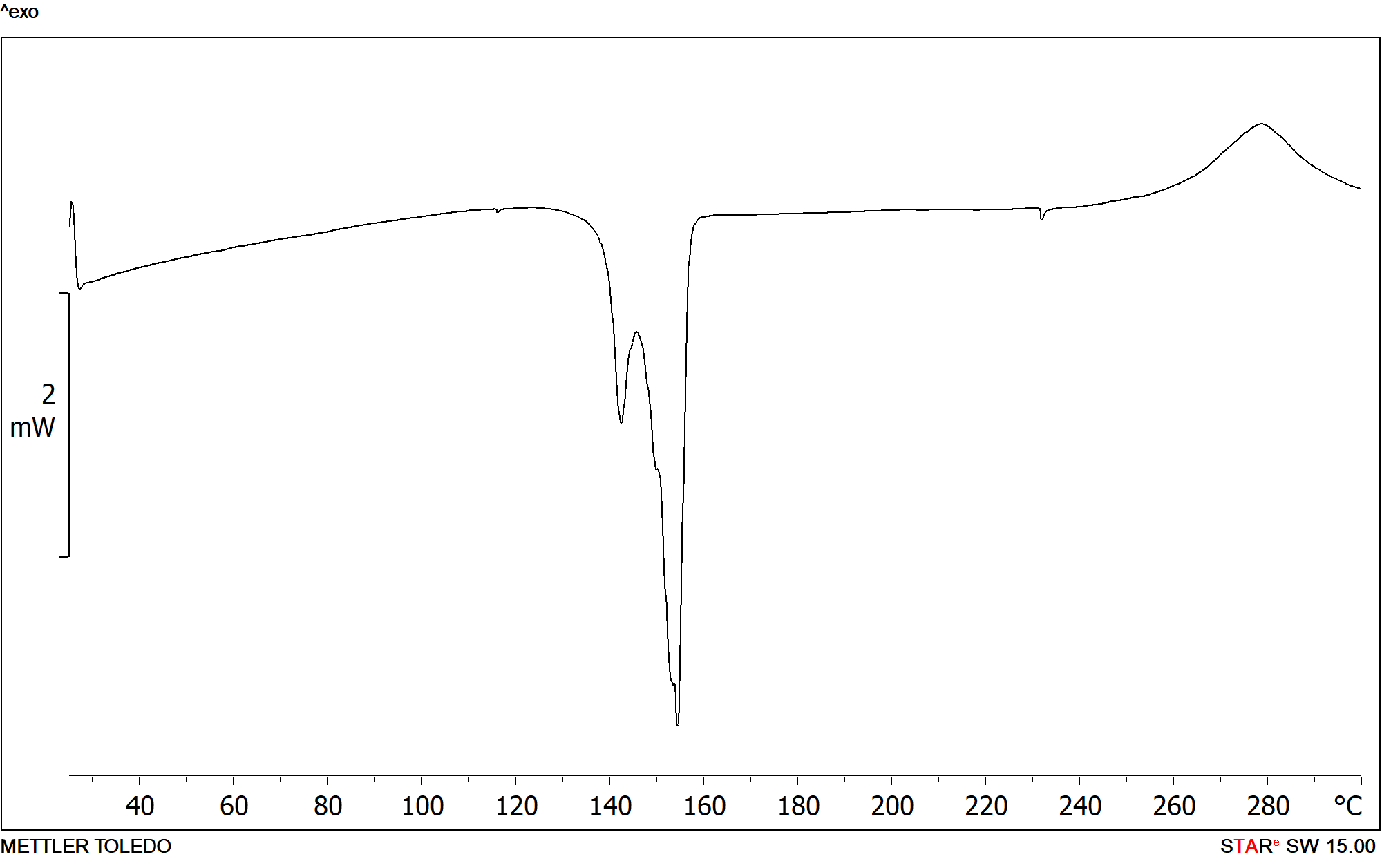


**Figure S58.** DSC curve of (**IazCl**)_2_(**hpy**).


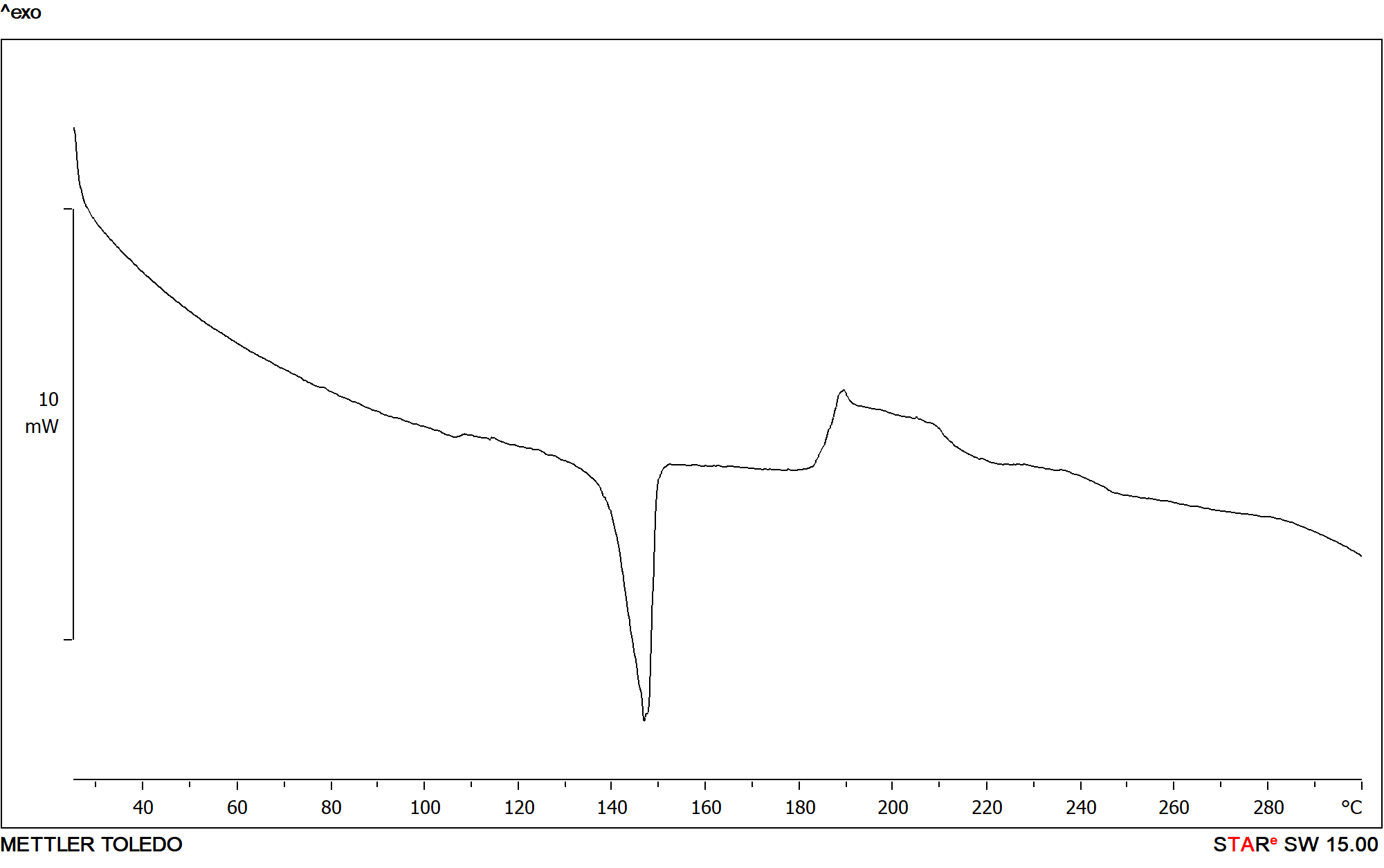


**Figure S59.** DSC curve of (**IazCl**)_2_(**dpa**).


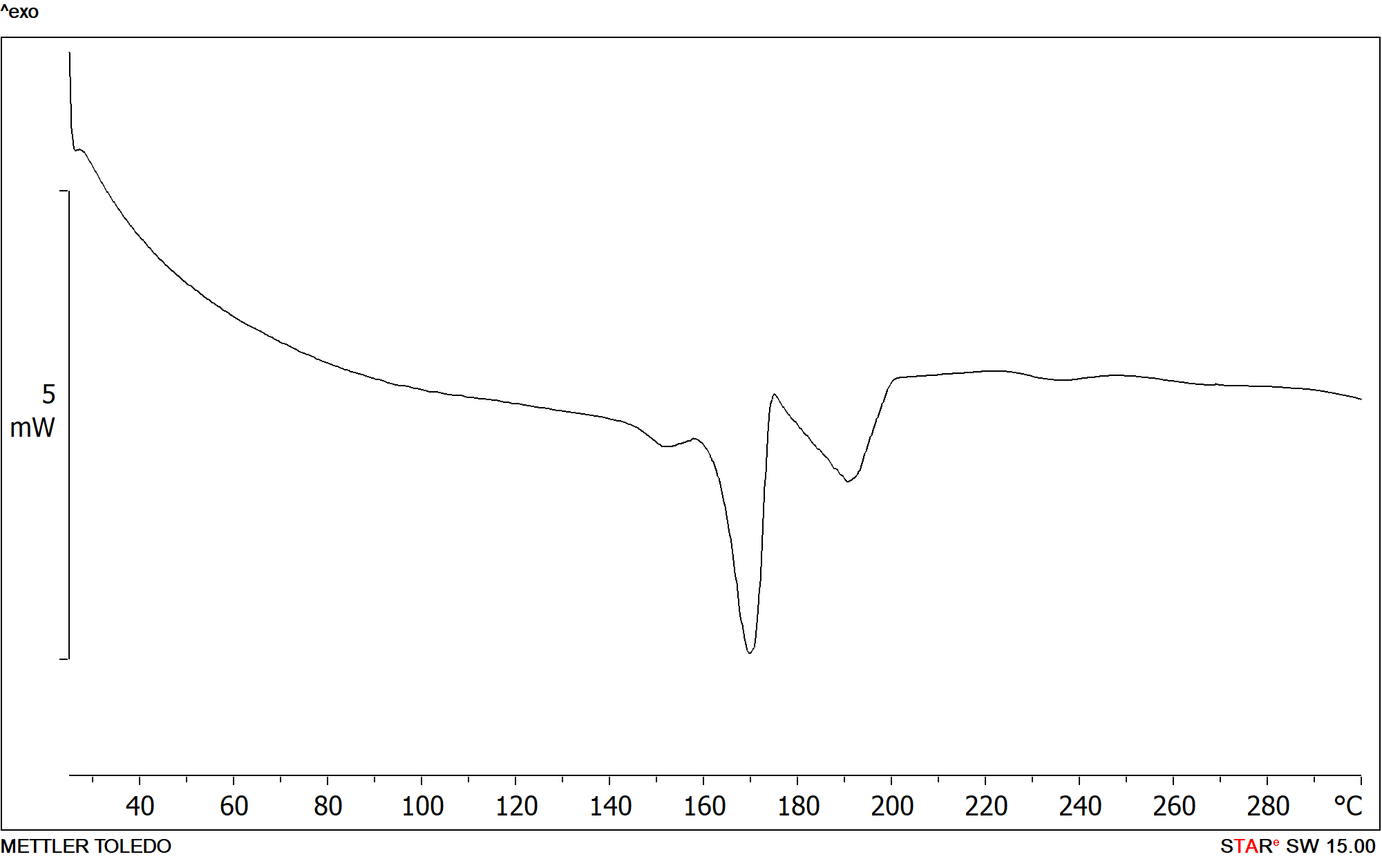


**Figure S60.** DSC curve of (**IazCN**)_2_(**44azpy**).


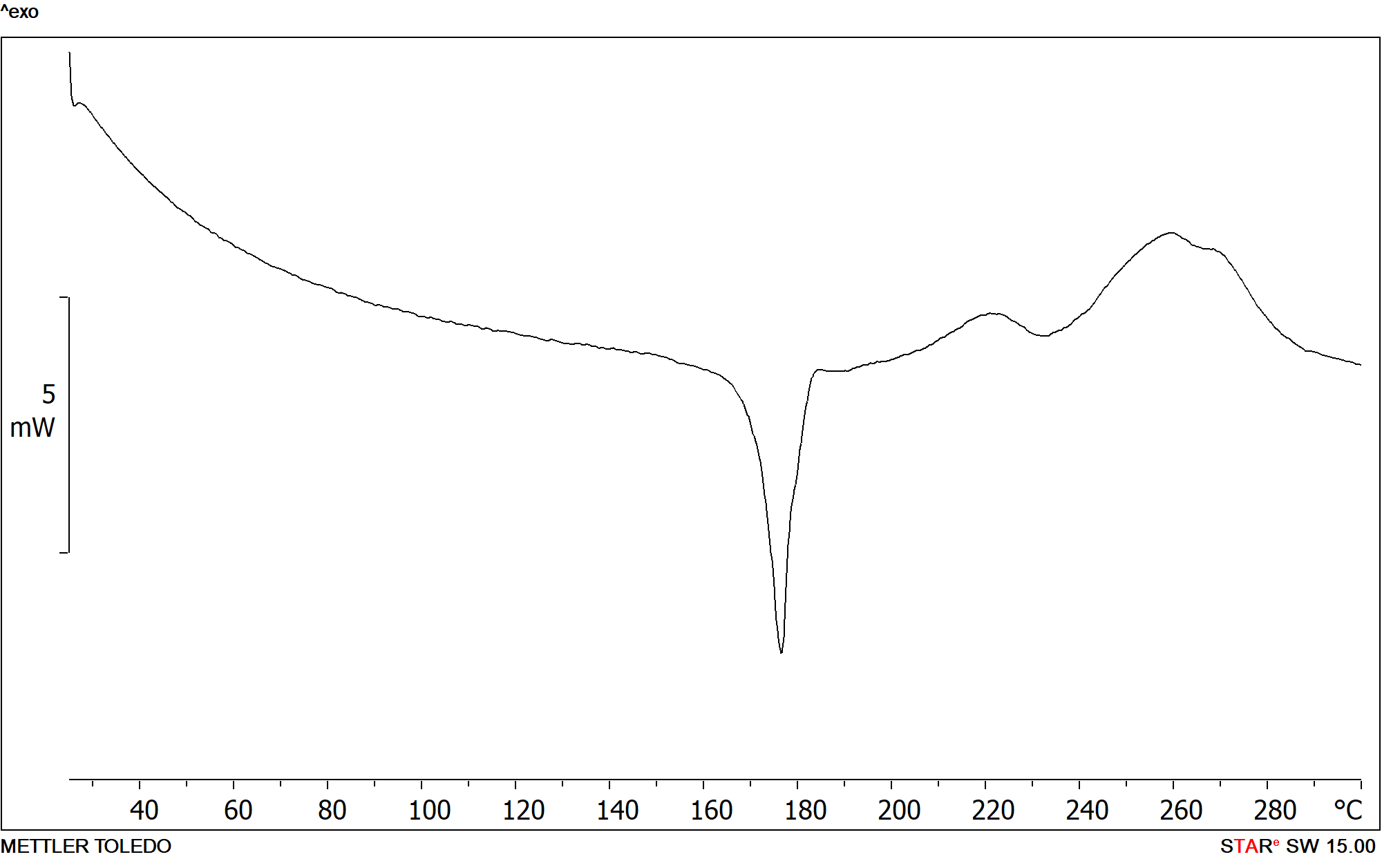


**Figure S61.** DSC curve of (**IazCN**)_2_(**dpe**).


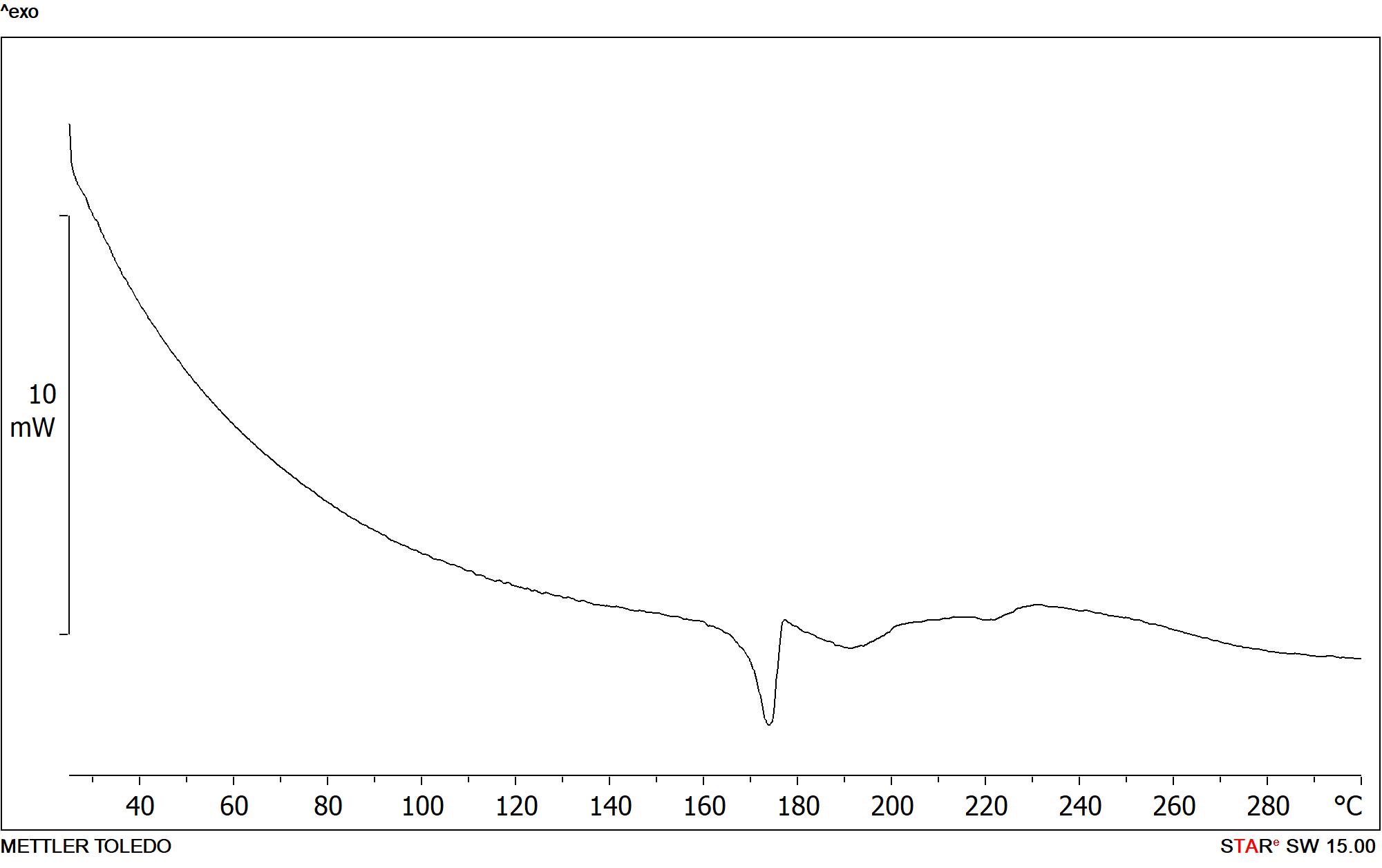


**Figure S62.** DSC curve of (**IazCN**)_2_(**hpy**).


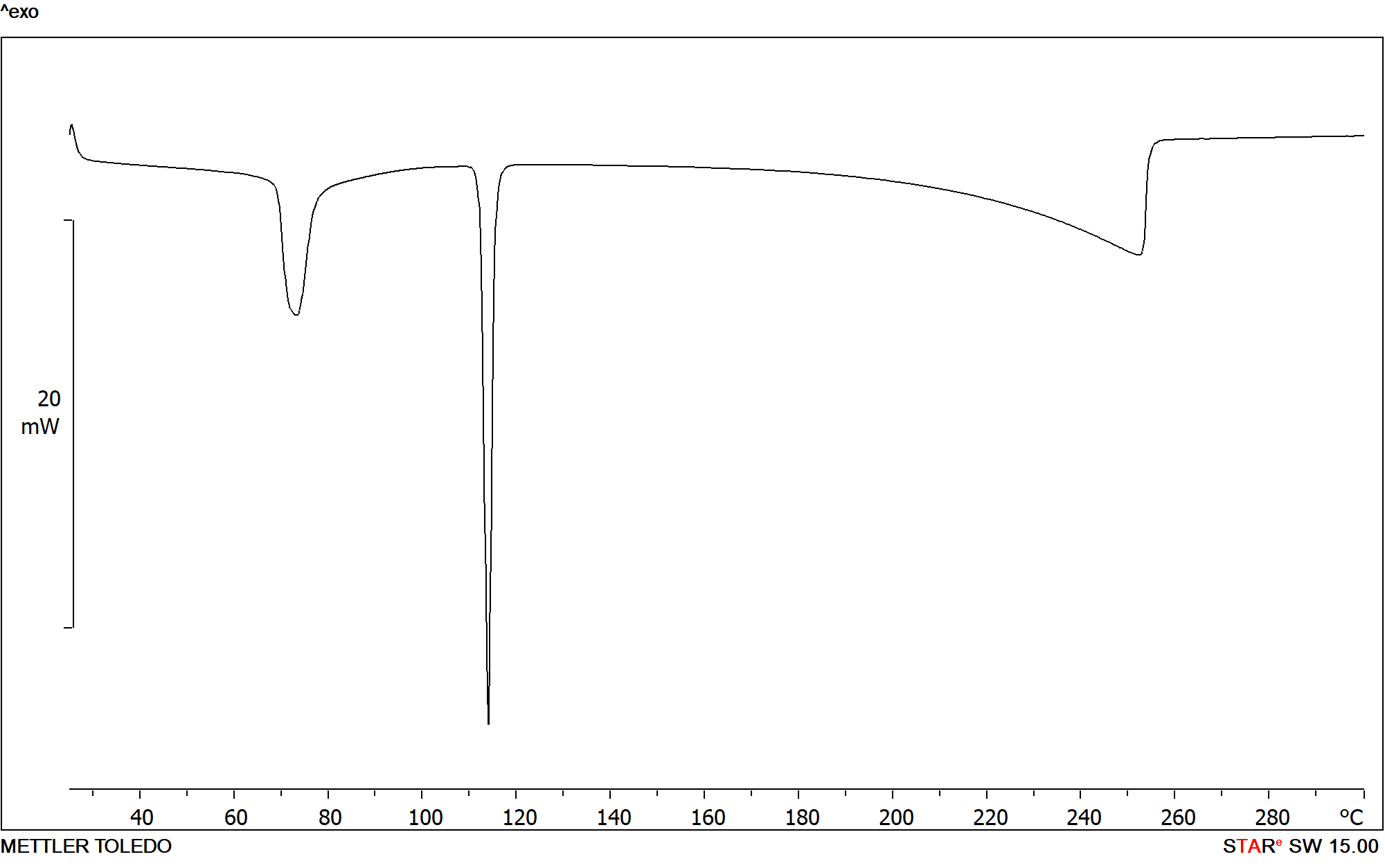


**Figure S63.** DSC curve of **44bpy**.


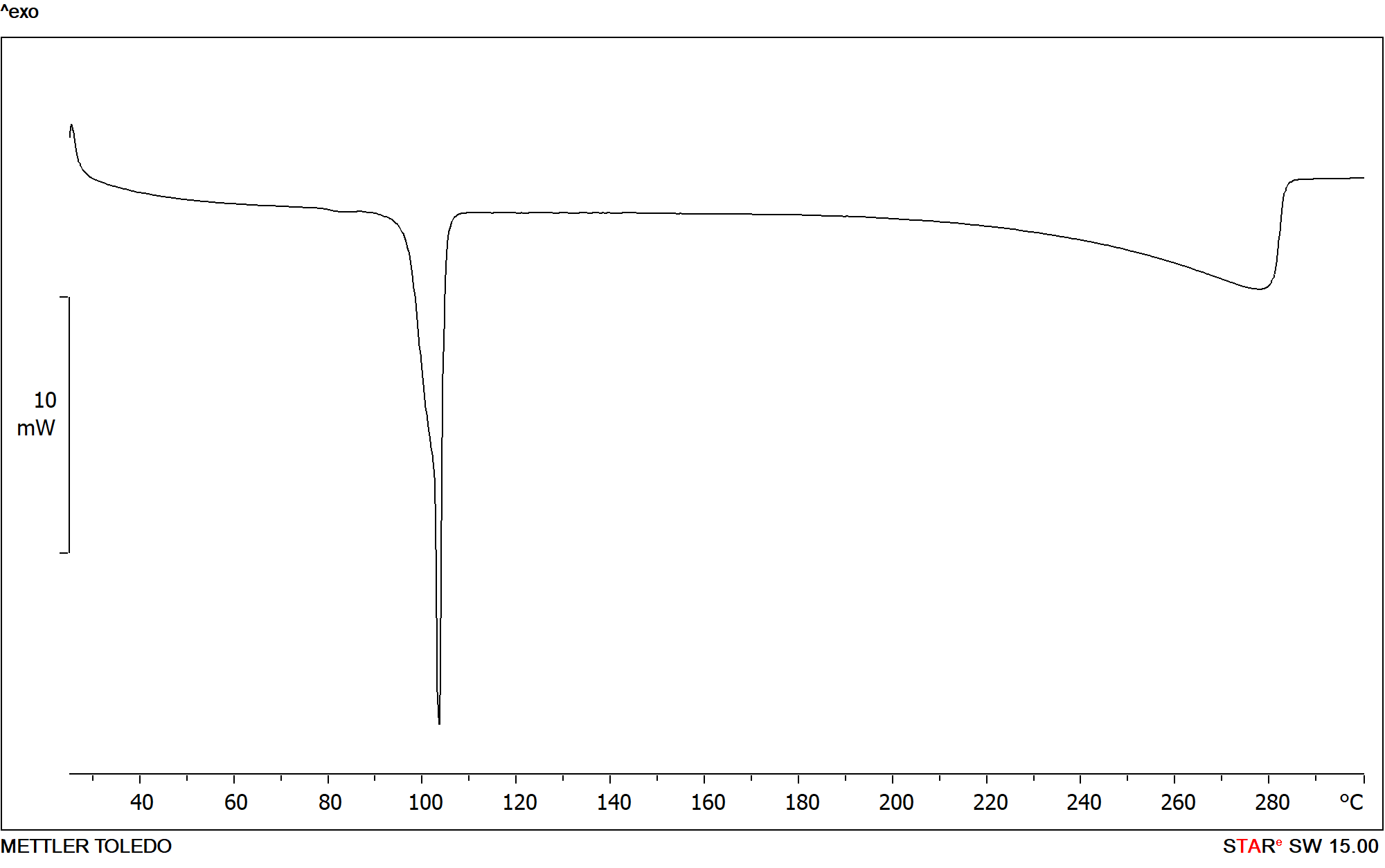


**Figure S64.** DSC curve of **44azpy**.


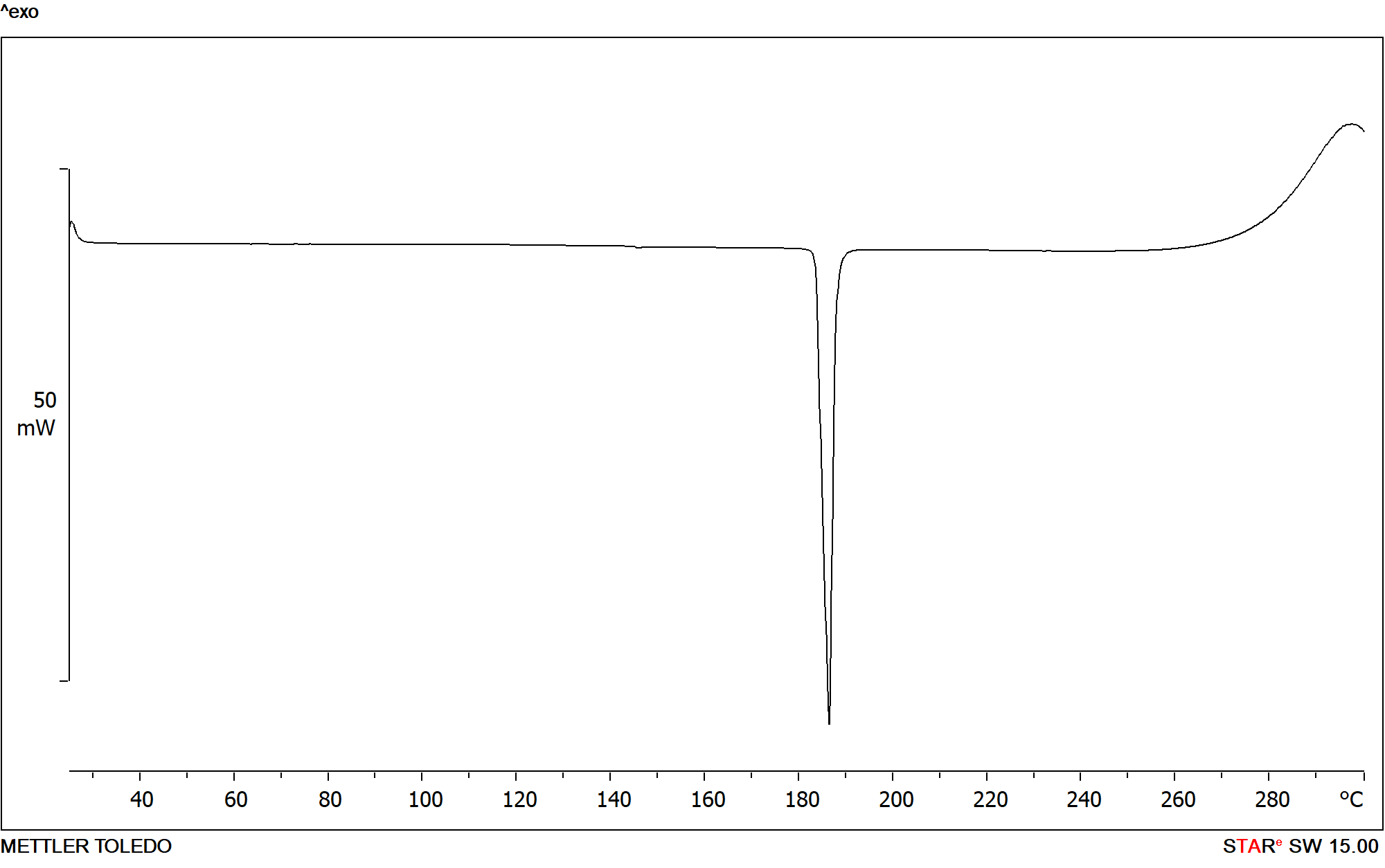


**Figure S65.** DSC curve of **hpy**.


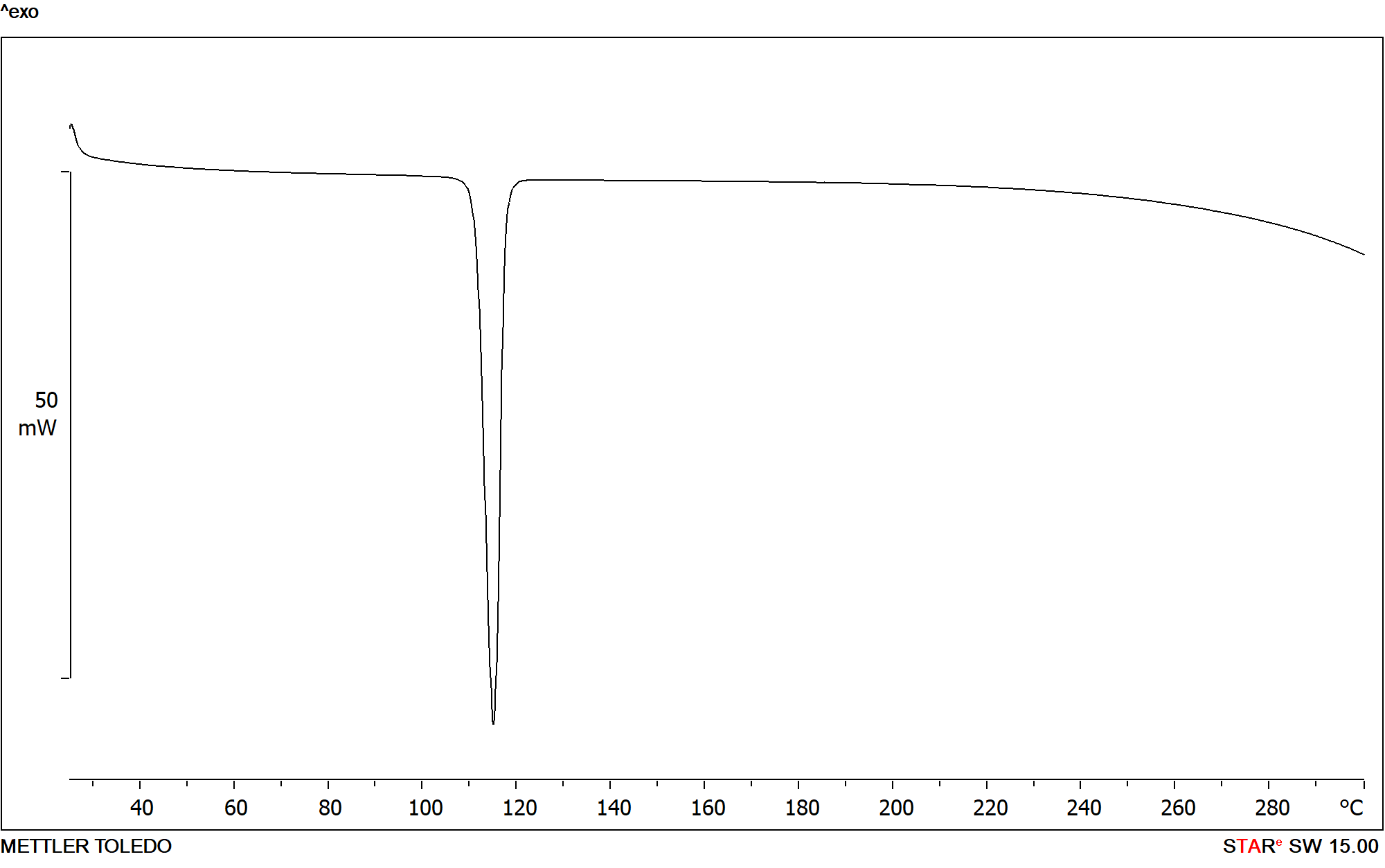


**Figure S66.** DSC curve of **dpa**.


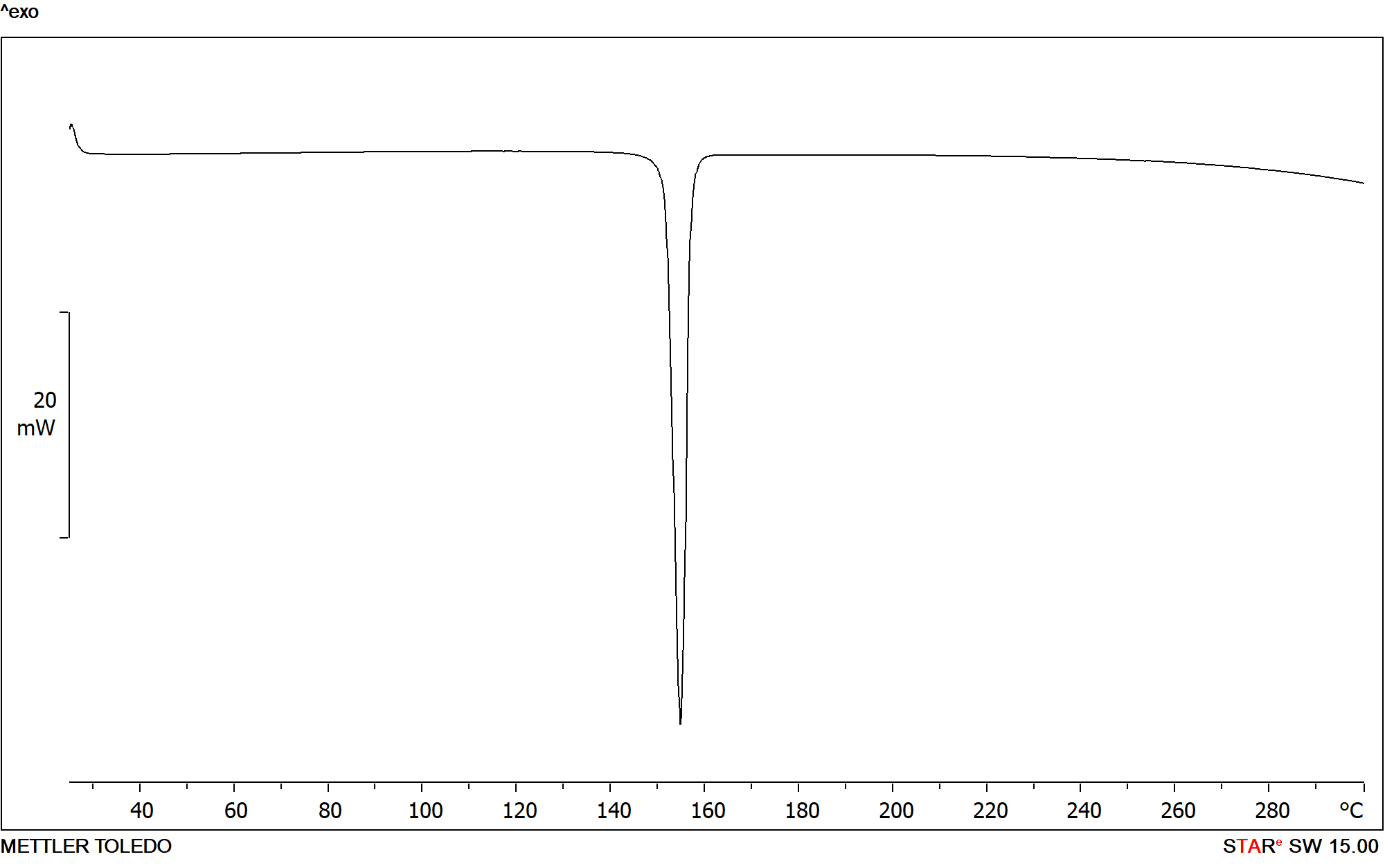


**Figure S67.** DSC curve of **dpe**.

**Table S4.** Percentages for individual intermolecular interactions of **IazCl** and **IazCN** molecules in cocrystals with five bipyridyl acceptors


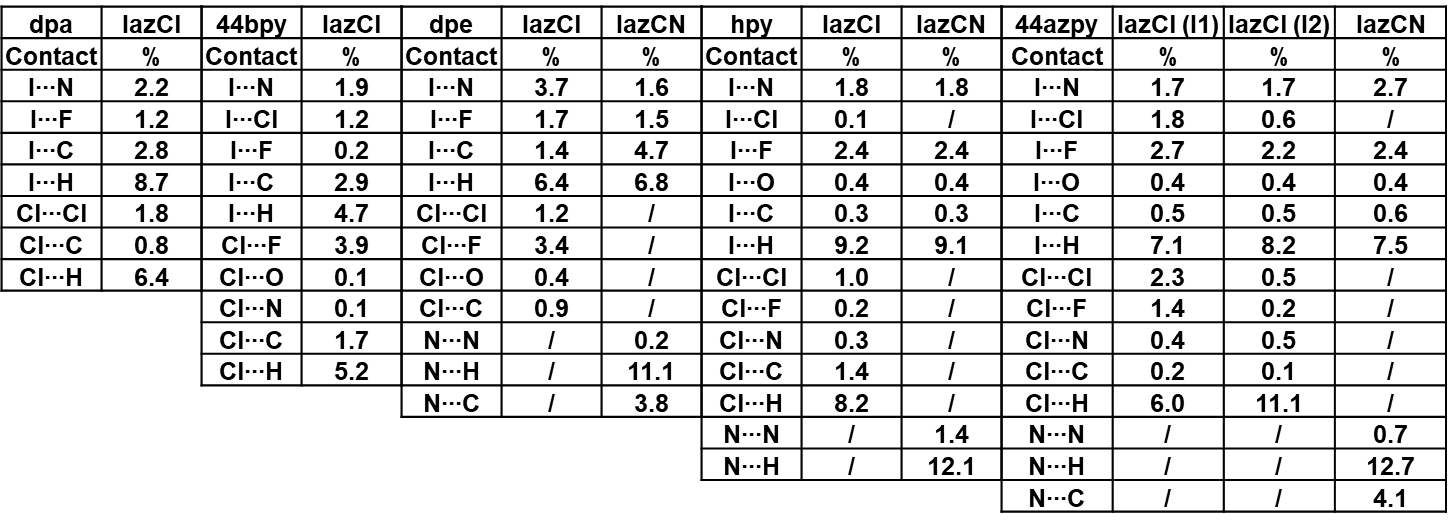


**Table S5.** Halogen bond, halogen contact and hydrogen bond lengths, angles and distances in the prepared cocrystals.

| cocrystal | D∙∙∙A | *d* (D∙∙∙A) or *d* (X∙∙∙A) / Å | ∠ (D–H∙∙∙A) or ∠ (D–X∙∙∙A) / ° |
| --- | --- | --- | --- |
| (**IazCl**)(**44bpy**) | C16–I1∙∙∙N3 | 2.832 | 176.8 |
|  | C9–H9∙∙∙N4 | 3.451 | 150.8 |
|  | C5–H5∙∙∙F4 | 3.244 | 131.3 |
|  | C23–H23∙∙∙C3 | 3.711 | 161.6 |
| (**IazCl**)_2_(**44azpy**) | C16–I1∙∙∙N5 | 2.903 | 172.1 |
|  | C34–I2∙∙∙N8 | 2.923 | 170.6 |
|  | C4–Cl1∙∙∙Cl1 | 3.343 | 143.6 |
|  | C6–H6∙∙∙F2 | 3.302 | 149.6 |
|  | C26–H26∙∙∙F5 | 3.163 | 141.4 |
|  | C29–H29∙∙∙F3 | 3.436 | 153.9 |
| (**IazCl**)_2_(**dpe**) | C16–I1∙∙∙N3 | 2.801 | 178.3 |
|  | C4–Cl1∙∙∙Cl1 | 3.440 | 147.9 |
|  | C6–H6∙∙∙F3 | 3.476 | 154.6 |
|  | C9–H9∙∙∙F4 | 3.343 | 142.1 |
|  | C20–H20∙∙∙F2 | 3.315 | 146.3 |
|  | C22–H22∙∙∙F1 | 3.376 | 156.2 |
| (**IazCl**)_2_(**dpa**) | C16–I1∙∙∙N3 | 2.876 | 175.0 |
|  | C4–Cl1∙∙∙Cl1 | 3.470 | 151.5 |
| (**IazCl**)_2_(**hpy**) | C16–I1∙∙∙N3 | 2.867 | 169.9 |
|  | C8–H8∙∙∙F4 | 3.060 | 135.1 |
|  | C11–H11∙∙∙F2 | 3.513 | 159.1 |
| (**IazCN**)_2_(**44azpy**) | C17–I1∙∙∙N4 | 2.922 | 171.4 |
|  | C3–H3∙∙∙N3 | 3.441 | 148.9 |
|  | C9–H9∙∙∙F4 | 3.119 | 142.6 |
|  | C12–H12∙∙∙F2 | 3.439 | 159.0 |
| (**IazCN**)_2_(**dpe**) | C17–I1∙∙∙N4 | 2.952 | 175.4 |
|  | C3–H3∙∙∙N3 | 3.434 | 155.9 |
|  | C24–H24∙∙∙N3 | 3.593 | 171.6 |
|  | C10–H10∙∙∙F1 | 3.359 | 147.0 |
|  | C20–H20∙∙∙F2 | 3.381 | 149.5 |
| (**IazCN**)_2_(**hpy**) | C17–I1∙∙∙N4 | 2.851 | 169.9 |
|  | C8–H8∙∙∙F4 | 3.060 | 135.1 |
|  | C11–H11∙∙∙F2 | 3.513 | 159.1 |


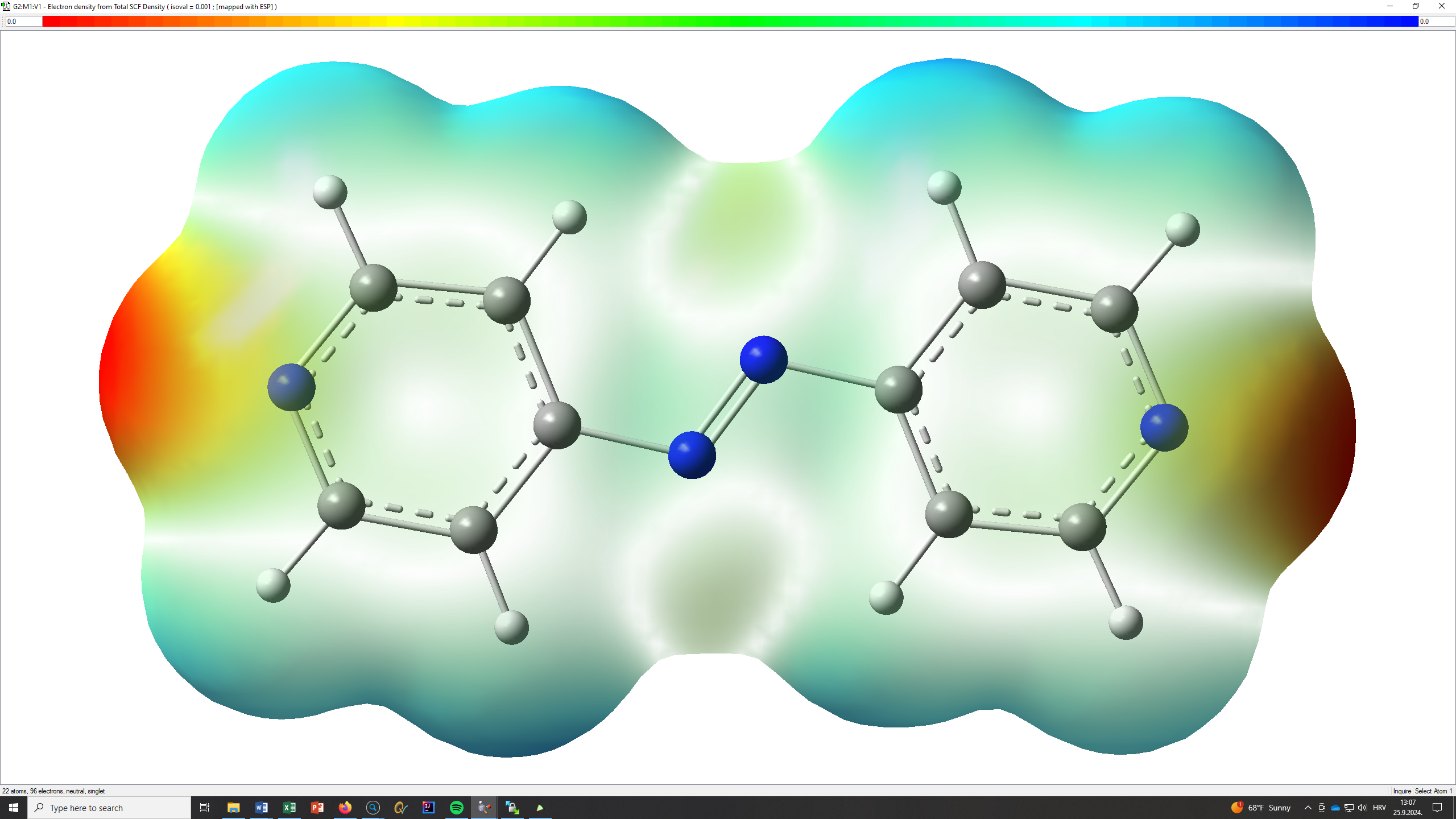


**Figure S68**. Molecular electrostatic potential map of 44**azpy**. MEP_min_ = −134.2 kJ mol^−1^ *e*^−1^


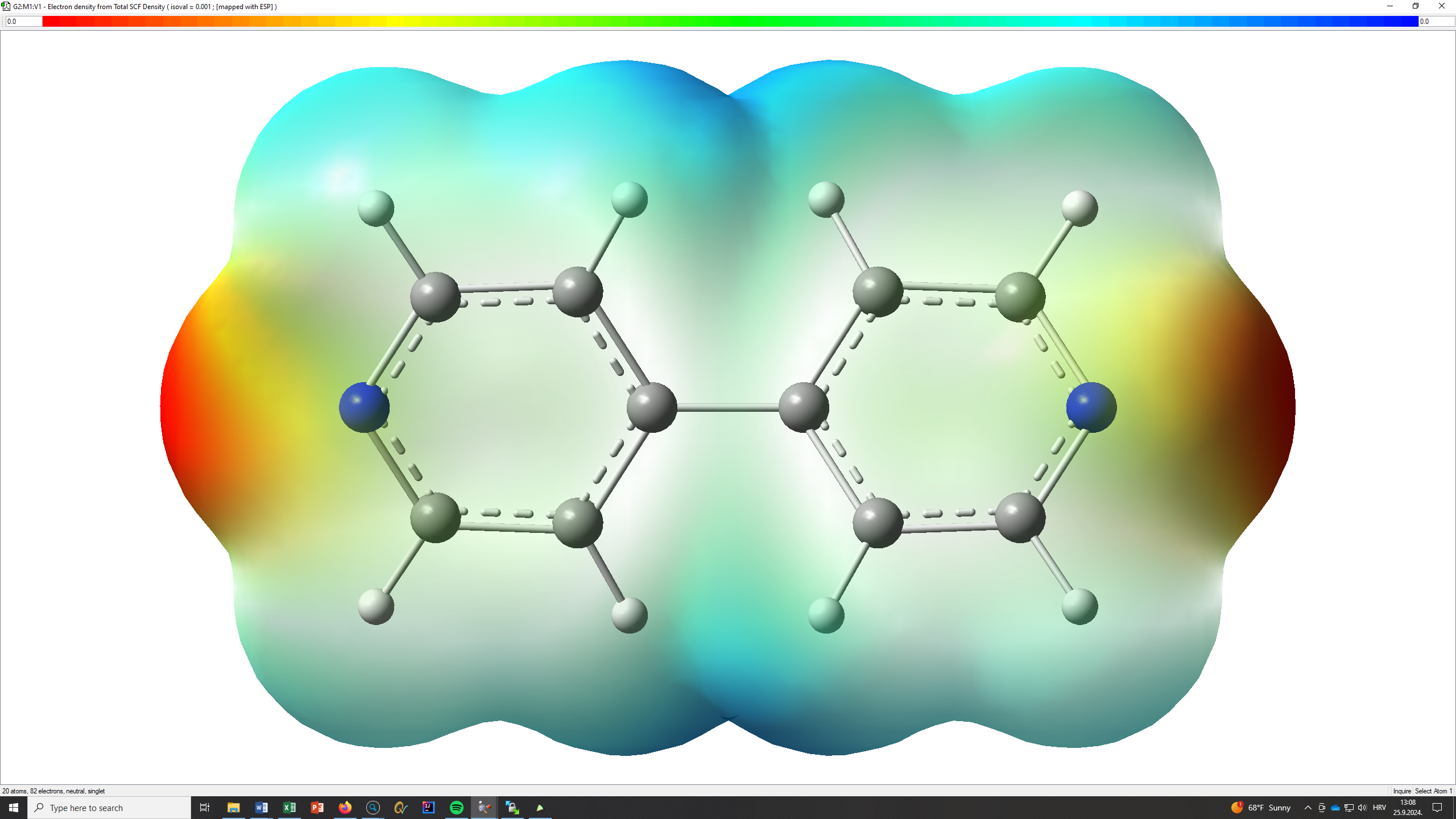


**Figure S69**. Molecular electrostatic potential map of 44**bpy**. MEP_min_ = −138.9 kJ mol^−1^ *e*^−1^


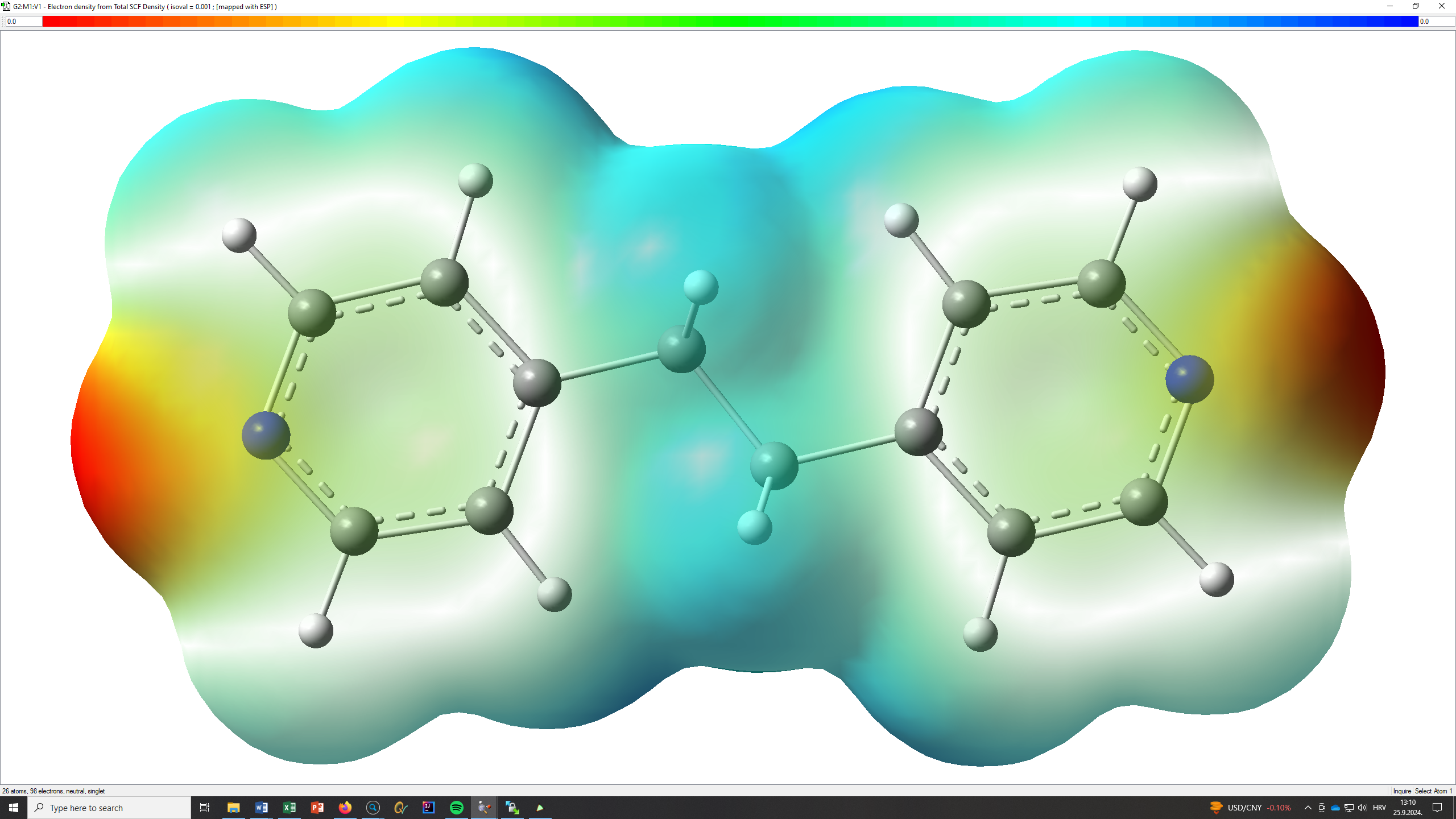


**Figure S70**. Molecular electrostatic potential map of **dpa**. MEP_min_ = −148.5 kJ mol^−1^ *e*^−1^


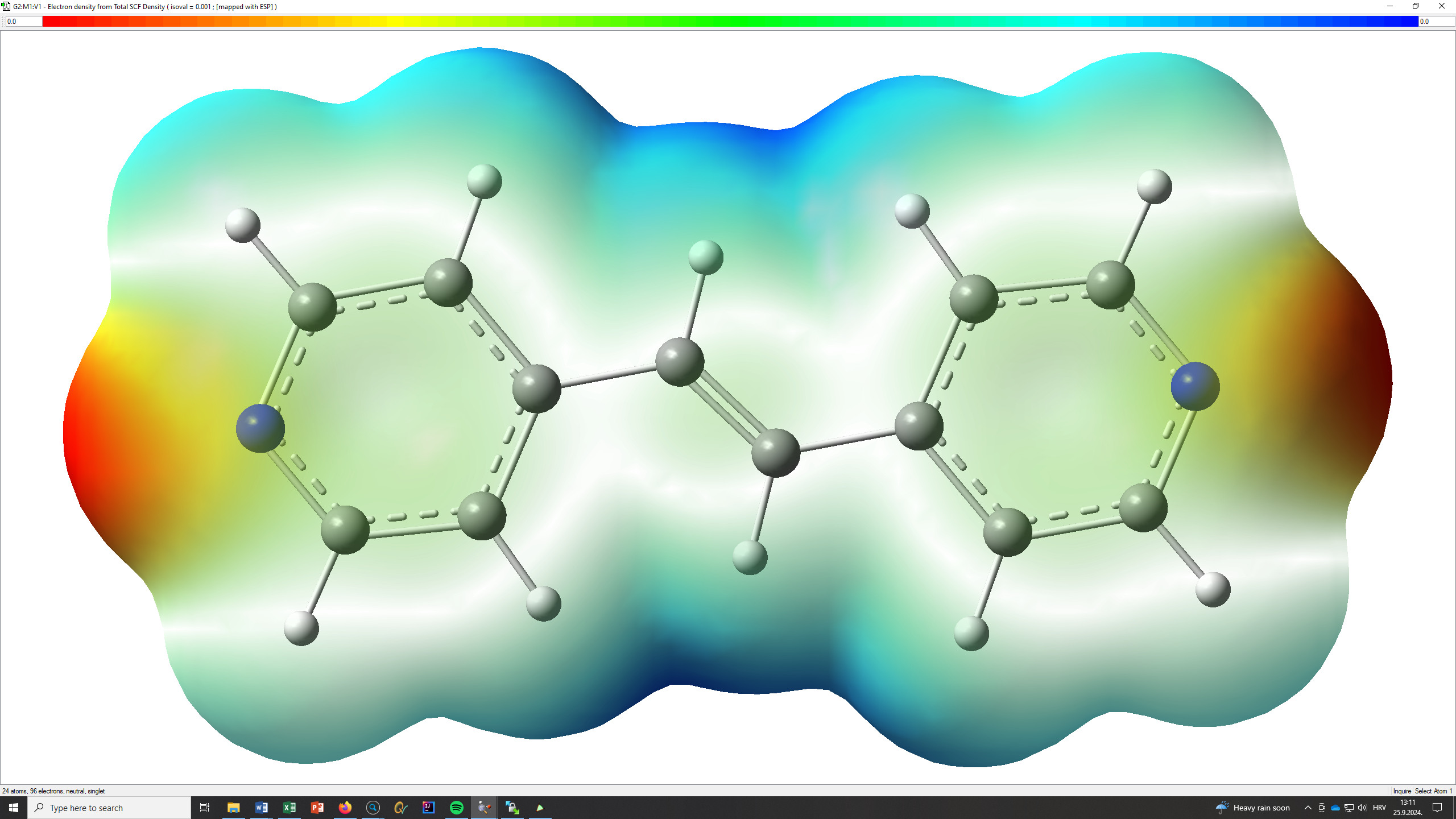


**Figure S71**. Molecular electrostatic potential map of **dpe**. MEP_min_ = −143.4 kJ mol^−1^ *e*^−1^


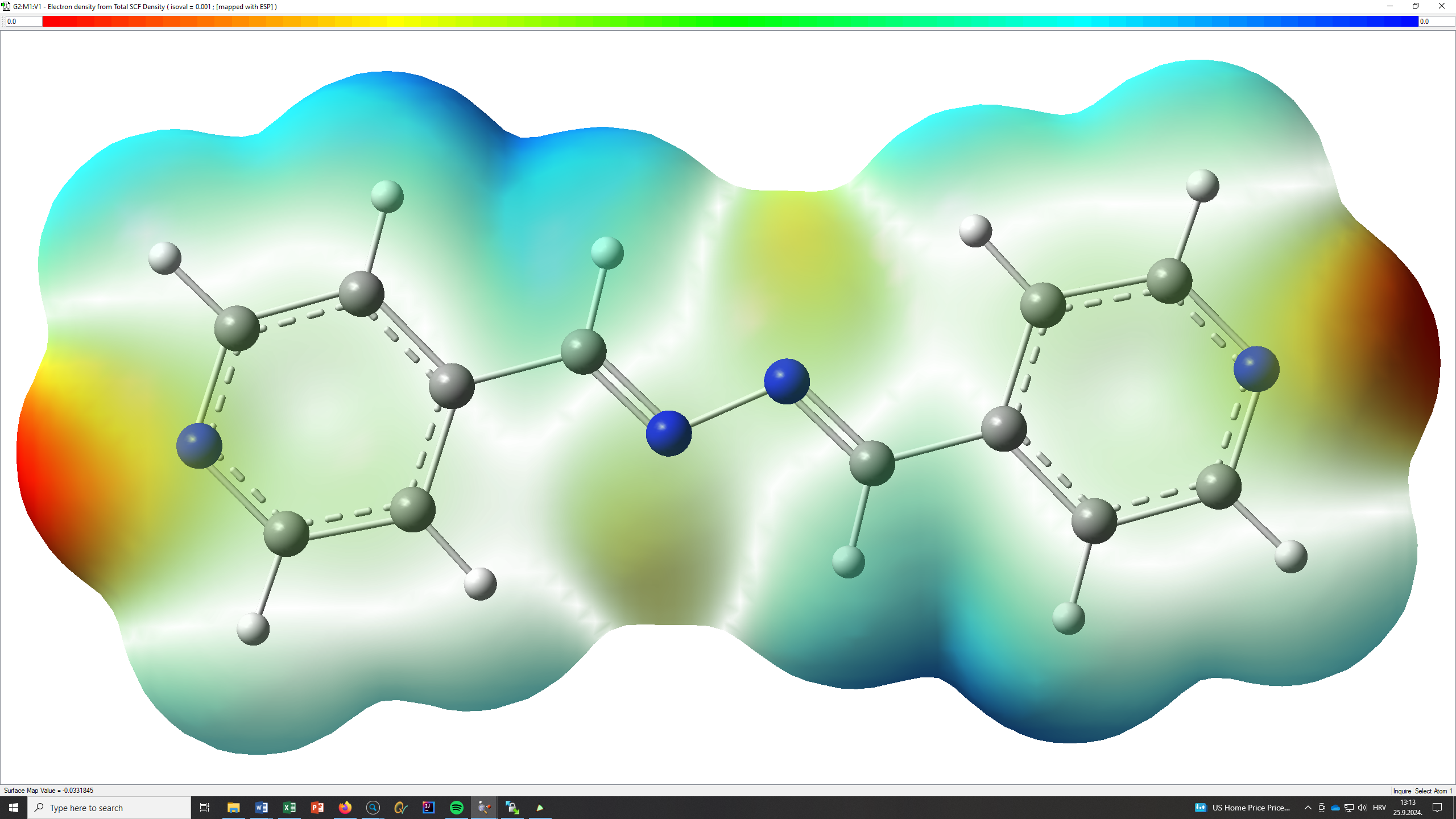


**Figure S72**. Molecular electrostatic potential map of **hpy**. MEP_min_ = −138.1 kJ mol^−1^ *e*^−1^


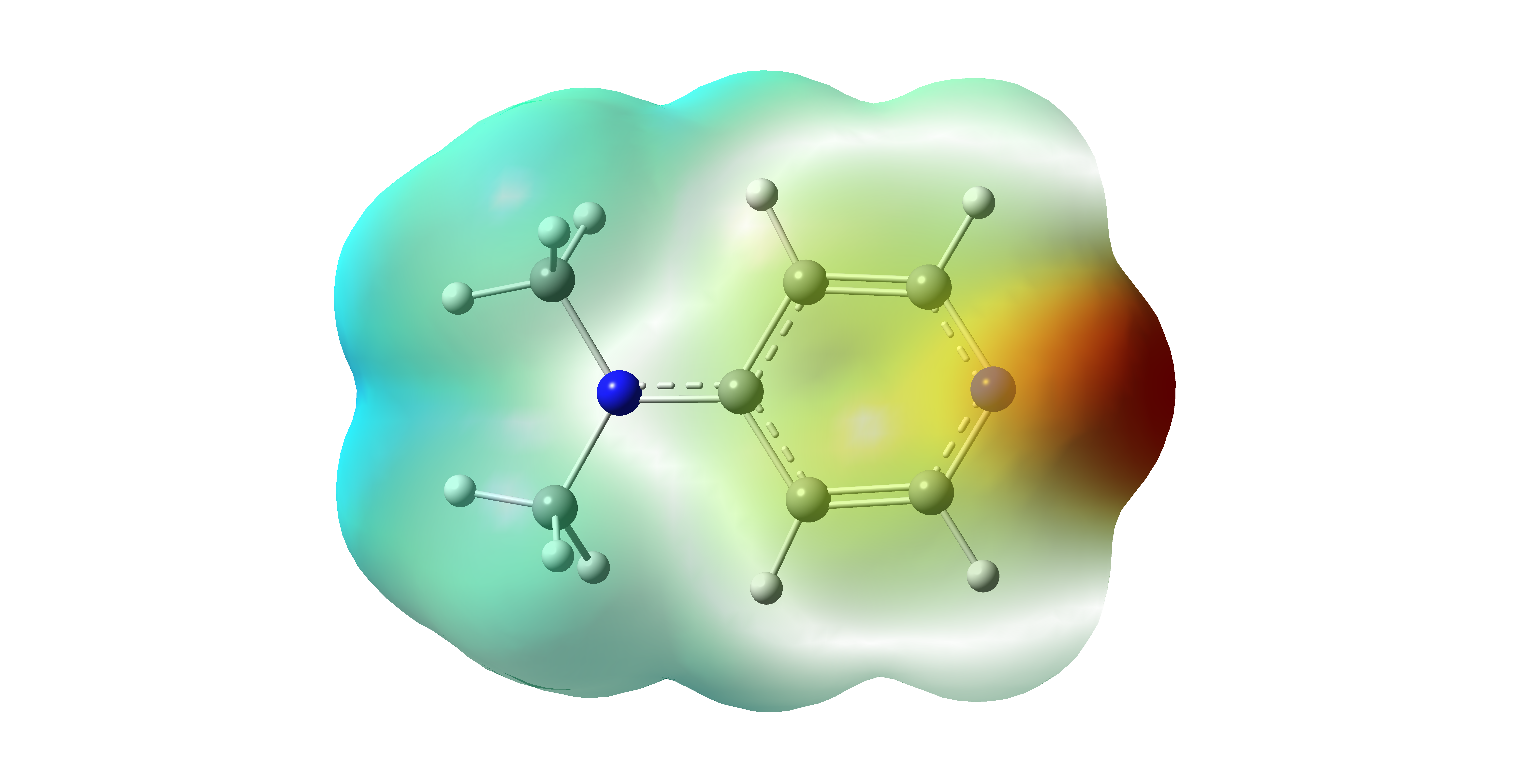


**Figure S73**. Molecular electrostatic potential map of **dmap**. MEP_min_ = −180.4 kJ mol^−1^ *e*^−1^


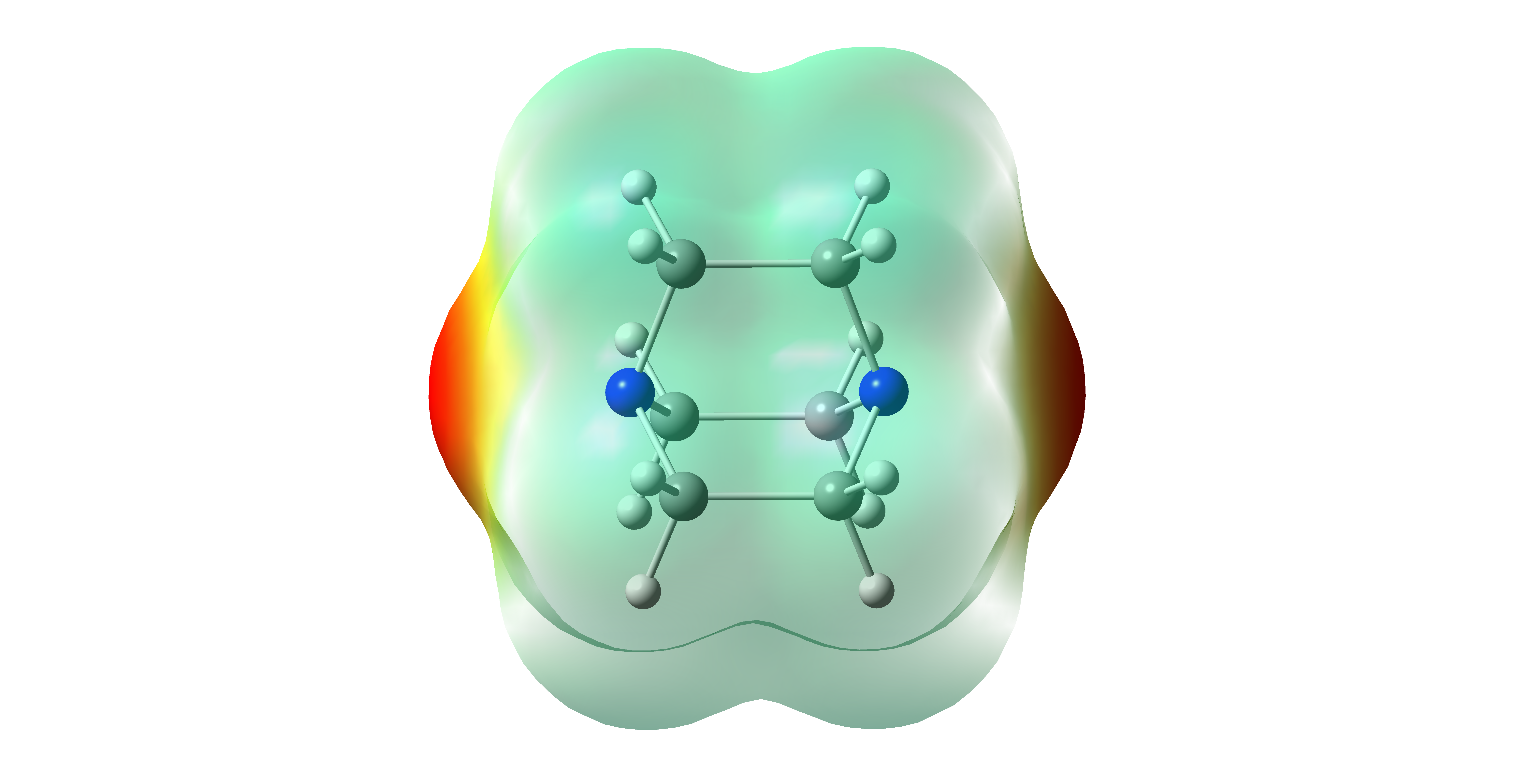


**Figure S74**. Molecular electrostatic potential map of **dabco**. MEP_min_ = −137.3 kJ mol^−1^ *e*^−1^


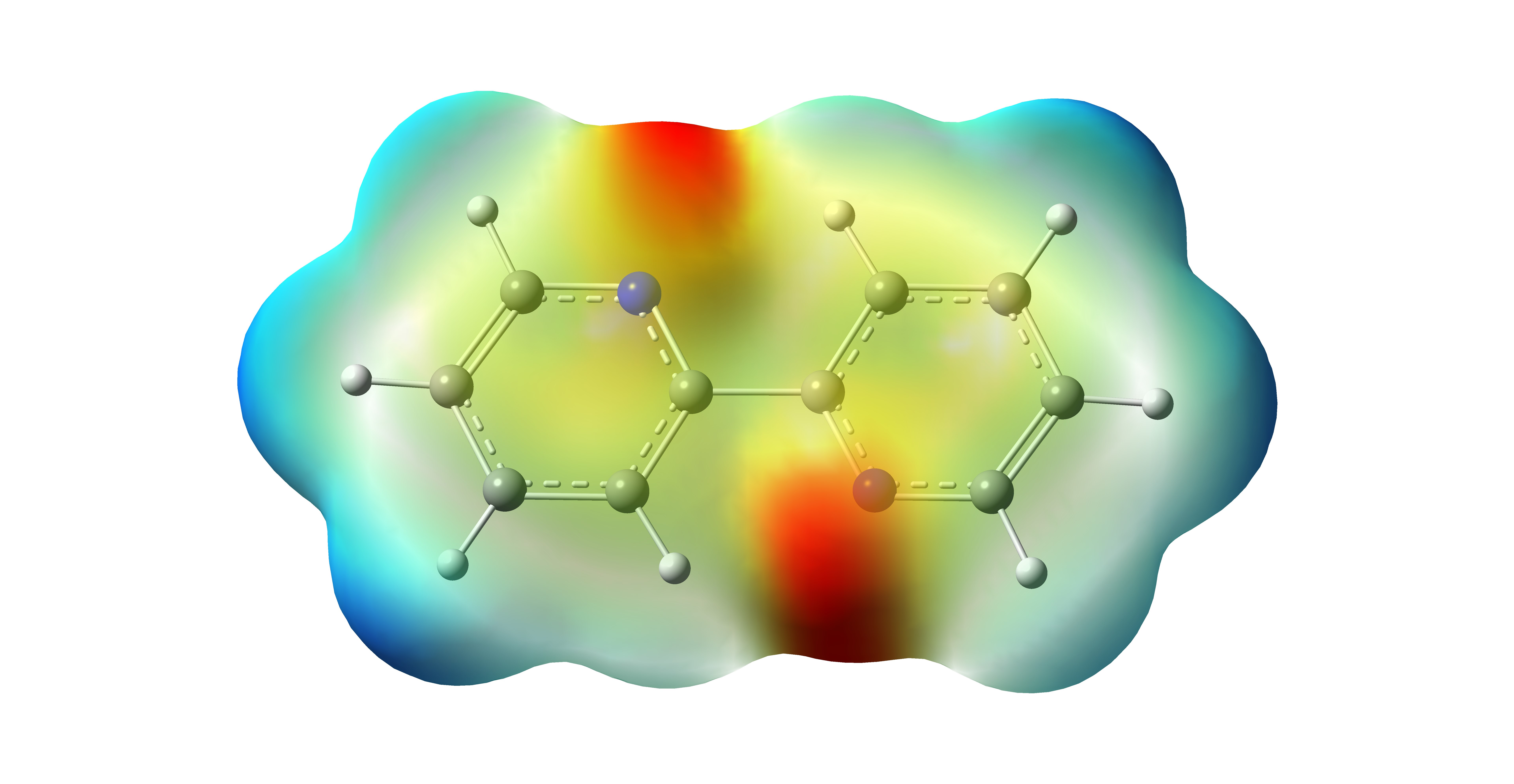


**Figure S75**. Molecular electrostatic potential map of **22bpy**. MEP_min_ = −100.6 kJ mol^−1^ *e*^−1^
